# Telmisartan is neuroprotective in a hiPSC-derived spinal microtissue model for C9orf72 ALS via inhibition of neuroinflammation

**DOI:** 10.1016/j.stemcr.2025.102535

**Published:** 2025-06-19

**Authors:** Berkiye Sonustun, Björn F. Vahsen, Mario Ledesma-Terrón, Zhuoning Li, Laura Tuffery, Nan Xu, Elizabeth L. Calder, Johannes Jungverdorben, Leslie Weber, Aaron Zhong, David G. Miguez, Mara Monetti, Ting Zhou, Elisa Giacomelli, Lorenz Studer

**Affiliations:** 1 Developmental Biology Program & Center for Stem Cell Biology, Memorial Sloan Kettering Cancer Center, New York, NY 10065, USA; 2 Weill Cornell Graduate School of Medical Sciences, Cornell University, New York, NY 10065, USA; 3 Oxford Motor Neuron Disease Centre, Nuffield Department of Clinical Neurosciences, University of Oxford, John Radcliffe Hospital, Oxford OX3 9DU, UK; 4 Kavli Institute for Nanoscience Discovery, University of Oxford, Dorothy Crowfoot Hodgkin Building, OX1 3QU Oxford, UK; 5 Centro de Biologia Molecular Severo Ochoa, Departamento de Fisica de la Materia Condensada, Instituto Nicolas Cabrera, and Condensed Matter Physics Center (IFIMAC), Universidad Autonoma de Madrid, 28049 Madrid, Spain; 6 Proteomics Innovation Laboratory and Proteomics Core, Memorial Sloan Kettering Cancer Center, New York, NY 10065, USA; 7 Louis V. Gerstner Jr. Graduate School of Biomedical Sciences, New York, NY 10065, USA; 8 The SKI Stem Cell Research Facility, The Center for Stem Cell Biology and Developmental Biology Program, Sloan Kettering Institute, 1275 York Avenue, New York, NY 10065, USA; 9 Sean M. Healey and the AMG Center for ALS and the Neurological Clinical Research Institute, Massachusetts General Hospital, Harvard Medical School, Boston, MA, USA

**Keywords:** neuroinflammation, amyotrophic lateral sclerosis, C9orf72, motor neurons, astrocytes, microglia, triculture, 3D microtissue, drug screen, sartans

## Abstract

Amyotrophic lateral sclerosis (ALS) is a fatal neurodegenerative disease characterized by progressive motor neuron (MN) loss. The most common genetic cause, a hexanucleotide repeat expansion in *C9orf72* (C9-ALS), disrupts microglial function, contributing to neuroinflammation, a key disease driver. To investigate this, we developed a three-dimensional spinal microtissue (SM) model incorporating human induced pluripotent stem cell (hiPSC)-derived MNs, astrocytes, and microglia. Screening 190 Food and Drug Administration (FDA)-approved compounds, we identified sartans—angiotensin II receptor I blockers (ARBs)—as potent inhibitors of neuroinflammation. Telmisartan, a highly brain-penetrant ARB, significantly reduced the levels of pro-inflammatory cytokines interleukin (IL)-6 and IL-8 and rescued MN loss in C9-ALS SMs. Our findings suggest that C9-ALS microglia drive MN toxicity and that telmisartan can effectively mitigate inflammation and preserve MN viability. This work lays the groundwork for modeling disease-related neuroinflammation and points to telmisartan as a therapeutic candidate worth further exploration for treating C9-ALS.

## Highlights

• 3D spinal microtissue model (SM) to study neuroinflammation in C9ORF72 (C9)-ALS
• C9-ALS vs. isogenic control microglia and SMs show increased IL-6 and IL-8 levels
• Screen of FDA-approved drugs identifies sartans as inhibitors of neuroinflammation
• Telmisartan can preserve motoneuron survival against C9-microglia-mediated toxicity

## Graphical abstract

**Figure:**
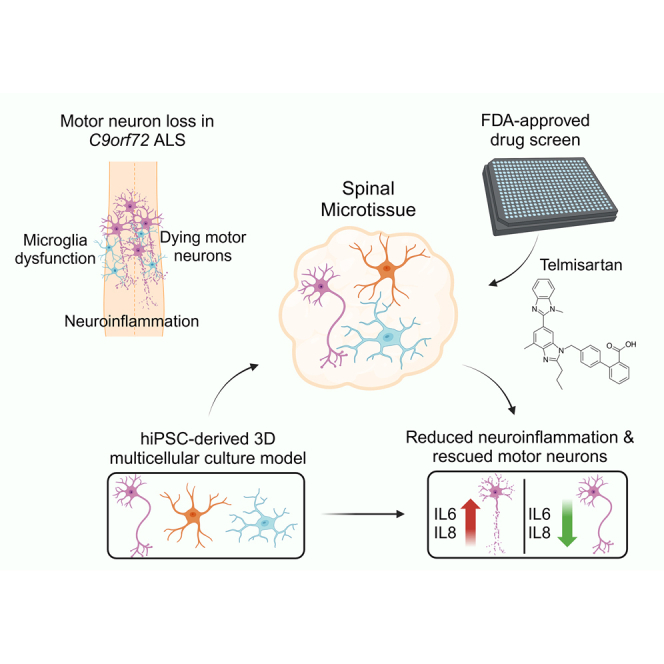

## Teaser

In this article, Sonustun, Giacomelli, Studer, and colleagues present a 3D spinal microtissue model of C9ORF72 (C9)-ALS to study disease-related neuroinflammation. An FDA-approved drug screen identifies sartans as potent inhibitors of C9-neuroinflammation. Telmisartan prevents motoneuron death mediated by C9-ALS microglia and may present a novel candidate therapeutic for C9-ALS.

## Introduction

Amyotrophic lateral sclerosis (ALS) is a fatal neurodegenerative disease characterized by motor neuron (MN) degeneration in the brain and spinal cord, leading to progressive paralysis and death within 3–5 years (Lall and Baloh, 2017). While most ALS cases lack an identifiable genetic cause, the expansion of the G_4_C_2_ hexanucleotide repeats (GGGGCC) within the first intron of the chromosome 9 open reading frame 72 (*C9orf72*) gene contributes to approximately 25%–40% of familial cases and 5% of sporadic ALS/FTD cases (Balendra and Isaacs, 2018; Boillée et al., 2006). The *C9orf72* gene is implicated in various physiological processes, particularly in microglial and myeloid cells, and in pathways related to immune regulation and glial cell functions (Balendra and Isaacs, 2018; Christoforidou et al., 2020; Masrori et al., 2022). Glial cell activation and dysfunction of astrocytes and microglia have been linked to both familial and sporadic ALS (Conlon et al., 2018) based on studies in postmortem tissue and in mouse and cellular models of the disease (Balendra and Isaacs, 2018; Boillée et al., 2006; Boillée et al., 2006; Christoforidou et al., 2020; Masrori et al., 2022; Saxena and Caroni, 2011). Neuroinflammation may be particularly important for driving ALS disease progression (Philips and Rothstein, 2014) and thereby presents a promising target for therapeutic intervention. However, there is a pressing need for a robust, scalable, and physiologically relevant model of human neuroinflammation to identify relevant candidate therapeutics.

Human pluripotent stem cells (hPSCs) offer a unique opportunity to capture neuro-glial interactions in relevant human cell types using patient-specific or genetically engineered stem cell lines carrying patient-related mutations. There is long-standing evidence that glial cells contribute to disease phenotypes as first described for SOD1 mutant ALS models (Di Giorgio et al., 2007; Nagai et al., 2007). *C9orf72* ALS (C9-ALS) models show phenotypes such as RNA foci or dipeptide repeat-related pathologies in neurons as reviewed recently (Giacomelli et al., 2022) including neurotoxic effects mediated by C9-microglia in co-culture platforms (Vahsen et al., 2022).

Here, we developed a scalable 3D spinal microtissue (SM) model integrating human induced pluripotent stem cell (hiPSC)-derived MNs, astrocytes, and microglia. C9-SMs showed elevated levels of secreted interleukin-6 (IL-6) and IL-8 compared to control (CTRL) SMs, reflecting a C9-ALS neuroinflammatory signature. Utilizing this inflammatory readout, we screened 190 Food and Drug Administration (FDA)-approved compounds; identified angiotensin II receptor I (AT1R) blockers (ARBs) as modulators of this neuroinflammatory signature; and demonstrated telmisartan’s ability to reduce inflammation and prevent MN loss.

## Results

### Development of a 3D SM triculture model

**Figure 1:**
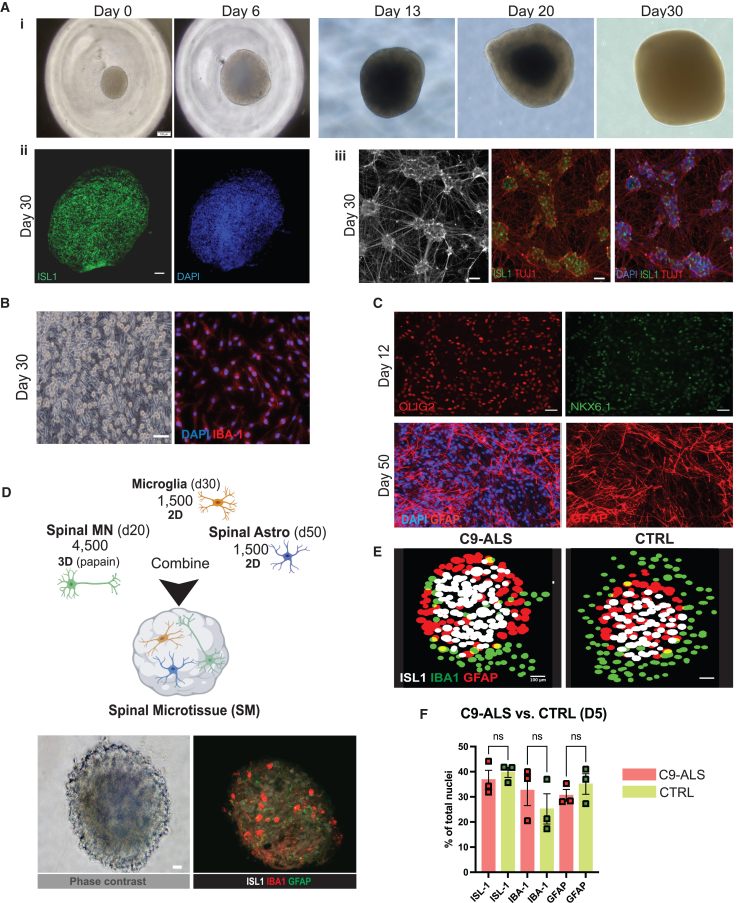
Characterization and workflow of hiPSC-derived spinal microtissues (A) (i) Representative bright-field images capture the 30-day development of 3D spinal motor neuron (MN) organoids, showing the transition from initial cell aggregation (day 0) to well-defined structures with increasing complexity at days 5, 10, 20, and 30. (ii) A cryosectioned hiPSC-derived spinal MN organoid at day 30 shows ISL-1 expression (green) with nuclear staining (DAPI, blue). (iii) Five days after papain dissociation, bright-field and immunofluorescence images display ISL-1 (green), TUJ1 (red), and DAPI (blue) at day 30. (B) Representative images of IBA-1-positive day 30 hiPSC-derived microglia in monolayer culture, including a corresponding bright-field image. (C) Representative images of hiPSC-derived neural stem cells (NSCs) on day 12 of differentiation, depicting NKX6.1 and OLIG2 staining (top panel). Bottom panel shows differentiated GFAP-positive spinal astrocytes on day 50. Scale bars represent 100 μm unless otherwise indicated. (D) A schematic illustrates the workflow for generating spinal microtissues (SMs) by combining spinal MNs (days 20–30), spinal astrocytes (day 50), and microglia (day 30). Cells were mixed in defined ratios, seeded into low-attachment 96-well V-bottom plates, centrifuged (10 min at 1,090 rpm), and incubated overnight at 37°C. Bright-field and immunofluorescence images at day 5 confirm SM integrity and composition. (E) Representative image outputs of SMs using the Object Segmentation, Counter and Analysis Resource (OSCAR) pipeline transformed into 2D representations depicting the three cell types as white (spinal MN), green (microglia), and red (spinal astrocytes). (F) Quantitative analysis of SMs reveals no significant differences in cell proportions between C9-ALS and CTRL on day 5 post-SM generation. The data presented in this figure were generated using confocal microscopy on whole-mount stained SMs. The quantifications are based on the z stack imaging, which captures the 3D architecture of the SMs as described in the methods section. Statistical analysis was performed using one-way ANOVA with Šídák’s multiple comparisons test. ns, not significant. ^∗^*p* < 0.05, ^∗∗^*p* < 0.01. Cumulative analysis from *N* = 3 differentiations. Data are shown as mean ± SEM.

To generate a 3D, multicellular SM model from hiPSCs, we first optimized two-dimensional (2D; monolayer) and 3D (organoid) protocols to direct differentiation into spinal motor neurons (spinal MNs), spinal astrocytes, and microglia. For these studies, we used patient C9-ALS hiPSCs and age- and gender-matched, healthy CTRL lines. Details on these cell lines are presented in Figure S1A. Spinal MNs were generated using a novel, highly efficient, and scalable 3D organoid protocol (Figure 1Ai) detailed in Figure S1B. This allowed us to generate high-purity spinal MNs (Figure S1C) between day 20–30 of differentiation (Figure 1Aii). Dissociated spinal MNs display typical neuronal morphology and express MN markers such as ISL-1 (Figure 1Aiii). For generating hiPSC-derived microglia, we used an adaptation of a previous protocol (Guttikonda et al., 2021) (Figures 1B and S1F). The resulting microglia are characterized by the presence of IBA-1 (Figure 1B) and CD14 (Figure S1G) on day 30. To obtain spinal cord astrocytes (spinal astrocytes), we directed the hiPSCs toward a spinal cord progenitor identity and transduced progenitors with nuclear factor I/A (NFIA), which yielded GFAP-positive astrocytes by day 50 (Figure 1C). Details of this protocol are summarized in Figure S1D. Day 50 spinal astrocytes showed characteristic morphology and expressed astrocyte makers such as GFAP, and their spinal identity was confirmed by the presence of Nkx6.1 and Olig2 by immunofluorescence (IF) (Figure 1C). All three cell types were generated from either C9-ALS or CTRL lines.

Next, spinal MNs, spinal astrocytes, and microglia from C9-ALS and CTRL hiPSCs were combined in suspension in 96-well V-bottom plates, as previously described for cardiac microtissues (Giacomelli et al., 2020) to generate a 3D, triculture platform, herein referred to as “spinal microtissue” (SM). Each SM is composed of 7,500 cells in total, consisting of 1,500 spinal astrocytes, 4,500 spinal MNs, and 1,500 microglia, ultimately forming a 1:3:1 ratio (Figure 1D) The cell composition of SMs was validated over time by assessing marker expression (ISL-1, IBA-1, and GFAP). No significant differences among cell types were found between C9-ALS and CTRL SMs on D5 (Figures 1E and 1F).

### Sartans decrease neuroinflammation in ALS SMs from C9orf72 hiPSC lines

**Figure 2:**
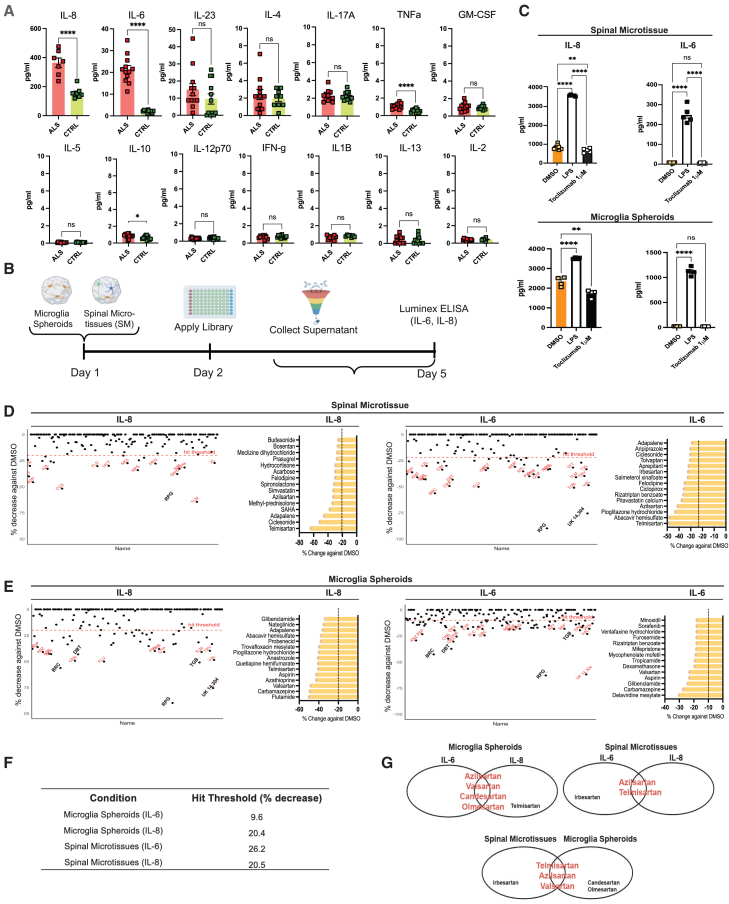
High-throughput screen of 190 FDA-approved compounds identifies angiotensin II receptor I blockers as modulators of neuroinflammation in C9-ALS (A) C9-ALS and CTRL spinal microtissues (SMs) were cultured for 5 days, and supernatants were collected on day 5. IL-6 and IL-8 were identified as C9-ALS-associated inflammatory signatures by using multiplex cytokine profiling from the supernatants. *N* = 2 independent differentiations, two C9-ALS patient lines, 3 technical replicates per differentiation. Data are shown cumulatively as mean ± SEM. (B) Experimental design highlighting the SM and microglia spheroid generation, treatment paradigm, and experiment timeline. HTS was performed on C9-ALS spinal microtissues, as well as microglia spheroids. After generating the microtissues/spheroids on D1, the TocriScreen library was applied at 5 μM and plates were incubated at 37^o^C for 72 h. Subsequently, the supernatants were collected from each well and sent for multiplex cytokine profiling, specific for IL-6 and IL-8. (C) IL-6 and IL-8 concentrations in C9-ALS SMs and microglia spheroids treated with negative control (tocilizumab, 1 μM), positive control (LPS, 1 μg/mL), and vehicle control (DMSO, 0.01%) demonstrate significantly lower IL-6 and IL-8 concentrations in vehicle control-treated groups relative to LPS-treated groups in both the SM and microglia spheroid groups. The percent decrease in IL-6 and IL-8 in tocilizumab-treated groups against vehicle control-treated groups was used as a cutoff threshold for determining hits (referred to as “hit threshold”). These thresholds are detailed in Figure 2F. (D) Screen results, shown as percent decrease against the vehicle control, highlight compounds that lowered IL-8 and IL-6 in C9-ALS SM supernatants. The red dashed line indicates the hit threshold for each condition. Red labels denote top hits, identified by their abbreviations (full names in Figure S2A), while black labels indicate toxic compounds removed from further analysis. Bar charts rank the top 15 hits by potency of inhibition, defined as the percent reduction in IL-6 or IL-8 relative to the vehicle control. Data were generated from a single differentiation (technical replicate). (E) Screen results show the percent decrease in IL-8 and IL-6 concentrations relative to vehicle control, highlighting hits in C9-ALS microglia spheroid supernatants. This panel specifically represents findings for microglia spheroids. See (D) for details on thresholds, labels, and ranking criteria. (F) Hit thresholds are depicted as % decrease of IL-6 or IL-8 with 1 μM tocilizumab treatment against DMSO in either SMs or microglia spheroids. (G) Venn diagrams depicting shared ARB hits among each group and those that were mutually exclusive. Telmisartan, Valsartan, and Azilsartan are identified as the hits that are shared between spinal microtissues and microglia spheroids.

Cytokine profiling in C9-ALS and CTRL SM supernatants was performed to detect supernatant concentrations and fluorescence intensity of 14 inflammatory cytokines (Figure 2A). We selected IL-6 and IL-8 as inflammatory readouts for our compound screen, as they showed the best signal to noise ratio. Next, we performed a chemical screen of 190 FDA-approved compounds to identify modulators of neuroinflammation exclusively in C9-ALS SMs, using the TocriScreen FDA-Approved Drugs Library (Figure S2A). Because our pilot experiments strongly suggested a key role for microglia in driving IL-6 and IL-8 secretion in C9-ALS, the screen was performed in both C9-ALS SMs and in C9-ALS microglia similarly aggregated in 3D (microglia spheroids containing 7,500 cells/spheroid). At 24 h after microtissue generation (day 2), the chemical library was applied at 5 μM final concentration for each compound, and supernatants were collected 72 h later (day 5) (Figure 2B). The screen was performed in ultra-low-attachment 96-well V-bottom plates in single wells for the compounds and triplicates for the positive, negative, and vehicle CTRLs. IL-6 and IL-8 secretion were used as a readout for C9-ALS neuroinflammatory signature. Lipopolysaccharid (LPS; 1 μg/mL) was used as the positive CTRL needed to induce the opposite effect—namely, increasing inflammation. Tocilizumab (1 μM), a soluble IL-6 receptor (sIL6R) blocking antibody, was used as negative CTRL. By blocking trans-IL-6 signaling, we anticipated that it would reduce both IL-6 and IL-8 levels. 0.01% DMSO was used as vehicle CTRL. Tocilizumab showed little to no effect compared to DMSO in the case of IL-6 levels suggesting that blocking IL-6 signaling does not dramatically impact its secretion (Figure 2C). In addition, we assessed microtissue viability by measuring the diameter of each microtissue compared to the untreated wells and the DMSO-treated groups. We observed no significant decrease in SM viability for most compounds, including CTRLs. Compounds affecting microtissue or microglia spheroid viability were excluded from further analysis (Figure S2B). We quantified secreted IL-6 and IL-8 percent (%) reductions relative to DMSO-treated CTRLs as the primary screening readout. Hits were defined as compounds reducing IL-6 and/or IL-8 beyond set thresholds.

In C9-ALS SMs, we identified 24 IL-8 hits (20%–70% decrease) and 23 IL-6 hits (20%–60% decrease) (Figure 2D). In C9-ALS microglia spheroids, 45 compounds reduced IL-8 (20%–90%) and 66 reduced IL-6 (15%–60%) (Figure 2E). The top 15 hits per group were selected based on potency of IL-6 and IL-8 inhibition (Figures 2D and 2E). In SMs, telmisartan ranked the highest, reducing IL-8 by 65% and IL-6 by 50% (Figure 2D). In microglia spheroids, flutamide and delavirdine mesylate were the top IL-8 and IL-6 inhibitors, respectively (Figure 2E). Hit thresholds were determined using the negative CTRL (tocilizumab-treated) versus DMSO (Figure 2F). Notably, sartans, the top hits in C9-ALS SMs, were also effective in microglia spheroids, suggesting their ability to reduce IL-6 and IL-8 in both platforms (Figure 2G). Further details are in the methods section. To assess screen robustness, we performed a Z-prime factor analysis, measuring signal separation between the positive and vehicle CTRLs. Results were plotted as probability distributions against log-transformed assay signals, visualized as bell curves (Figures S2C and S2D).

### Confirmation of telmisartan as a key hit in decreasing neuroinflammation in C9-ALS SMs

**Figure 3:**
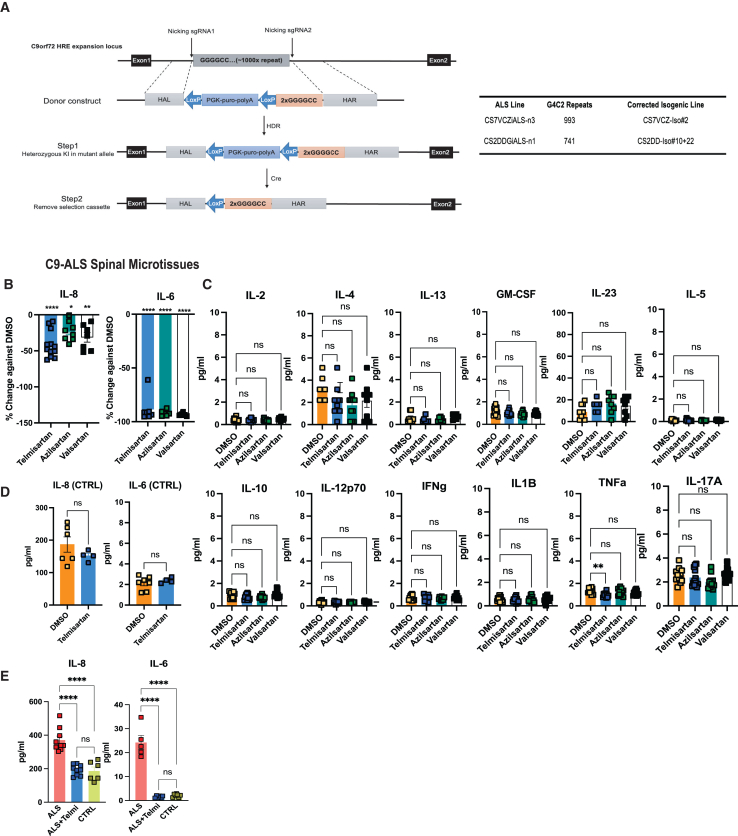
Validation of ARBs on neuroinflammation in C9-ALS spinal microtissues (A) Gene correction of C9orf72 ALS patient iPSC lines. The CS7VCZiALS and CS2DDGiALS iPSC lines, carrying heterozygous G4C2 repeats of 993 and 741 sequences, respectively, were corrected using CRISPR-Cas9-mediated HDR. (B) Validation screen was performed in C9-ALS SMs, which were treated with Telmisartan, Valsartan, and Azilsartan (all compounds at 5 μM) following the same paradigm as the HTS. Cytokine analysis shows the percent decrease in IL-6 and IL-8 concentrations relative to DMSO (vehicle control). One-way ANOVA with Šídák’s multiple comparisons test ^∗^*p* < 0.05, ^∗∗^*p* < 0.01, ^∗∗∗∗^*p* < 0.001. *N* = 3 replicates, 3 independent differentiations from 2 different lines for each. Data are represented as mean ± SEM. (C) Bar graphs depict the effects of telmisartan, azilsartan, and valsartan on IL-2, IL-4, IL-5, IL-12, IL-13, GM-CSF, IL-10, IL-12p70, interferon (IFN)γ, IL-1β, TNF-α, and IL-17a concentrations. Results show no significant differences in the cytokine concentrations in the C9-ALS SM supernatants after 72 h of treatment, except for a significant decrease in TNF-α with 5 μM telmisartan treatment. One-way ANOVA with Šídák’s multiple comparisons test, ^∗^*p* < 0.05, ^∗∗^*p* < 0.01, ^∗∗∗∗^*p* < 0.001. *N* = 3 replicates, 3 independent differentiations from 2 different lines. Data are represented as mean ± SEM. (D) Telmisartan was applied to healthy control SMs at 5 μM following the same paradigm. Bar graphs show no significant difference in IL-6 and IL-8 concentrations between DMSO and telmisartan treatment. Student’s t test with multiple comparisons. ns, not significant. *N* = 3 replicates, 2 independent differentiations from two isogenic control lines. Data are represented as mean ± SEM. (E) Bar graphs depicting IL-6 and IL-8 concentrations from C9-ALS, control, and C9-ALS SMs treated with 5 μM telmisartan. Results show that telmisartan treatment lowers IL-8 and IL-6 concentrations in C9-ALS SMs to levels observed in control SMs. One-way ANOVA with Šídák’s multiple comparisons test, ^∗^*p* < 0.05, ^∗∗^*p* < 0.01, ^∗∗∗∗^*p* < 0.001. *N* = 3 replicates, 3 independent differentiations from 4 different lines for all the panels. Data are represented as mean ± SEM.

Because sartans were the top hits reducing IL-6 and IL-8 in both microglia and C9-ALS SMs, we validated their effects using telmisartan, valsartan, and azilsartan in a secondary screen. Validation was conducted on C9-ALS SMs derived from different hiPSC lines and their isogenic CTRLs, generated via CRISPR-Cas9 homology-directed repair (HDR). Details, including the gene editing strategy using CRISPR-Cas9 (HDR) to generate these lines, are depicted in Figure 3A. Following the original screening design, SMs were treated with 5 μM sartans on D2 and cultured for 72 h before supernatant analysis. Telmisartan, azilsartan, and valsartan significantly reduced IL-6 and IL-8 in C9-ALS SMs (Figures 3B and 3C). However, telmisartan had no significant effect on IL-6, IL-8, or other cytokines in isogenic CTRL SMs (Figure 3D), suggesting its effect is specific to C9-ALS (Figure S3A). Secretome analysis confirmed IL-8 (CXCL8) as a top upregulated protein in C9-ALS SMs (Figure S3B) and the most significantly reduced following telmisartan treatment, further supporting our findings (Figure S3C).

### Telmisartan enhances survival of spinal MNs in C9-ALS co-cultures and tricultures

**Figure 4:**
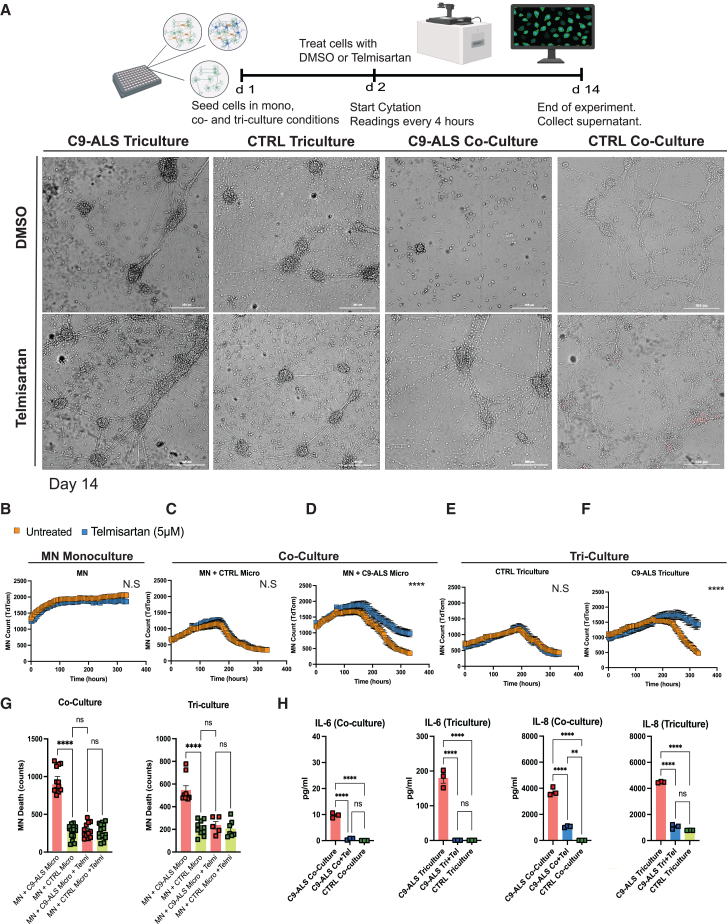
Telmisartan enhances motor neuron survival in C9-ALS tri- and co-culture models (A) Experimental timeline and setup for assessing the effects of DMSO and telmisartan on cells cultured under 2D mono-, co-, and tri-culture conditions. All MN were wild-type and tagged (GPI:H2B-TdTom). On day 0 (d0), cells were seeded into a 96-well plate in the specified culture conditions. On day 1 (d1), cells were treated with either DMSO or telmisartan. Starting from day 2 (d2), live-cell imaging was initiated using a Cytation instrument, with readings taken every 4 h over a 14-day period to monitor cellular responses. Bottom panel depicts bright-field images at 336 h. Td-tomato counts were recorded every 4 h. (B) The effects of telmisartan (5 μM) on motor neuron (MN) counts over time were assessed across various experimental conditions, comparing untreated (DMSO, orange) and telmisartan-treated (blue) groups. Td-Tomato counts were recorded every 4 h for 336 h in the following settings: MN alone, MN + CTRL microglia, MN + C9-ALS microglia, C9-ALS triculture (MN + C9-ALS microglia + C9-ALS astrocytes), and CTRL triculture (MN + CTRL microglia + CTRL astrocytes). CTRL refers to cells derived from either (1) healthy control donors age- and gender-matched to C9-ALS patient lines or (2) isogenic controls generated by correcting the C9orf72 repeat expansion in C9-ALS patient lines. Scatterplots illustrate differences in Td-Tomato counts over 14 days between telmisartan-treated and untreated groups across these conditions. (C) When MNs are co-cultured with control microglia, there is also no significant difference (NS) in MN counts between untreated and telmisartan-treated groups. (D) MNs co-cultured with C9-ALS microglia, telmisartan treatment results in higher MN counts compared to untreated groups, suggesting a protective effect of telmisartan on MNs in the presence of C9-ALS microglia. (E) In the control triculture model, telmisartan treatment does not significantly affect MN survival, as indicated by the no significant difference (NS) in MN counts over time between untreated and telmisartan-treated groups. (F) Telmisartan treatment in the C9-ALS triculture group results in significantly higher motor neuron (MN) counts compared to the untreated group. (G) MNs co-cultured with C9-ALS microglia exhibit significantly higher death counts compared to MNs co-cultured with CTRL microglia (*p* < 0.001). Treatment with telmisartan significantly reduces MN death in the C9-ALS co-culture to levels similar to those seen in MNs co-cultured with CTRL microglia. There is no significant difference (NS) in MN death counts between untreated and telmisartan-treated CTRL microglia co-cultures. Similarly, MNs in the presence of C9-ALS microglia+C9-ALS astrocytes show significantly higher death counts compared to MNs with CTRL microglia (*p* < 0.001). Telmisartan treatment again significantly reduces MN death in the C9-ALS triculture to levels comparable to those in the CTRL triculture, with no significant difference (NS) observed between untreated and telmisartan-treated CTRL tricultures. (H) Supernatants were collected on day 14 and sent for multiplex cytokine analysis. Results demonstrate that, in C9-ALS co-cultures and tricultures, IL-6 and IL-8 levels are significantly elevated compared to CTRLs (^∗∗∗∗^*p* < 0.001). Telmisartan treatment significantly reduces IL-6 and IL-8 levels in both C9-ALS co-cultures and tricultures (^∗∗∗∗^*p* < 0.001), reducing the levels to baseline CTRL levels. One-way ANOVA with Šídák’s multiple comparisons test, ^∗^*p* < 0.05, ^∗∗^*p* < 0.01, ^∗∗∗∗^*p* < 0.001. Data were generated using two C9-ALS patient lines and their isogenic controls. *N* = 3 replicates each. Data are represented as mean ± SEM.

To assess telmisartan’s effect on spinal MN survival under C9-ALS conditions, we generated spinal MNs from a GPI:H2B-tdTomato-tagged hESC line (Guttikonda et al., 2021) and cultured them alone or with C9-ALS or CTRL glia (microglia and/or spinal astrocytes) under mono-, co-, and triculture conditions. Cells were treated with DMSO (CTRL) or telmisartan (5 μM) on D2, and live-cell imaging was performed every 4 h for 14 days (Figure 4A). Td-Tomato counts showed no significant difference in MN survival between untreated and telmisartan-treated groups in monoculture (Figure 4B) or when co-cultured with CTRL microglia (Figure 4C). However, telmisartan significantly improved MN survival in co-culture with C9-ALS microglia (Figure 4D). In the triculture model, telmisartan had no effect with wild-type glia (Figure 4E) but significantly increased MN survival in the C9-ALS glia-based setting (Figure 4F). Analysis of “motor neuron death” (net Td-Tomato change between the first and last time points) showed that MNs co-cultured with C9-ALS microglia exhibited significantly higher death counts compared to those co-cultured with CTRL microglia, and telmisartan treatment reduced MN death in the C9-ALS co-culture to levels similar to CTRL microglia (Figure 4G). No significant difference was observed between untreated and telmisartan-treated CTRL microglia co-cultures. Similarly, MNs in C9-ALS tricultures, which included both microglia and astrocytes, showed significantly higher death counts compared to those co-cultured with CTRL microglia (Figure 4G). Finally, supernatant collected after 14 days of culture was analyzed for cytokine levels, revealing that IL-6 and IL-8 levels were significantly elevated in C9-ALS co-cultures and tricultures compared to CTRLs (Figure 4H). Telmisartan treatment significantly reduced IL-6 and IL-8 levels in both C9-ALS co-cultures and tricultures, restoring them to baseline CTRL levels (Figure 4H). These findings suggest that telmisartan has a neuroprotective effect in C9-ALS models derived from patient induced pluripotent stem cells (iPSCs) by enhancing MN survival in a non-cell-autonomous manner and reducing C9-microglia-mediated pro-inflammatory cytokine levels.

### Telmisartan shows time-dependent effect on C9-ALS SM cell ratios and rescues MN proportion

As the previous survival studies were performed in 2D co-culture and tri-culture models (Figure 4), we explored whether we could assess the role of telmisartan in our 3D SM platform using whole-mount staining to quantify cell proportions over a 14-day period. SMs were generated and treated with either 0.01% DMSO or 5 μM telmisartan using the same experimental paradigm depicted in Figure 2B. SMs were collected and fixed on days 5 (D5), 7 (D7), and 14 (D14) post-generation and stained using whole-mount IF analysis, as described in the methods section. Antibodies against ISL-1 (spinal MNs), IBA-1 (microglia), and glial fibrillary acid protein (GFAP; spinal astrocytes) were used to determine the proportions of the three cell types over time in culture. The bar graphs (Figures S4A–S4D) depict the proportions of IBA-1+ microglia, GFAP+ astrocytes, and ISL-1+ MNs on D5, D7, and D14. Interestingly, the percentage of ISL-1+ MNs was significantly lower in C9-ALS compared to CTRL SMs on D14 (Figure S4F). With telmisartan treatment (C9-ALS + Telmi), ISL-1+ MN proportions on D5 and D7 showed a slight increase compared to their DMSO-treated counterparts (Figures S4A and S4C), with the most significant difference observed on D14, where the ISL-1+ MN population was significantly higher with telmisartan treatment compared to the DMSO group on D14 (Figures S4C and S4E). In the CTRL (DMSO) group, ISL-1-positive cells remained similar across all the time points, regardless of telmisartan treatment (Figures S4B and S4D). Collectively, these results support our findings that MN populations decline over time in C9-ALS compared to CTRL SMs and that telmisartan can mitigate this effect.

## Discussion

Our SM model provides a controlled *in vitro* platform to study cell-autonomous and non-cell-autonomous interactions while enabling scalable high-throughput screening with just 7,500 cells per microtissue. Its novelty lies in mimicking the dynamic interplay between MNs and glia at physiological cell ratios under highly defined conditions, offering a more accurate representation of disease states. By deriving all cell types from a single hiPSC source, it ensures a physiologically relevant cellular environment, preserving intricate cellular interactions often missed in simpler models. Our findings, particularly the identification of sartans as IL-6 and IL-8 modulators with neuroprotective potential, highlight the model’s value in drug discovery. IL-6 and IL-8 have emerged as neuroinflammatory signatures in the context of ALS, with some studies highlighting their elevated levels in human ALS cerebrospinal fluid (CSF) and plasma (Mennini et al., 2009; Moreau et al., 2005; Wosiski-Kuhn et al., 2021). These cytokines are thought to play roles in mediating the inflammatory response within the CNS, contributing to the progression of neurodegeneration (Zhang et al., 2023). IL-6 is known to promote the survival and differentiation of neurons under normal conditions, but, in the diseased state, its chronic elevation may lead to detrimental effects, including the exacerbation of neuroinflammation and neuronal loss (Ehrhart et al., 2015; Tortelli et al., 2020). Similarly, IL-8 has been implicated in the inflammatory milieu associated with ALS (Ehrhart et al., 2015; Femiano et al., 2024; Masrori et al., 2022). In our SM model, we identified these cytokines as neuroinflammatory signatures specific to C9-ALS not observed in CTRL SMs, highlighting their potential role in the disease pathology. By using our model, we screened 190 compounds to identify modulators of IL-6 and IL-8 and identified multiple sartans as hits. We also demonstrated a correlation between neuroprotection and the modulation of IL-6 and IL-8 in C9-ALS.

In contrast to the elevated IL-6 and IL-8 levels in C9-ALS SMs and microglia spheroids, we found low levels in C9-ALS astrocytes and MNs, highlighting microglia as key mediators of the disease-related neuroinflammatory response. Beyond secreting IL-6 and IL-8, C9-ALS microglia were neurotoxic, even in the absence of external stimuli, unlike previous studies using LPS or excitotoxic challenges (Banerjee et al., 2023; Vahsen et al., 2022, 2023). This suggests that C9-ALS is not solely MN autonomous but actively driven by microglia, potentially via IL-6 and IL-8 pathways. Our SM model confirmed this role, showing significant MN loss in the C9-ALS group, which was rescued by telmisartan treatment. These findings enable further mechanistic studies on neuroinflammation and the identification of key drivers of MN toxicity in C9-ALS microglia.

Our data implicate the renin-angiotensin-aldosterone system (RAAS) in C9-ALS pathogenesis. RAAS, primarily known for regulating blood pressure (Nakagawa et al., 2020), is also active in the CNS, where angiotensin II (Ang II) and its receptors contribute to neuroinflammation, oxidative stress, and blood-brain barrier disruption (Huang and Zhang, 2024). AT1R activation exacerbates microglial activation and pro-inflammatory cytokine release (Kim et al., 2022), while RAAS inhibition via ARBs or angiotensin converting enzyme (ACE) inhibitors may reduce neuroinflammation (Benicky et al., 2011; Joglar et al., 2009). Sartans, prescribed for hypertension and heart failure, are not currently used for inflammatory conditions, though some studies suggest anti-inflammatory effects in cardiovascular disease and diabetes (Chen et al., 2018; Klinghammer et al., 2013). A meta-analysis also reported that telmisartan reduces IL-6 and tumor necrosis factor alpha (TNF-α), supporting its potential as an anti-inflammatory agent (Takagi et al., 2013). Beyond systemic RAAS, brain-specific RAAS (b-RAS) has been implicated in local immune modulation (Sigmund and Grobe, 2020) and microglial polarization via NADPH oxidase activation (Labandeira-Garcia et al., 2017). AT1R inhibition has shown functional benefits in preclinical models of stroke (Wanderer et al., 2020), Alzheimer’s (Mogi and Horiuchi, 2009), and Parkinson’s disease (Labandeira et al., 2022). Telmisartan (Micardis) is a potent, blood-brain barrier (BBB)-penetrant ARB (Noda et al., 2012), with cohort studies suggesting an inverse correlation between RAS-acting drugs and neurodegenerative diseases, including Alzheimer’s disease (Ho et al., 2021; Lee et al., 2023). Notably, ACE inhibitors have been linked to a lower ALS incidence (Lin et al., 2015), but no studies have explored ARBs in C9-ALS. Here, we demonstrate sartans, particularly telmisartan, as potential C9-ALS therapeutics by reducing neuroinflammation and MN death.

Our findings suggest that IL-6 and IL-8 are key neuroinflammatory cytokines in C9-ALS and that microglia play an active role in its pathology. These results support targeting IL-6 and IL-8 pathways for therapy, including testing telmisartan in C9-ALS progression. However, IL-6 or IL-8 alone did not replicate MN death observed with C9-ALS microglia or tricultures, suggesting that additional toxic factors contribute. Telmisartan may offer broader benefits beyond direct IL-6/IL-8 inhibition. For translational studies, determining whether peripheral telmisartan administration can achieve therapeutic CNS levels comparable to our 5 μM *in vitro* concentration is critical. Its high plasma protein binding (∼99.5%) limits free drug availability, but preclinical studies in monkeys (1 mg/kg IV) show BBB penetration and AT1R inhibition in the brain (Noda et al., 2012). Translating this to humans may require intrathecal delivery for optimal neuroprotection. The SM platform, leveraging hiPSC technology, integrates the spinal cord neuroinflammatory axis while maintaining scalability for high-throughput screening. It is cost effective, easy to fabricate, and adaptable for drug discovery. Beyond ALS, SMs could advance personalized medicine by predicting drug efficacy, optimizing clinical trial design, and guiding patient stratification. Realizing the full potential of this platform could accelerate ALS research and translate promising findings from bench to bedside.

### Limitations of the study

Our study demonstrates a robust effect of multiple sartans on neuroinflammation in *C9orf72* SMs and in microglia and shows that telmisartan protects spinal motoneurons from *C9orf72-*-microglia-mediated death *in vitro*. However, the study has several limitations. First, although our screening was conducted in 3D microtissues, the pathophysiological analysis in 3D microtissues remains limited. Second, while we utilized hiPSCs from two independent *C9orf72* backgrounds, two age-matched CTRLs, and two gene-corrected isogenic clones corrected to physiological repeat length, future studies are needed to test larger panels of *C9orf72* and sporadic hiPSC lines. Such studies may address whether our findings are broadly applicable to ALS or specific to *C9orf72.* Third, our focus was on the impact of the *C9orf72* mutation in triculture models and microglia-MN co-cultures. However, we have not explored a specific role of *C9orf72* astrocytes using co-cultures of astrocytes and MNs, and the specific factors driving the increase in inflammatory cytokines in C9orf72 microglia remain unclear. Furthermore, studies are needed to address whether the anti-inflammatory and neuroprotective effects are directly mediated by the Ang II type 1 receptor. Finally, this manuscript focused on validating sartans, and other candidate hits from our screen need further validation but may represent interesting targets for future research.

## Methods

### hiPSC cell culture

Experiments using human pluripotent stem cells were reviewed by the Tri-institutional ESCRO committee. Human iPSCs were maintained in Essential 8 medium on Vitronectin-coated plates, as previously described (Tchieu et al., 2017) The working cell banks (WCBs) were expanded from the master cell banks obtained from the Answer ALS repository. hiPSC stocks were passaged at a 1:6 ratio, cryopreserved using Stem Cell Banker as the freezing medium, and subsequently thawed into Essential 8 medium supplemented with 10 μM ROCK inhibitor (Y-27632). Cells were maintained in this medium for 24 h post-thawing. All WCB stocks were used between passages 20–30, and differentiations were initiated two passages following thawing. For additional information see supplemental methods.

### Generation and dissociation of 3D, spinal MN organoids from hiPSCs in 3D

Spinal MNs were generated in 3D as organoids. For dissociation, 10 organoids were collected and subjected to a papain dissociation technique following manufacturer instructions. For additional information on MN generation and dissociation see supplemental methods.

### Generation of spinal astrocytes and microglia from hiPSCs

The spinal astrocyte protocol was adjusted from Tchieu et al. (2019) to generate spinal cord-specific astrocytes. Microglial differentiation followed Guttikonda et al. (2021). For additional information on those two protocols see supplemental methods.

### Generation of SMs

SMs were generated similarly to cardiac microtissues (Giacomelli et al., 2020) but by mixing microglia, spinal MNs, and spinal astrocytes at a ratio of 1:3:1 in SM media—Neurobasal media containing brain-derived neurotrophic factor (BDNF; 0.02 μg/mL), glial cell line-derived neurotrophic factor (GDNF; 0.02 μg/mL), and IL-34 (100 ng/mL). Briefly, microglia and astrocyte stocks were thawed and allowed to recover in serum-free medium for one week, whereas day 20–30 spinal MN organoids were dissociated using papain dissociation (Worthington Biochemical Corporation, Cat #: LK003150) for 1 h and 10 min on an orbital shaker and counted by trypan blue. Meanwhile, day 30 microglia were dissociated into a single-cell suspension using Accutase for 10 min at 37^o^ C and counted by trypan blue. Simultaneously, day 50 spinal astrocytes were dissociated with trypsin at 37^o^ C, and cells were counted by trypan blue. Spinal MNs, microglia, and spinal astrocytes were mixed to generate a total of 7,500 cells comprising 4,500 spinal MNs, 1,500 spinal astrocytes, and 1,500 microglia per microtissue and seeded in 100 μL of microtissue media per well in a low-attachment 96-well V-bottom plate (S-Bio PrimeSurface 3D culture: ultra-low-attachment plates, Cat #MS-9096VZ). Plates were centrifuged at 1,090 rpm for 10 min at room temperature (RT) and incubated under standard cell culture conditions (37^o^C, 5% CO_2_).

### High-throughput screen

SM and microglia spheroids were generated in 100 μL of media as described earlier. After 24 h, the wells were treated with the TocriScreen FDA-Approved Drugs Library (Tocris, Cat. #7200) using a semi-automated system. The screening was conducted in single wells for the compounds, with triplicates for the positive, negative, and vehicle CTRLs. The final concentration of each compound was 5 μM. Plates were incubated at 37°C with 5% CO_2_ for 72 h. After incubation, supernatants were carefully collected without disturbing the cells using a robotic system. The supernatants were immediately frozen at −80°C for subsequent analysis. Cytokine levels (IL-6 and IL-8) were measured using Eve Technologies’ Human High Sensitivity Custom 2-Plex Cytokine Assay. Quantitative analysis was performed in collaboration with the MSKCC Gene Editing & Screening Core.

### Whole-mount staining of microtissues

To stain SMs in 3D, we modified protocols described previously (Dekkers et al., 2019; Giacomelli et al., 2020). For additional information see supplemental methods.

### Immunostaining of monolayer cultures

Cells were fixed in 4% paraformaldehyde in PBS for 20 min, permeabilized in 0.1% Triton X-100 in PBS for 15, and blocked for 30 min in PBS with 1% bovine serum albumin (BSA). Primary antibody incubation was performed overnight at 4 °C at the specified dilutions in 1% BSA-PBS. Following three washes with PBS, cells were incubated with fluorescently conjugated secondary antibodies (2 μg mL^−1^) and DAPI (1 μg μL^−1^) for 45 min at RT. Antibody details can be found in the supplemental information.

### Cytokine arrays

This study used Luminex xMAP technology for multiplexed quantification of 14 human cytokines, chemokines, and growth factors. The multiplexing analysis was performed using the Luminex 200 system (Luminex, Austin, TX, USA) by Eve Technologies (Calgary, Alberta). For additional information see supplemental methods.

### Protein digestion

Samples were loaded into Amicon Ultra centrifugal filters (3 kDa molecular weight cut off), concentrated to a final volume of 100 μL, and transferred to a new Eppendorf tube. The filters were then rinsed with 4 M urea and 50 mM EPPS pH 8.5, and the rinse was transferred to the samples tube. After protein quantification using the Pierce bicinchoninic acid assay (Thermo Fisher Scientific), the proteins were reduced with tris (2-carboxyethyl) phosphine to a final concentration of 5 mM for 20 min at RT. Free cysteine residues were alkylated with iodoacetamide at a final concentration of 10 mM for 20 min in the dark at RT. The samples were cleaned using SP3 Sera-Mag carboxylate SpeedBeads beads (Cytiva, cat#: 65152105050250; 45152105050250, 50 μg/μL) according to the manufactures’ instructions. The beads were then resuspended in 50 μL of 50 mM triethylammonium bicarbonate, and the samples were digested with Lys-C (Wako) at a 1:100 enzyme-to-protein ratio and trypsin (Promega) at a 1:100 enzyme-to-protein ratio at 37°C for overnight incubation with shaking (1200 rpm) on a thermomixer (Thermo Fisher Scientific). After digestion, the samples were acidified with 2.65 μL of 99% formic acid (FA) at RT for 5 min with shaking (1,200 rpm), followed by addition of 1.2 mL of 100% acetonitrile (ACN) for 10 min at RT with shaking (1,200 rpm). The samples were washed three times with 100% ACN and resuspended in 50 μL of 2% DMSO at 37°C for 30 min with shaking (1,200 rpm). The eluted peptides were dried under vacuum, reconstituted in water with 0.1% FA, and sonicated in a water bath sonicator. Peptide yield was quantified using a NanoDrop (Thermo Fisher Scientific).

### Mass spectrometry analyses

Peptides were separated on a 25 cm column with a 75 μm diameter and 1.7 μm particle size, composed of C18 stationary phase (IonOpticks Aurora 3 1801220). The separation was carried out using a 25-min gradient: from 3.2% to 9.6% B over 2 min at a flow rate of 400 nL/min and then to 18% B over 12 min at 200 nL/min, to 32% B over 19.5 min at 200 nL/min, and finally to 90% B over 5 min at 300 nL/min. The mobile phase A was 0.1% FA in high-performance liquid chromatography-grade water, and mobile phase B ACN with 0.1% FA. The separation was performed using a Vanquish Neo system (Thermo Fisher Scientific). Mass spectrometry (MS) data were acquired on an Orbitrap Astral mass spectrometer (Thermo Fisher Scientific) in a data-independent acquisition mode, with a normalized collision energy of 25%. MS1 spectra were acquired in the Orbitrap at a resolution of 240 K, with a normalized automated gain control target 200%, a custom maximum injection time, and a scan range of 380–980 m/z. MS/MS spectra were acquired in the Astral analyzer with a 2 m/z isolation window, a scan range of 150–2,000 m/z, a precursor mass range of 380–980 m/z, and a loop CTRL time of 0.6 s.

### Analyses of MS data

Raw data files were processed using Spectronaut version 18.5 (Biognosys) and searched with the PULSAR search engine against a *Homo sapiens* UniProt protein database downloaded on 2024/05/28 (226,232 entries). Cysteine carbamidomethylation was specified as fixed modifications, while methionine oxidation, acetylation of the protein N-terminus, and deamidation (NQ) were set as variable modification. A maximum of two trypsin missed cleavages were allowed. Searches utilized a reversed sequence decoy strategy to CTRL peptide false discovery rate (FDR), with a 1% FDR threshold set for identification. An unpaired t test was used to calculate *p* values in differential analysis. The volcano plot was generated based on log2 fold change (log2FC) and *q* value (multiple testing corrected *p* value using the Benjamini-Hochberg method). A *q* value of ≤0.05 was considered the statistically significant cutoff.

### Live imaging with BioTek Cytation 5 cell imaging multimode reader

We utilized the BioTek Cytation 5 cell imaging multimode reader, which integrates automated microscopy and multimode detection. For additional information see supplemental methods.

### Flow cytometry

Flow cytometry analysis of CD14 expression was performed using a PE-conjugated anti-CD14 antibody (clone M5E2, STEMCELL Technologies), after incubation with FcR blocking reagent (Miltenyi Biotec). ISL-1 and IBA-1 expression were detected following fixation and permeabilization of cells using BD Cytofix/Cytoperm solution (BD Pharmingen). Cells were marked with Zombie Violet Viability (BioLegend). After incubation with FcR blocking reagent (Miltenyi Biotec), cells were stained with anti-IBA (clone 019-19741, Wako) and anti-ISL-1 (Clone 39.4D5-s, Developmental Studies Hybridoma Bank) primary antibodies, followed by staining with Alexa Fluor 488- and Alexa Fluor 555-conjugated secondary antibodies (Thermo Fisher Scientific). Flow cytometry was performed using a BD Biosciences LSR Fortessa flow cytometer with Diva software. Data were analyzed using FlowJo (BD Biosciences LLC).

### Gene correction and validation for C9orf72 ALS iPSC line

The C9-ALS patient hiPSC lines were obtained from Answer ALS (Baxi et al., 2022): the CS7VCZiALS iPSC lines carries a heterozygous 993 G4C2 repeating sequences, and the CS2DDGiALS carries a heterozygous 741 G4C2 repeating sequences in the intron 1 of *C9orf72* gene. The gene correction was performed using CRISPR-Cas9-based HDR. For additional information see supplemental methods.

### 3D image processing and analysis

Image processing and analysis were conducted using Fiji in conjunction with custom computational routines implemented in Python and Julia. A standardized pipeline was applied to each confocal image, encompassing kernel size determination, image processing, and 3D object classification and analysis as described in the supplemental methods.

### Statistical analysis

Results are shown as mean ± SEM. Mean values were compared between CTRL cells and cells from the patients in one-way ANOVA with Sidak’s multiple comparisons tests. One-way ANOVA, two-way ANOVA, Student’s t test, for paired or unpaired measurements was applied where indicated and appropriate. Statistical analysis was performed using GraphPad Prism 9 (v.9.2.0). Statistical significance is denoted as follows: NS, not significant; *p* > 0.05; ^∗^*p* < 0.05; ^∗∗^*p* < 0.01; ^∗∗∗^*p* < 0.001; ^∗∗∗∗^*p* < 0.0001. Results with *p* values <0.05 were considered statistically significant.

## Resource availability

### Lead contact

Requests for further information, resources, and reagents should be directed to the lead contact, Lorenz Studer, studerl@mskcc.org.

### Materials availability

The unique cell lines generated from this study are available from the lead contact. The unmodified C9ORF72 patient and CTRL hiPSC lines were obtained from the Answer ALS via the Cedars Sinai Biomanufacturing Center.

### Data and code availability

The mass spectrometry proteomics data generated in this study have been deposited in the ProteomeXchange Consortium via the PRIDE partner repository with the dataset identifier PXD057067.

## Acknowledgments

We are grateful to the members of the Studer lab for helpful discussions and support of this study. We thank the MSKCC Gene Editing and Screening Core for their help in designing and executing the high-throughput screen (HTS). Furthermore, we thank Drs. Mary Baylies, Li Gan, and Shuibing Chen for insightful discussions. Moreover, we acknowledge BioRender.com for providing the tools to create illustrations used in this publication. We also thank Dr. Ryan Walsh for generating the GPI:H2B-Td-tomato-tagged ESCs. Additionally, we acknowledge the use of ALS patient-derived iPSC lines from the Answer ALS project, led by Cedars-Sinai Medical. We extend our deepest gratitude to the patients, their families, and the healthy control donors for their invaluable contributions to this research. The work was funded by grant AL200169 - W81XWH2110140 from the 10.13039/100000005Department of Defense and a Project ALS award to L.S. Additional support was provided by grants from the National Institutes of Health (R21NS116545, R01MH135403, core grant P30CA08748), and the JPB foundation/Freedom together Foundation to L.S. B.S. was supported by The Dompé Rita Levi Montalcini Fellowship, B.F.V. was supported by the 10.13039/501100000406Motor Neurone Disease Association (MND Association; project grant Talbot/Apr22/889-791) and a 10.13039/501100001645Boehringer Ingelheim Fonds travel fellowship, L.W. by Charles Revson fellowship, and E.G. by Rubicon fellowship (2020/30766/ZONMW).

## Author contributions

B.S. designed, performed, and interpreted most of the experiments; analyzed the data; generated the figures; and wrote the manuscript. B.F.V. performed some of the SM generation and immunofluorescent staining experiments and wrote the manuscript. L.W. performed and analyzed flow cytometry experiments. N.X. performed the spinal MN death assay and helped with the data analysis. E.L.C. and J.J. developed the two-dimensional spinal MN protocol from which the 3D spinal MN protocol is based upon. A.Z. generated the isogenic C9-ALS lines used in the screen validation. M.L.-T. and D.M.G. performed and analyzed the SM cell proportion quantifications. L.T. processed all samples for proteomic analyses. M.M. and Z.L. performed, analyzed, and interpreted the proteomics data analyses. T.Z. supervised and generated the isogenic lines used in the screen validation. E.G. conceived the idea; designed the study; developed and optimized the 3D spinal MN protocol, spinal astrocyte protocol, and SM model; and wrote the manuscript. L.S. conceived the idea, designed the study, generated funding, and wrote the manuscript.

## Declaration of interests

L.S. is a scientific cofounder and paid consultant of BlueRock Therapeutics Inc. and a scientific cofounder of DaCapo Brainscience.

## Supplemental information

Document S1. Figures S1–S4

Document S2. Article plus supplemental information
