# Supplementary material for: Telmisartan is neuroprotective in a hiPSC-derived spinal microtissue model for C9orf72 ALS via inhibition of neuroinflammation
Source: Stem Cell Reports. 2025 Jun 19;20(7):102535. doi: 10.1016/j.stemcr.2025.102535 (PMC12277806; doi:10.1016/j.stemcr.2025.102535)
Supplement: Document S1. Figures S1–S4 [file mmc1.pdf]

Stem Cell Reports, Volume *20*

Supplemental Information

Telmisartan is neuroprotective in a hiPSC-derived spinal microtissue model for C9orf72 ALS via inhibition of neuroinflammation

Berkiye Sonustun, Björn F. Vahsen, Mario Ledesma-Terrón, Zhuoning Li, Laura Tuffery, Nan Xu, Elizabeth L. Calder, Johannes Jungverdorben, Leslie Weber, Aaron Zhong, David G. Miguez, Mara Monetti, Ting Zhou, Elisa Giacomelli, and Lorenz Studer

Supplemental Information:

Telmisartan is neuroprotective in a hiPSC-derived spinal microtissue model for C9orf72-ALS via inhibition of neuroinflammation.

Berkiye Sonustun, Björn F. Vahsen, Mario Ledesma-Terrón, Zhuoning Li, Laura Tuffery , Nan Xu, Elizabeth L. Calder, Johannes Jungverdorben, Leslie Weber, Aaron Zhong, David Miguez Gómez, Mara Monetti, Ting Zhou, Elisa Giacomelli and Lorenz Studer.

- **Supplementary Methods:** Pages **1-6**

- **Supplementary Figures and Figure legends**
  - Figure S1 Pages **7-8**
  - Figure S2 Pages **9-10**
  - Figure S3 Pages **11-12**
  - Figure S4 Pages **13-14**

Supplementary Methods

hiPSC Cell Culture

Mycoplasma testing was performed upon thawing the MCB and upon receiving isogenic pairs from the MSKCC Stem Cell Core. Sterility of cultures was monitored through daily observations. Genomic characterization of MCBs was conducted by the Answer ALS repository, and karyotyping analysis was performed both when MCBs were thawed to generate WCBs (for Answer ALS and in-house isogenic pairs) and before the high-throughput screen. Confirmation of the disease mutation was performed by Answer ALS. Removal of the HRE in isogenic lines was confirmed by the MSKCC Stem Cell Core during their generation. All stem cell work was conducted in accordance with protocols approved by the Tri-Institutional Stem Cell Initiative Embryonic Stem Cell Research Oversight Committee (Tri-SCI ESCRO).

Generation of 3D, spinal motor neurons (MNs) organoids from hiPSCs in 3D

hiPSCs were dissociated using 0.04% EDTA-PBS solution and seeded at 10,000 cells/well of a V-bottom 96 well plate (S-Bio PrimeSurface® 3D culture: Ultra-low Attachment Plates, Cat # #MS-9096VZ) in E8 media containing ROCK inhibitor (Y-27632; 10 μM) on Day -1. On Days 0, 2 and 4, media was changed to Neurobasal media containing N2 and B27 supplements (NB N2/B27),

and neural induction was performed via dual SMAD inhibition with LDN193189 (250 nM; Stemgent) and SB431542 (10 µM; Tocris Bioscience) and Anterior-Posterior patterning via the Wnt-activator CHIR 99021 (3uM; Tocris). Ascorbic Acid (AA) (100 nM; Sigma) was supplemented to the media as an antioxidant. On Day 6, organoids were transferred to 10 cm dishes on an orbital shaker and maintained in NB N2/B27 media containing SB431542 (2 µM, R&D), LDN193189 (500 nM, Stemgent), AA (100 nM, Sigma), CHIR 99201 (1 µM, Tocris Bioscience), SAG (1 µM, Selleck) and Retinoic Acid (RA) (100 nM, Sigma) with media changes on days 8 and 10. On Day 11, neural progenitors were assessed for the expression of NKX 6.1 and Olig2. Within its broader expression domain, Nkx6.1 initiates transcription of Olig2 specifically in the pMN domain 64-67. Olig2 distinguishes MN progenitors from V2 interneuron progenitors that also express Nkx6.1 (Novitch et al., 2001). On Day 13, dual SMAD inhibition was completed, and cells were cultured in NB N2/B27 media containing SAG (0.5 µM, Selleck) RA (500 nM, Sigma), AA (100 nM, Sigma), BDNF (0.02 µg/ml, R&D), GDNF (0.02 µg/ml, Peprotech), and CNTF (0.01 µg/ml, R&D). On Days 15 and 17, organoids were cultured in Day 13 media supplemented with DAPT (10 µM; Tocris Bioscience). From Day 20, organoids were maintained in NB media containing B27 (NB/B27) supplemented with SAG (0.5 µM, Selleck), Retinoic Acid (RA) (500 µM, Sigma), DAPT (10 µM; Tocris Bioscience) and CNTF (0.01 µg/ml, R&D). Day 20-30 organoids were dissociated as described below and cells were subjected to quality control (QC), immunofluorescence (IF) experiments to check for Islet-1 (ISL1), HUC/D, MAP2 and TUJ1.

Generation of spinal astrocytes from hiPSCs

On Day 0, hiPSCs were dissociated into a single-cell suspension using Accutase and plated on Geltrex coated plates at 600,000 cells per cm2 in NB N2/B27 media containing SB431542 (10 µM, Tocris Bioscience), LDN193189 (250 µM, Stemgent) Ascorbic Acid (AA) (100 µM; Sigma), CHIR99201 (3 µM; Tocris Bioscience) and ROCK inhibitor (10 µM; Y-27632). Between days 1 and 5, day 0 media without ROCK inhibitor was used to culture cells with media changes every day. Between days 6 and 10, cells were cultured in NB N2/B27 media supplemented with SB431542 (2µM; Tocris Bioscience), LDN193189 (500 nM; Stemgent), AA (100 nM; Sigma), CHIR99201 (1 µM; Tocris Bioscience), SAG (1 µM; Selleck) and Retinoic Acid (RA) (100 nM; Sigma). On Day 11, neural progenitors were dissociated with Accutase solution containing DNAse I (50 µg/ml Sigma/Roche) and plated on Poly-D-lysine (10 µg/ml; Sigma) Fibronectin (2 µg/ml; Corning), Laminin (2 µg/ml; R&D) coated plates at 175,000 cells per cm2 in NB N2/B27 media containing SAG (0.5 µM; Selleck), Retinoic Acid (RA) (500 nM; Sigma), AA (100 nM; Sigma), BDNF (0.02 µg/ml, R&D) GDNF (0.02 µg/ml, Peprotech), CNTF (0.01 µg/ml, R&D) and ROCK

inhibitor (Y-27632; 10 µM). Progenitors were subjected to QC to check for NKX 6.1 and Olig2 expression via IF. On day 12, progenitors were infected with lentiviral vectors containing NFIA (https://www.addgene.org/141403/) and M2rtTA (https://www.addgene.org/20342/) overnight to induce a gliogenic switch of neural progenitors into astrocytes as described in 30 . On days 14 to 20, cells were cultured in Day 13 media supplemented with1 µg/ml Doxycycline to induce NFIA expression and on day 20, cells were subjected to QC to check for NFIA and Sox9 expression via IF. On day 22, cells were dissociated using trypsin and re-plated at 50,000 cells per cm2 in plates coated with Poly-L-Ornithine Hydrobromide (15 µg/ml; Sigma), Fibronectin (2 µg/ml; Corning) and Laminin (2 µg/ml; R&D) in Astrocyte Medium (AM, ScienCell Research Laboratories, Cat. #1801) with 2% fetal bovine serum from the AM kit, for two weeks before removing FBS from the media completely. Cryobanks were generated on Day 22, Day 35, Day 45 and Day 50 cells and cells were subjected to QC, performed by IF to check for astrocyte markers on Day 50.

Generation of microglia from hiPSCs

hiPSCs were dissociated into a single-cell suspension using Accutase on Day 0, and 60,000 cells per cm2 were seeded onto Matrigel-coated plates in Essential 8 medium with activin A (7.5 ng/ml; R&D), BMP4 (30 ng/ml; R&D), CHIR 99021 (3 µM; Tocris Bioscience) and ROCK inhibitor (Y-27632; 10 µM). Following a 16 to18 hour period, Day 0 media was changed to Essential 6 medium based Day 1 media containing activin A (10 ng/ml; R&D), BMP4 (40 ng/ml; R&D) and IWP2 (2 µM; Selleck;). On day 2, cells were exposed to FGF2 (20 ng/ml; Selleck;) that was added to the Day 1 media. The next day, cells were dissociated with Accutase and re-seeded at 60,000 cells per cm2 in Matrigel coated plates in Essential 6 medium based Day 3 media containing ROCK inhibitor (Y-27632; 10 µM). VEGF (15 ng/ml; R&D,) and FGF2 (5 ng/ml; R&D,). On Day 4, and cell medium was changed to Day 3 media without the ROCK inhibitor. Cells were exposed to Essential 6 based media containing VEGF (15 ng/ml), FGF2 (5 ng/ml), SCF (200 ng/ml; R&D) and IL-6 (20 ng/ml; R&D) on Days 5 and 6, followed by media change to Essential 6 containing SCF (100 ng/ml), IL-6 (10 ng/ml), TPO (30 ng/ml; R&D) and IL-3 (30 ng/ml; R&D) on Day 7 and Day 9. Distinctively, we did not co-culture EMP, PMACs with neurons, instead harvesting the round cells into RPMI media containing 10% FBS, and IL-34 (100 ng/ml; R&D) and M-CSF (10 ng/ml; R&D) directly for 18-20 days until round cells were adherent and exhibited classical microglia morphology and expressed microglia specific markers. Cells were subjected to QC, performed by IF to check for the expression of microglial markers and cryobanks were generated using 90% FBS and 10% DMSO as freezing media.

Dissociation of spinal MN organoids

Samples were sliced using a sterile scalpel into smaller pieces and resuspended into papain solution. The samples were incubated at 37C with constant agitation on an orbital shaker for 1 h. Following incubation, the papain-cell solutions were pipetted up and down with a micropipette (P1000) to completely dissolve any tissue pieces. The samples were then transferred to 15 mL falcon tubes and centrifuged at 300g for 5 min at room temperature. Supernatants were discarded and cell pellets were immediately resuspended in DNase dilute albumin-inhibitor solution. Subsequently, albumin-inhibitor solution was added drop-by-drop to each sample to create phase separation and tubes were centrifuged at 70g for 6 min at room temperature. Cells were resuspended in Neurobasal medium and counted to be incorporated into SMs.

Whole Mount Staining of Microtissues

Microtissues were fixed in 4% paraformaldehyde for 1 hour at 4oC, and washed 3x in PBS. After the third wash, PBS was removed and microtissues were incubated in 0.5mL of 0,5% Triton-X in PBS at RT for 6 hours. Subsequently, microtissues were re-suspended in Organoid Washing Buffer, OWB (0.2% Triton-X, 0.02% SDS and 0.2% BSA in PBS) and transferred to a low adhesion 24 well plate and incubated for 15 minutes at RT. Primary antibodies were prepared in OWB and incubated overnight on an orbital shaker at 37oC. The next day, microtissues were washed for a total of 6 hours with OWB inside the wells (3 washes, 2 hours per wash) and the secondary antibodies were prepared in OWB and added to the microtissues inside the wells and incubated overnight at 37oC on the orbital shaker. On the final day, microtissues were subjected to 3X OWB washes for a total of 6 hours, recovered with a P200 micropipette, and mounted on slides for confocal microscopy.

Cytokine Arrays

Fourteen markers were simultaneously measured in the samples using Eve Technologies' Human High Sensitivity 14-Plex Discovery Assay® (MilliporeSigma, Burlington, Massachusetts, USA, HDHSTC14) according to the manufacturer's protocol. The 14-plex consisted of GM-CSF, IFNγ, IL-1β, IL-2, IL-4, IL-5, IL-6, IL-8, IL-10, IL-12p70, IL-13, IL-17A, IL-23, TNF-α. Assay sensitivities of these markers range from 0.11 – 3.25 pg/mL for the 14-plex. Individual analyte sensitivity values are available in the MilliporeSigma MILLIPLEX® MAP protocol. The measurements were performed on a bead analyzer (Bio-Plex 200), which detects the amount of the target analyte. The results are quantified according to a standard curve.

Live imaging with BioTek Cytation 5 Cell Imaging Multimode Reader

Cells were initially seeded into 96-well plates on D0. These plates were incubated under standard cell culture conditions (37°C, 5% CO_2) until they adhered. The next day (D1), wells were treated with 0.01% DMSO or 5 μM telmisartan (final concentration in 100ul media) and incubated overnight under standard cell culture conditions. Prior to imaging, the BioTek Cytation 5 was configured to maintain the cells at 37°C and 5% CO_2 during the entire imaging process. The plates were loaded onto the Cytation 5. Imaging commenced on Day 2 after cell seeding, and the Cytation 5 was programmed to capture both brightfield and fluorescent images (targeting the Td-tomato channel) every 4 hours over a 14-day period. Exposure settings, focus parameters, and fluorescence intensity thresholds were optimized and standardized across all wells to ensure consistent image acquisition. Every 72 hours, a complete media change was performed to refresh the media containing either telmisartan or DMSO.

Gene correction and validation for C9orf72 ALS iPSC line

Two nicking sgRNAs were designed targeting to the upstream and downstream close to the repeat sequence. Each target sequence was cloned into the pX335 vector (Addgene, Plasmid #42335) to make the gene targeting constructs (Scheme: slide 3). A donor plasmid containing a 586 bp left homology arm, followed by a "loxp-PGK-puro-loxp" cassette, a normal 2 G4C2 repeat, and a 652 bp right homology arm was used as the template for HDR (Scheme: slide 3). The nicking sgRNAs and the donor plasmid were electroporated into patients' iPSCs using a Lonza 4D-Nucleofector instrument with Solution "Primary Cell P3", and Pulse Code "CB-150". 0.5 ug/ml Puromycin was added to the 3 days post-electroporation cells for 4 days. Single-cell clones were then generated, PCR and Sanger-sequencing33 were used to identify the correctly knock-in clones that Knock-in to the mutant allele, and with the WT allele untouched. For the identified corrected Knock-in clones, Cre treatment to remove the "loxp-PGK-puro-loxp" cassette and left a "loxP" and a normal 2 G4C2 repeat at the genome locus.

sgRNA sequences and PCR primers:

C9orf72- up sgRNA target	GCTCTCACAGTACTCGCTGA
C9orf72- down sgRNA target	GAAAGCCCGACACCCAGCTT
C9orf72-PCR-F1	AGGTGTAGACGTTGAGAGCC
C9orf72-PCR-R1	CTGAAATTGTGCAGGCGTCT

3D Image Processing and Analysis

Kernel Size Determination: Kernel size, a critical parameter for image filtering and analysis, was estimated using a two-step approach. Initially, a difference image was generated by subtracting the average z-projection from the maximum z-projection. Median filtering and Euclidean Distance Transform (EDT) were applied to this image, with local maxima in the EDT corresponding to initial kernel size estimates. To refine this estimate, the top 5% densest image slices were subjected to background subtraction and median filtering using the initial kernel size. Local maxima in the EDT of these refined slices provided the final kernel size for subsequent image processing.

Nuclear Segmentation and Characterization: DAPI staining was used to identify and localize nuclei within the image volume. To enhance image quality, DAPI images underwent Gaussian blurring and Unsharp Masking. A combination of thresholding, background subtraction, and watershed segmentation was employed to delineate individual nuclei. The resulting 3D nuclear objects were characterized in terms of their position, orientation, and dimensions using an algorithm of clustering in 3D described in Ledesma-Terrón et al.34.

Marker Analysis and Quantification: Isl1, Iba1, and GFAP channels were subjected to median filtering, with a global intensity threshold differentiating signal from background. To reduce noise in the GFAP channel, a grayscale opening operation was applied. To extract quantitative information, the mean marker intensity inside each nuclear volume is looked for all markers, and phenotypes were assigned based on comparative analysis of standardized intensities for each marker.

Data Analysis: To assess temporal changes in marker expression, data were categorized based on experimental conditions (C9-ALS+DMSO, C9-ALS + telmisartan, CTRL+DMSO, CTRL + telmisartan). Statistical comparisons of mean marker intensities across time points were performed to identify significant differences.

Supplemental Figure S1 (Sonustun et al.,)

A

iPSC Line Name	Disease	Parent Cell Type	Sex	Revised El Escorial Criteria	Age at Sample Collection	Age at Death	Cause of Death	Baseline ALSFRS-R
CS7VCZi ALS-n3	C9ORF72 ALS	PBMC/ T-Cell	Male	Definite	64	65	Disease Progression	-2.9324
CS5DZLi CTR-n5	Healthy CTRL	PBMC	Male	N/A	64	N/A	N/A	N/A

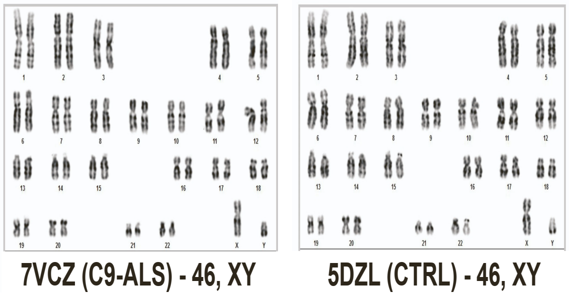

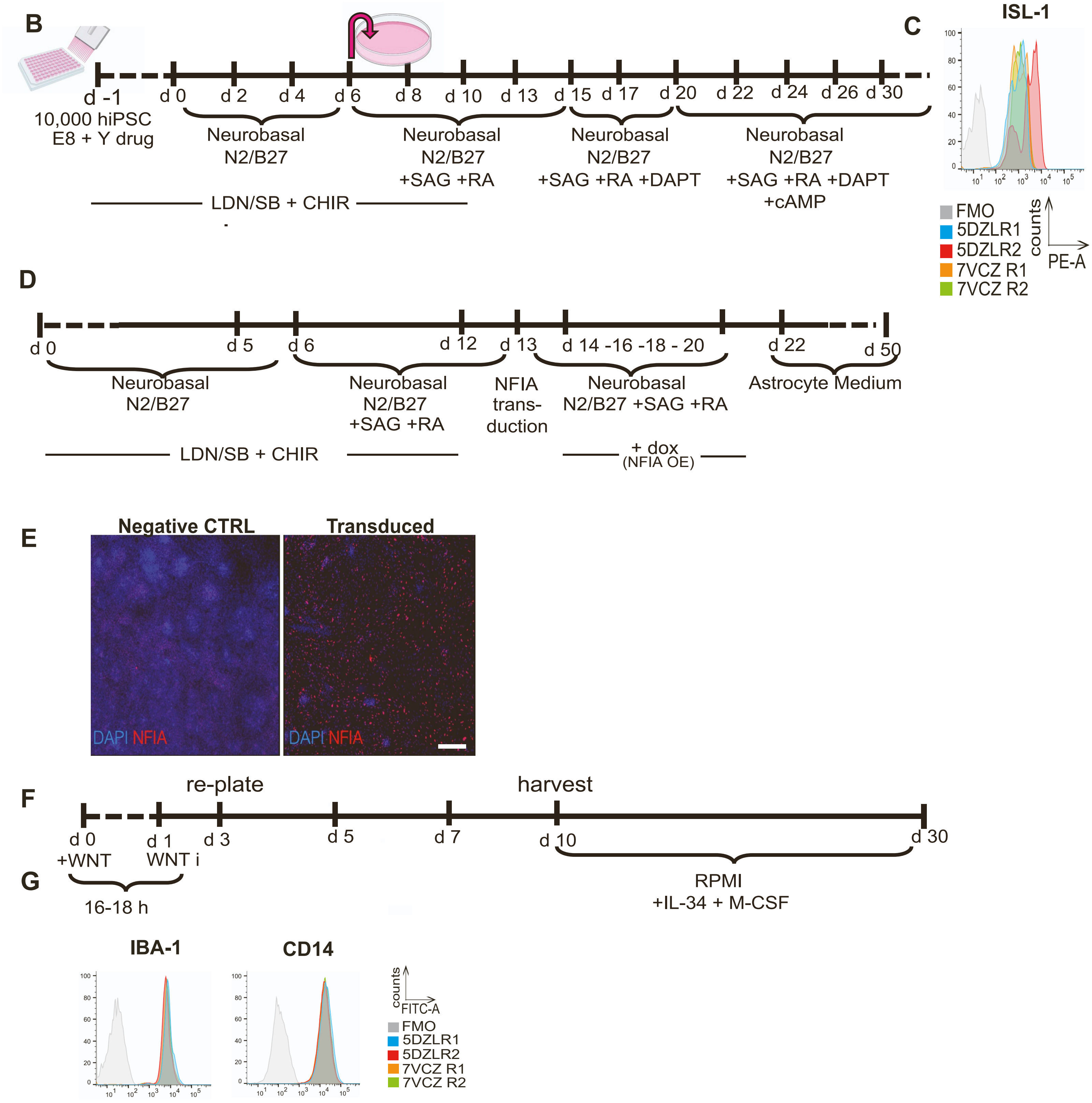

Figure S1. Characterization of hiPSC-Derived Spinal Microtissues – related to Fig. 1

(A) Details and karyotype analysis of the C9-ALS and age- and gender-matched healthy control (CTRL) iPSC lines used in this study. The C9-ALS iPSC lines were derived from patients diagnosed with ALS harboring the C9orf72 hexanucleotide repeat expansion, while the healthy CTRL iPSC lines were obtained from individuals without neurodegenerative diseases.

(B) Schematic of spinal motor neuron (MN) differentiation from hiPSCs in 3D culture. Starting with 10,000 cells per well in a low-attachment 96-well V-bottom plate, the protocol involves dual-SMAD inhibition and anterior-posterior and ventral patterning, resulting in high-purity ISL-1 positive 3D spinal MN organoids within 20-30 days.

(C) Representative flow cytometry analysis showing ISL-1 positivity in Day 30 (d30) hiPSC-derived spinal MN organoids from both healthy control and C9-ALS patients following papain dissociation. (FMO: fluorescence minus one).

(D) Schematic representing the hiPSC-derived spinal astrocyte differentiation protocol adapted from Tchieu et al. Spinal astrocytes are obtained by overexpressing NFIA in NSCs, promoting a glial fate switch in progenitors.

(E) Immunofluorescence characterization of NFIA transduction efficiency in neural progenitor cells (NPCs) on Day 20 (D20). The left panel shows NPCs transduced with a non-targeting virus, stained with DAPI (blue) for nuclear visualization and NFIA (red) as a control, indicating minimal or no expression of NFIA. The right panel displays NPCs transduced with the NFIA-targeting virus, demonstrating successful transduction, as evidenced by the widespread presence of NFIA-positive (red) nuclei throughout the culture. Scale bar corresponds to 100μm.

(F) Schematic illustrating the hiPSC-derived microglia differentiation protocol adapted from Guttikonda et al. The protocol directs hiPSCs to differentiate into microglial precursors by mimicking primitive hematopoiesis. Hemangioblast precursors are exposed to hematopoietic cytokines, generating macrophage precursors that transition into microglia expressing markers such as Pu.1 and IBA-1.

(G) Flow cytometry analysis showing IBA-1 and CD14 positivity in Day 30 (d30) hiPSC-derived microglia from healthy control and C9-ALS following Accutase dissociation. (FMO: fluorescence minus one).

Supplemental Figure S2 (Sonustun et al.,)

A

Abbreviation	Name
UK	UK 14,304
BRC	Bromocriptine mesylate
DHE	Dihydroergotamine mesylate
DBT	Dobutamine hydrochloride
MXD	Minoxidil
PRZ	Prazosin hydrochloride
DIL	Diltiazem hydrochloride
DIP	Dipyridamole
CYT	Cyclothiazide
FMB	Felbamate
CIM	Cimetidine
TPM	Tropicamide
GBC	Glibenclamide
PMZ	Pimozide
DXZ	Diazoxide
TMX	Tamoxifen citrate
ICI	ICI 182,780
TXL	Taxol
DEX	Dexamethasone
ETP	Etoposide

Abbreviation	Name
FMZ	Flumazenil
CLF	Clemastine fumarate
FLC	Flecainide acetate
MFP	Mifepristone
MPA	Mycophenolic acid
LOV	Lovastatin
ARG	Argatroban
CLZ	Cilostazol
CSP	Cisapride
ACT	Acetaminophen
LRT	Loratidine
SIM	Simvastatin
ISR	Isradipine
FLP	Fluticasone propionate
MRP	Mirtazapine
TBZ	Tetrabenazine
DOX	Doxorubicin hydrochloride
RAL	Raloxifene hydrochloride
FLX	Fluoxetine hydrochloride
FEX	Fexofenadine hydrochloride

Abbreviation	Name
ACV	Acyclovir
AML	Amlodipine besylate
BEN	Benazepril hydrochloride
MEX	Mexiletine hydrochloride
CLF	Clofarabine
DCT	Decitabine
ZON	Zonisamide
CAB	Cabergoline
BDS	Budesonide
ACR	Acarbose
PBA	Sodium 4-Phenylbutyrate
CAR	Carvedilol
CPT	CPT 11
TMZ	Temozolomide
DMD	Dexmedetomidine hydrochloride
IBU	(S)-(+)-Ibuprofen
LVT	Levetiracetam
ADP	Adapalene
MET	Metformin hydrochloride
VEN	Venlafaxine hydrochloride

Abbreviation	Name
FLD	Felodipine
DOX	Doxazosin mesylate
SPR	Spironolactone
IRE	Iressa
MCZ	Miconazole nitrate
TAM	Tamsulosin hydrochloride
BMT	Bumetanide
FRS	Furosemide
RAN	Ranolazine dihydrochloride
MTP	Metoprolol tartrate
GEM	Gemcitabine hydrochloride
VPA	Valproic acid, sodium salt
MTP	Metyrapone
ZLT	Zileuton
FLV	Fluvastatin sodium
SLX	Selexipag
FLD	Fludarabine
NEB	Nebivolol hydrochloride
SUM	Sumatriptan succinate
TIZ	Tizanidine hydrochloride

Abbreviation	Name
TOP	Topiramate
ASN	Asenapine maleate
MRV	Maraviroc
DFT	Dofetilide
EXE	Exemestane
VAR	Varenicline tartrate
LNZ	Linezolid
SUN	Sunitinib malate
AZM	Azithromycin
SLF	Sildenafil citrate
DOC	Docetaxel
LST	Losartan potassium
RPG	Repaglinide
CP	CP 690550 citrate
TRN	Tranylcypromine hydrochloride
ABT	ABT 199
TRV	Trovafloxacin mesylate
RTG	Rotigotine hydrochloride
IBL	Ibutilide hemifumarate
PYM	Pyrimethamine

Abbreviation	Name
ASP	Aspirin
HCT	Hydrocortisone
FLU	Flutamide
CLT	Clotrimazole
CRB	Carbamazepine
AZA	Azathioprine
MMF	Mycophenolate mofetil
PRB	Probenecid
FFB	Fenofibrate
BPR	Bepridil hydrochloride
PIO	Pioglitazone hydrochloride
ABA	Abacavir hemisulfate
DEL	Delavirdine mesylate
VAL	Valsartan
NTL	Nateglinide
OLO	Olopatadine hydrochloride
MCL	Meclizine dihydrochloride
TGB	Tiagabine hydrochloride
RSG	Rasagiline mesylate
OLZ	Olanzapine

Abbreviation	Name
AXI	Axitinib
SQV	Saquinavir mesylate
RVG	Rivastigmine tartrate
TFT	Trifluorothymidine
RBV	Ribavirin
AZL	Azilsartan
OLM	Olmesartan
SAHA	SAHA
ZLM	Zolmitriptan
SLT	Salmeterol xinafoate
QTP	Quetiapine hemifumarate
CND	Candesartan
DLX	(S)-Duloxetine hydrochloride
CAP	Capecitabine
MPS	Methyl-prednisolone
LNG	Levonorgestrel
AMX	Amlexanox
RUX	Ruxolitinib
PTV	Pitavastatin calcium
MGT	Miglitol

Abbreviation	Name
STV	Stavudine
RZT	Rizatriptan benzoate
TLM	Telmisartan
TLV	Tolvaptan
RMP	Ramipril
BZA	Bazedoxifene acetate
RSG	Rosiglitazone
RFX	Rifaximin
XL	XL 184
ARP	Aripiprazole
REM	Remdesivir
IRB	Irbesartan
AMB	Ambrisentan
RTV	Ritonavir
IMT	Imatinib mesylate
FTY	FTY 720
PMT	Pemetrexed
ARM	Arformoterol tartrate
RUC	Rucaparib camsylate
BOS	Bosentan

Abbreviation	Name
ANZ	Anastrozole
BCL	Bicalutamide
BOS	Bosutinib
LTZ	Letrozole
FBS	Febuxostat
ESC	Escitalopram oxalate
TRF	Teriflunomide
ALK	Aliskiren hemifumarate
VLZ	Vilazodone hydrochloride
SAX	Saxagliptin hydrochloride
PF	PF 04449913 maleate
DRV	Darunavir
DST	Dasatinib
IBR	Ibrutinib
ETV	Entecavir
MGA	Megestrol Acetate
LND	Lenalidomide
TDL	Tadalafil
PRG	Prasugrel
TRZ	Trazodone hydrochloride

Abbreviation	Name
RSV	Rosuvastatin calcium
CCS	Ciclesonide
CCP	Ciclopirox
PNT	Phentolamine Mesylate
APR	Aprepitant
RFL	Roflumilast
SLN	Silodosin
LPT	Lapatinib
SRF	Sorafenib
MFQ	Mefloquine hydrochloride

B

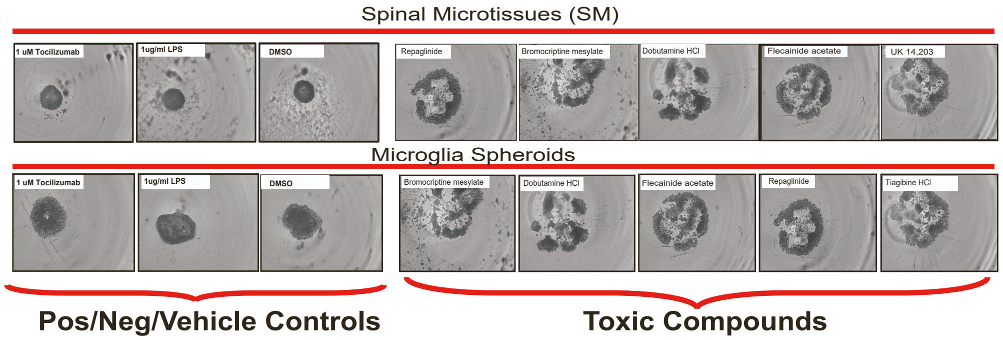

C

	Condition	Z' Score
IL-6	Microglia Spheroids	0.853
	Spinal Microtissues	0.6889
IL-8	Microglia Spheroids	0.7625
	Spinal Microtissues	0.9127

D

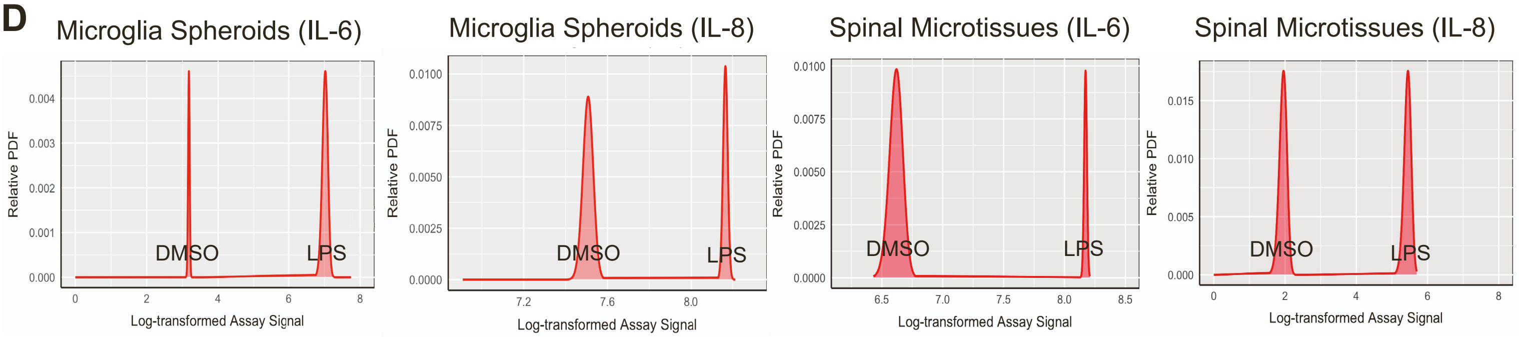

E

Drug	Condition	EC50
Telmisartan	IL-6	1.2 µM
	IL-8	3.9 µM
Valsartan	IL-6	9.7 µM
	IL-8	15 µM
Azilsartan	IL-6	0.16 µM
	IL-8	3.2 µM

Figure S2. Assay Validity and details about compounds – related to Fig. 2

(A) Table showing compound abbreviations in 3-letter codes used in the screen. This table provides a reference for interpreting the hit identification in Figure 2(d).

(B) Cell viability was assessed by measuring the microtissue/spheroid diameters before and 72 hours after library application, and toxic compounds were excluded from analysis. Representative images of toxicity, as well as cells treated with the positive, negative and vehicle controls are shown.

(C) Z' scores for IL-6 and IL-8 cytokine assays conducted on microglia spheroids and spinal microtissues.

(D) Z-prime factor graphs were generated based on relative probability distribution factors on the y-axis, and the extrapolated assay signals of negative and vehicle control data on the x-axis. The peaks represent the separation between the positive control (LPS, right peak) and the vehicle control (DMSO, left peak). These graphs utilize the standard deviations, means, and medians of the control data to depict the assay performance. Z-prime scores and assay validity for the high-throughput screen of 190 FDA-approved compounds targeting neuroinflammation in C9-ALS. All conditions yielded Z-prime factors of above 0.5 indicating high assay quality and reliability.

Supplemental Figure S3 (Sonustun et al.,)

A

Control Microglia Spheroids

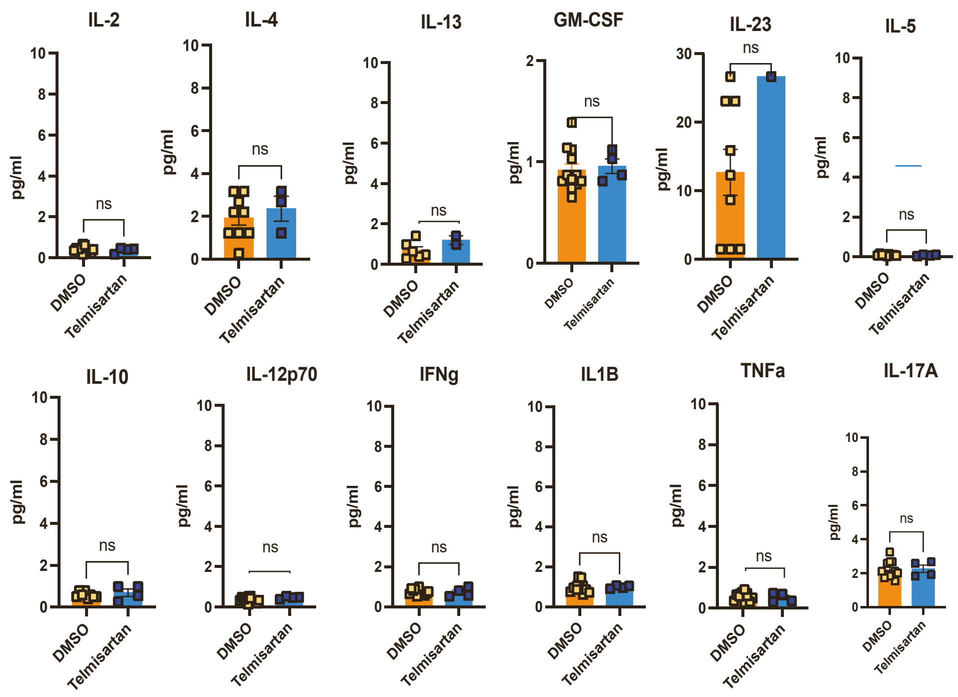

B

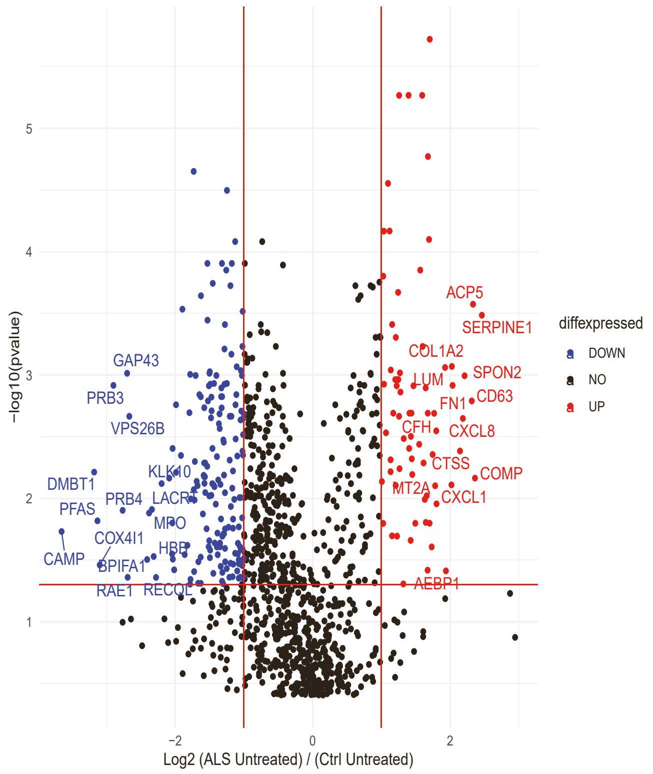

C

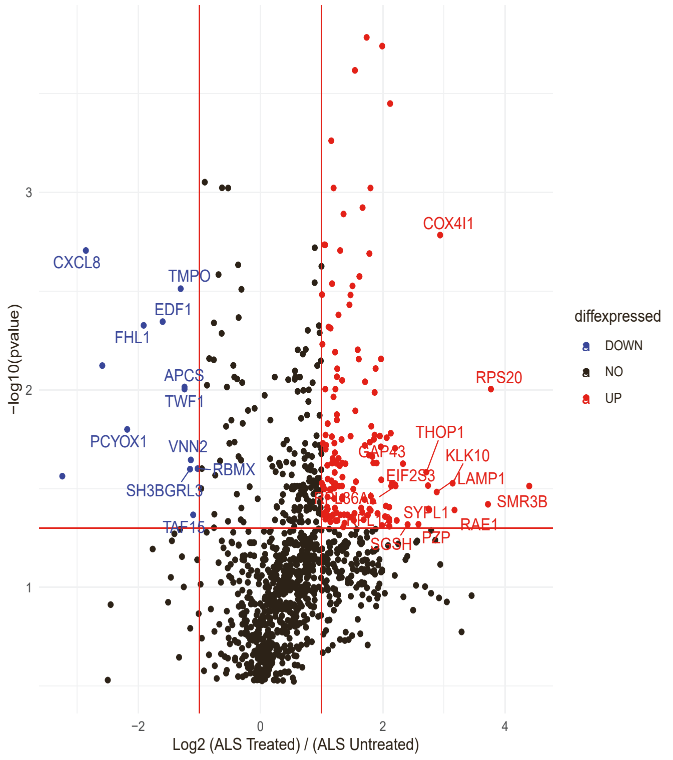

Figure S3. Telmisartan does not affect cytokine levels in supernatant of CTRL SMs - related to Fig. 3

(A) Bar graphs depicting cytokine concentrations from untreated and 5 μM telmisartan treated CTRL SMs. Results show that Telmisartan treatment shows no impact on cytokine levels in CTRL SMs. Student's T-Test N = 3 replicates, 3 independent differentiations. Data are represented as Mean ± SEM.

(B) Unbiased secretome proteomic analysis of untreated C9-ALS and control SM supernatants. Volcano plot depicting significantly different proteins between C9-ALS and control SMs (blue = significantly downregulated proteins in C9-ALS, red = significantly upregulated proteins in C9-ALS compared to controls). IL-8 (CXCL8) is among the top upregulated proteins in the C9-ALS secretome compared to controls, validating our findings. NB: 'Untreated' refers to DMSO-treated.

(C) Unbiased secretome proteomic analysis of untreated C9-ALS and C9-ALS SMs treated with 5 μM Telmisartan. Volcano plot depicting significantly different proteins between untreated C9-ALS and Telmisartan-treated C9-ALS SMs (blue = significantly downregulated proteins in C9-ALS + telmisartan, red = significantly upregulated proteins in C9-ALS + telmisartan compared to untreated C9-ALS). IL-8 (CXCL8) is the top downregulated protein in the telmisartan-treated C9-ALS secretome compared to untreated C9-ALS, validating our HTS findings. NB: 'Untreated' refers to DMSO-treated.

Supplemental Figure S4 (Sonustun et al.,)

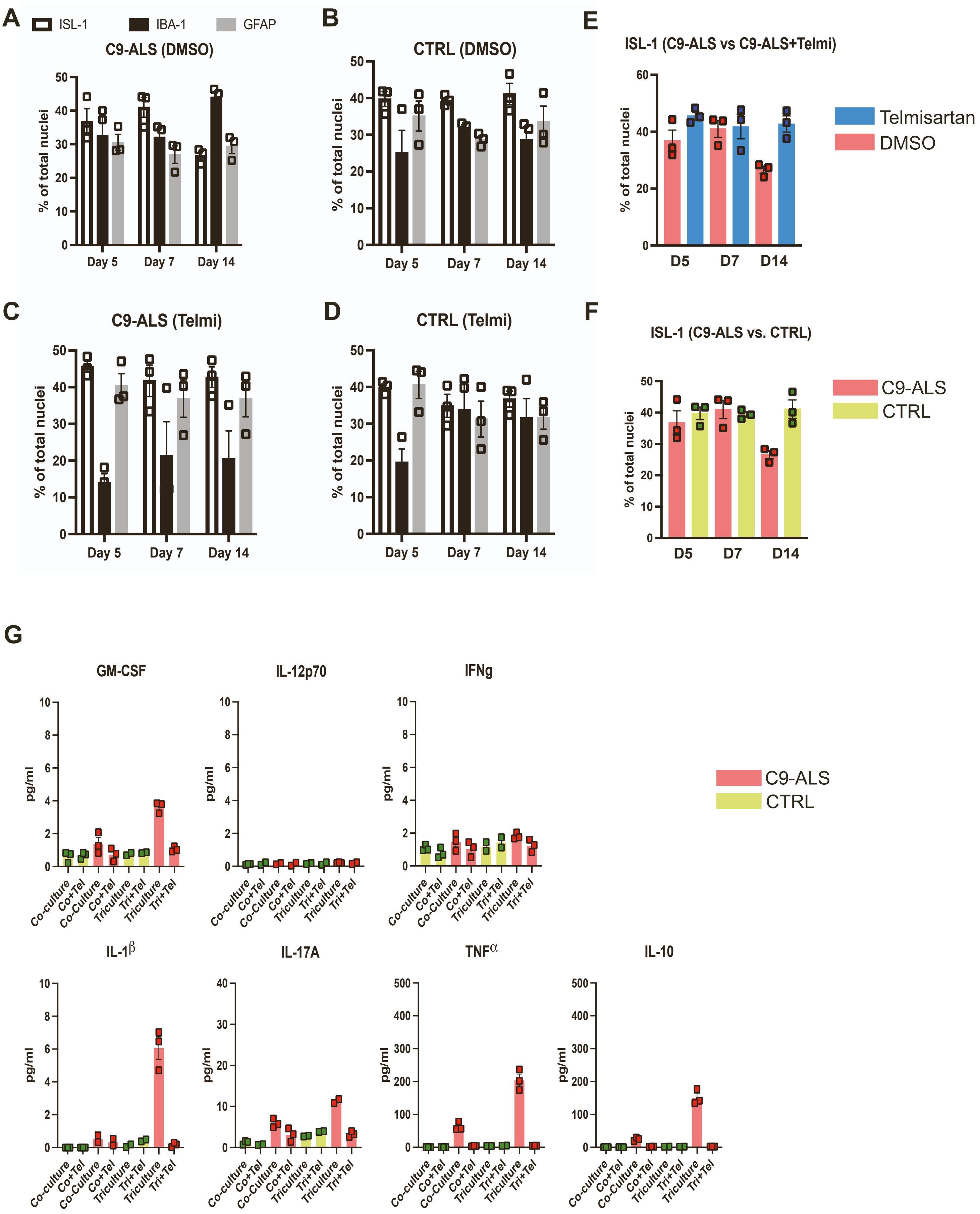

Figure S4. Time-Dependent Effects of Telmisartan on Cell Proportions in C9-ALS Spinal Microtissues – related to Fig. 4

(A)-(D) The bar graphs depict the proportions of IBA1+ microglia, GFAP+ astrocytes, and ISL-1+ spinal motor neurons (MNs) in C9-ALS (red) and control (CTRL, green) spinal microtissues treated with telmisartan (Telmi) over 14 days. The data is shown for Day 5 (D5), Day 7 (D7), and Day 14 (D14).

(E) ISL-1 proportion comparisons between C9-ALS SM +DMSO vs. C9-ALS SM + telmisartan represented as % nuclei of total.

(F) ISL-1 proportion comparisons between C9-ALS SM +DMSO vs. CTRL SM + DMSO represented as % nuclei of total.

(G) Bar graphs depicting the effect of telmisartan on the levels of GM-CSF, IL-10, IL-1β, TNFα, and IL-17A from the supernatants of C9-ALS co- and tri- cultures at 336 hours.

The data presented in this figure were generated using confocal microscopy on whole-mount stained SMs. The quantifications in these panels are based on the z-stack imaging, which captures the three-dimensional architecture of the SMs. The analysis paradigms are described in the Methods section in detail. N = 3 technical replicates, generated using 2 differentiations from 1 C9-ALS line and its isogenic control. Data are represented as Mean ± SEM.
