# Supplementary material for: Telmisartan is neuroprotective in a hiPSC-derived spinal microtissue model for C9orf72 ALS via inhibition of neuroinflammation
Source: Stem Cell Reports. 2025 Jun 19;20(7):102535. doi: 10.1016/j.stemcr.2025.102535 (PMC12277806; doi:10.1016/j.stemcr.2025.102535)
Supplement: Document S2. Article plus supplemental information [file mmc2.pdf]

Stem Cell Reports
Article
OPEN ACCESS

Telmisartan is neuroprotective in a hiPSC-derived spinal microtissue model for C9orf72 ALS via inhibition of neuroinflammation

Berkiye Sonustun,[1,2] Björn F. Vahsen,[3,4] Mario Ledesma-Terrón,[5] Zhuoning Li,[6] Laura Tuffery,[6] Nan Xu,[1,7] Elizabeth L. Calder,[1,10] Johannes Jungverdorben,[1] Leslie Weber,[1] Aaron Zhong,[8] David G. Miguez,[5] Mara Monetti,[6] Ting Zhou,[1,8] Elisa Giacomelli,[1,9,11,*] and Lorenz Studer[1,2,11,12,*]

[1]Developmental Biology Program & Center for Stem Cell Biology, Memorial Sloan Kettering Cancer Center, New York, NY 10065, USA
[2]Weill Cornell Graduate School of Medical Sciences, Cornell University, New York, NY 10065, USA
[3]Oxford Motor Neuron Disease Centre, Nuffield Department of Clinical Neurosciences, University of Oxford, John Radcliffe Hospital, Oxford OX3 9DU, UK
[4]Kavli Institute for Nanoscience Discovery, University of Oxford, Dorothy Crowfoot Hodgkin Building, OX1 3QU Oxford, UK
[5]Centro de Biologia Molecular Severo Ochoa, Departamento de Fisica de la Materia Condensada, Instituto Nicolas Cabrera, and Condensed Matter Physics Center (IFIMAC), Universidad Autonoma de Madrid, 28049 Madrid, Spain
[6]Proteomics Innovation Laboratory and Proteomics Core, Memorial Sloan Kettering Cancer Center, New York, NY 10065, USA
[7]Louis V. Gerstner Jr. Graduate School of Biomedical Sciences, New York, NY 10065, USA
[8]The SKI Stem Cell Research Facility, The Center for Stem Cell Biology and Developmental Biology Program, Sloan Kettering Institute, 1275 York Avenue, New York, NY 10065, USA
[9]Sean M. Healey and the AMG Center for ALS and the Neurological Clinical Research Institute, Massachusetts General Hospital, Harvard Medical School, Boston, MA, USA
[10]Present address: DaCapo Brainscience Inc., 700 Main Street North, Cambridge, MA 02139, USA
[11]These authors contributed equally
[12]Lead contact
*Correspondence: egiacomelli@mgh.harvard.edu (E.G.), studerl@mskcc.org (L.S.)
https://doi.org/10.1016/j.stemcr.2025.102535

SUMMARY

Amyotrophic lateral sclerosis (ALS) is a fatal neurodegenerative disease characterized by progressive motor neuron (MN) loss. The most common genetic cause, a hexanucleotide repeat expansion in *C9orf72* (C9-ALS), disrupts microglial function, contributing to neuroinflammation, a key disease driver. To investigate this, we developed a three-dimensional spinal microtissue (SM) model incorporating human induced pluripotent stem cell (hiPSC)-derived MNs, astrocytes, and microglia. Screening 190 Food and Drug Administration (FDA)-approved compounds, we identified sartans—angiotensin II receptor I blockers (ARBs)—as potent inhibitors of neuroinflammation. Telmisartan, a highly brain-penetrant ARB, significantly reduced the levels of pro-inflammatory cytokines interleukin (IL)-6 and IL-8 and rescued MN loss in C9-ALS SMs. Our findings suggest that C9-ALS microglia drive MN toxicity and that telmisartan can effectively mitigate inflammation and preserve MN viability. This work lays the groundwork for modeling disease-related neuroinflammation and points to telmisartan as a therapeutic candidate worth further exploration for treating C9-ALS.

INTRODUCTION

Amyotrophic lateral sclerosis (ALS) is a fatal neurodegenerative disease characterized by motor neuron (MN) degeneration in the brain and spinal cord, leading to progressive paralysis and death within 3–5 years (Lall and Baloh, 2017). While most ALS cases lack an identifiable genetic cause, the expansion of the G_4C_2 hexanucleotide repeats (GGGGCC) within the first intron of the chromosome 9 open reading frame 72 (*C9orf72*) gene contributes to approximately 25%–40% of familial cases and 5% of sporadic ALS/FTD cases (Balendra and Isaacs, 2018; Boillée et al., 2006). The *C9orf72* gene is implicated in various physiological processes, particularly in microglial and myeloid cells, and in pathways related to immune regulation and glial cell functions (Balendra and Isaacs, 2018; Christoforidou et al., 2020; Masrori et al., 2022). Glial cell activation and dysfunction of astrocytes and microglia have been linked to both familial and sporadic ALS (Conlon et al., 2018) based on studies in postmortem tissue and in mouse and cellular models of the disease (Balendra and Isaacs, 2018; Boillée et al., 2006; Boillée et al., 2006; Christoforidou et al., 2020; Masrori et al., 2022; Saxena and Caroni, 2011). Neuroinflammation may be particularly important for driving ALS disease progression (Philips and Rothstein, 2014) and thereby presents a promising target for therapeutic intervention. However, there is a pressing need for a robust, scalable, and physiologically relevant model of human neuroinflammation to identify relevant candidate therapeutics.

Human pluripotent stem cells (hPSCs) offer a unique opportunity to capture neuro-glial interactions in relevant human cell types using patient-specific or genetically engineered stem cell lines carrying patient-related mutations. There is long-standing evidence that glial cells contribute to disease phenotypes as first described for SOD1 mutant ALS models (Di Giorgio et al., 2007; Nagai et al., 2007). *C9orf72* ALS (C9-ALS) models show phenotypes such as RNA foci or dipeptide repeat-related pathologies in neurons as reviewed recently (Giacomelli et al., 2022)

Published by Elsevier Inc. on behalf of International Society for Stem Cell Research.
This is an open access article under the CC BY license (http://creativecommons.org/licenses/by/4.0/).

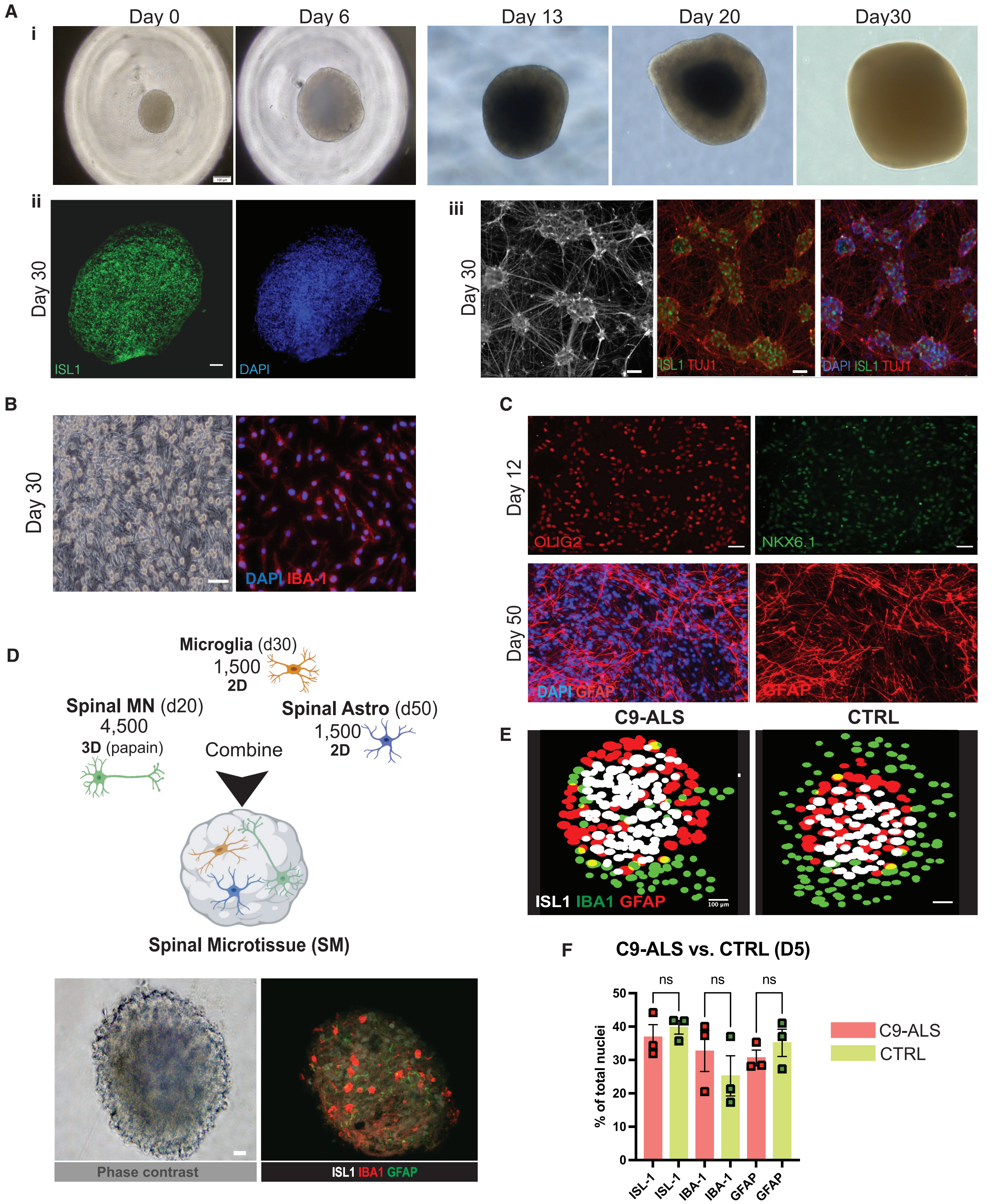

Figure 1. Characterization and workflow of hiPSC-derived spinal microtissues

(A) (i) Representative bright-field images capture the 30-day development of 3D spinal motor neuron (MN) organoids, showing the transition from initial cell aggregation (day 0) to well-defined structures with increasing complexity at days 5, 10, 20, and 30. (ii) A

(legend continued on next page)

including neurotoxic effects mediated by C9-microglia in co-culture platforms (Vahsen et al., 2022).

Here, we developed a scalable 3D spinal microtissue (SM) model integrating human induced pluripotent stem cell (hiPSC)-derived MNs, astrocytes, and microglia. C9-SMs showed elevated levels of secreted interleukin-6 (IL-6) and IL-8 compared to control (CTRL) SMs, reflecting a C9-ALS neuroinflammatory signature. Utilizing this inflammatory readout, we screened 190 Food and Drug Administration (FDA)-approved compounds; identified angiotensin II receptor I (AT1R) blockers (ARBs) as modulators of this neuroinflammatory signature; and demonstrated telmisartan's ability to reduce inflammation and prevent MN loss.

RESULTS

Development of a 3D SM triculture model

To generate a 3D, multicellular SM model from hiPSCs, we first optimized two-dimensional (2D; monolayer) and 3D (organoid) protocols to direct differentiation into spinal motor neurons (spinal MNs), spinal astrocytes, and microglia. For these studies, we used patient C9-ALS hiPSCs and age- and gender-matched, healthy CTRL lines. Details on these cell lines are presented in Figure S1A. Spinal MNs were generated using a novel, highly efficient, and scalable 3D organoid protocol (Figure 1Ai) detailed in Figure S1B. This allowed us to generate high-purity spinal MNs (Figure S1C) between day 20–30 of differentiation (Figure 1Aii). Dissociated spinal MNs display typical neuronal morphology and express MN markers such as ISL-1 (Figure 1Aiii). For generating hiPSC-derived microglia, we used an adaptation of a previous protocol (Guttikonda et al., 2021) (Figures 1B and S1F). The resulting microglia are characterized by the presence of IBA-1 (Figure 1B) and CD14 (Figure S1G) on day 30. To obtain spinal cord astrocytes (spinal astrocytes), we directed the hiPSCs toward a spinal cord progenitor identity and transduced progenitors with nuclear factor I/A (NFIA), which yielded GFAP-positive astrocytes by day 50 (Figure 1C). Details of this protocol are summarized in Figure S1D. Day 50 spinal astrocytes showed characteristic morphology and expressed astrocyte makers such as GFAP, and their spinal identity was confirmed by the presence of Nkx6.1 and Olig2 by immunofluorescence (IF) (Figure 1C). All three cell types were generated from either C9-ALS or CTRL lines.

Next, spinal MNs, spinal astrocytes, and microglia from C9-ALS and CTRL hiPSCs were combined in suspension in 96-well V-bottom plates, as previously described for cardiac microtissues (Giacomelli et al., 2020) to generate a 3D, triculture platform, herein referred to as "spinal microtissue" (SM). Each SM is composed of 7,500 cells in total, consisting of 1,500 spinal astrocytes, 4,500 spinal MNs, and 1,500 microglia, ultimately forming a 1:3:1 ratio (Figure 1D) The cell composition of SMs was validated over time by assessing marker expression (ISL-1, IBA-1, and GFAP). No significant differences among cell types were found between C9-ALS and CTRL SMs on D5 (Figures 1E and 1F).

Sartans decrease neuroinflammation in ALS SMs from C9orf72 hiPSC lines

Cytokine profiling in C9-ALS and CTRL SM supernatants was performed to detect supernatant concentrations and fluorescence intensity of 14 inflammatory cytokines (Figure 2A). We selected IL-6 and IL-8 as inflammatory readouts for our compound screen, as they showed the best signal to noise ratio. Next, we performed a chemical screen of 190 FDA-approved compounds to identify modulators of neuroinflammation exclusively in C9-ALS SMs, using the

cryosectioned hiPSC-derived spinal MN organoid at day 30 shows ISL-1 expression (green) with nuclear staining (DAPI, blue). (iii) Five days after papain dissociation, bright-field and immunofluorescence images display ISL-1 (green), TUJ1 (red), and DAPI (blue) at day 30.
(B) Representative images of IBA-1-positive day 30 hiPSC-derived microglia in monolayer culture, including a corresponding bright-field image.
(C) Representative images of hiPSC-derived neural stem cells (NSCs) on day 12 of differentiation, depicting NKX6.1 and OLIG2 staining (top panel). Bottom panel shows differentiated GFAP-positive spinal astrocytes on day 50. Scale bars represent 100 μm unless otherwise indicated.
(D) A schematic illustrates the workflow for generating spinal microtissues (SMs) by combining spinal MNs (days 20–30), spinal astrocytes (day 50), and microglia (day 30). Cells were mixed in defined ratios, seeded into low-attachment 96-well V-bottom plates, centrifuged (10 min at 1,090 rpm), and incubated overnight at 37°C. Bright-field and immunofluorescence images at day 5 confirm SM integrity and composition.
(E) Representative image outputs of SMs using the Object Segmentation, Counter and Analysis Resource (OSCAR) pipeline transformed into 2D representations depicting the three cell types as white (spinal MN), green (microglia), and red (spinal astrocytes).
(F) Quantitative analysis of SMs reveals no significant differences in cell proportions between C9-ALS and CTRL on day 5 post-SM generation. The data presented in this figure were generated using confocal microscopy on whole-mount stained SMs. The quantifications are based on the z stack imaging, which captures the 3D architecture of the SMs as described in the methods section.
Statistical analysis was performed using one-way ANOVA with Šídák's multiple comparisons test. ns, not significant. $*p < 0.05$, $**p < 0.01$. Cumulative analysis from $N = 3$ differentiations. Data are shown as mean ± SEM.

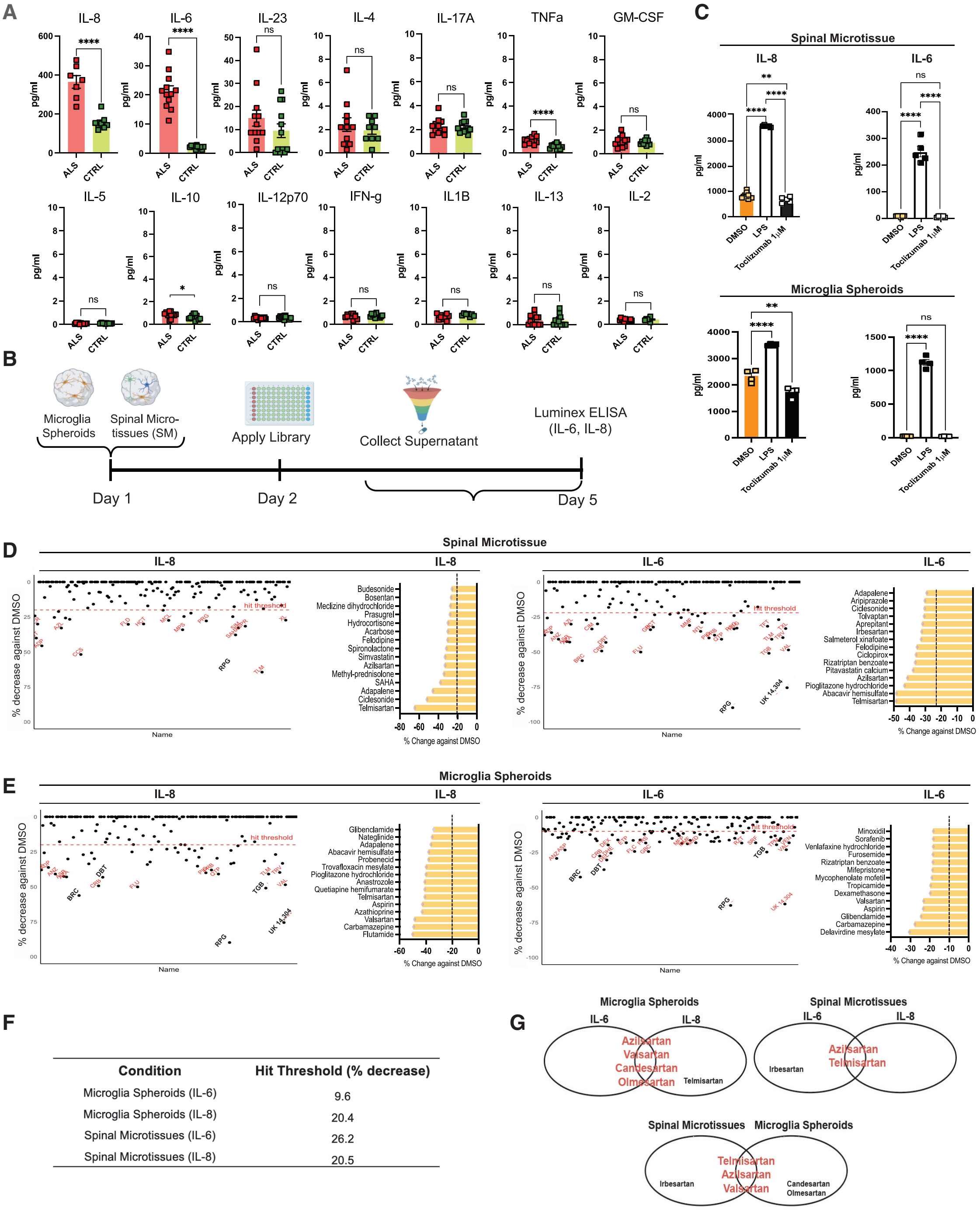

Condition	Hit Threshold (% decrease)
Microglia Spheroids (IL-6)	9.6
Microglia Spheroids (IL-8)	20.4
Spinal Microtissues (IL-6)	26.2
Spinal Microtissues (IL-8)	20.5

Figure 2. High-throughput screen of 190 FDA-approved compounds identifies angiotensin II receptor I blockers as modulators of neuroinflammation in C9-ALS

(A) C9-ALS and CTRL spinal microtissues (SMs) were cultured for 5 days, and supernatants were collected on day 5. IL-6 and IL-8 were identified as C9-ALS-associated inflammatory signatures by using multiplex cytokine profiling from the supernatants. $N = 2$ independent differentiations, two C9-ALS patient lines, 3 technical replicates per differentiation. Data are shown cumulatively as mean ± SEM.

(B) Experimental design highlighting the SM and microglia spheroid generation, treatment paradigm, and experiment timeline. HTS was performed on C9-ALS spinal microtissues, as well as microglia spheroids. After generating the microtissues/spheroids on D1, the

(legend continued on next page)

TocriScreen FDA-Approved Drugs Library (Figure S2A). Because our pilot experiments strongly suggested a key role for microglia in driving IL-6 and IL-8 secretion in C9-ALS, the screen was performed in both C9-ALS SMs and in C9-ALS microglia similarly aggregated in 3D (microglia spheroids containing 7,500 cells/spheroid). At 24 h after microtissue generation (day 2), the chemical library was applied at 5 μM final concentration for each compound, and supernatants were collected 72 h later (day 5) (Figure 2B). The screen was performed in ultra-low-attachment 96-well V-bottom plates in single wells for the compounds and triplicates for the positive, negative, and vehicle CTRLs. IL-6 and IL-8 secretion were used as a readout for C9-ALS neuroinflammatory signature. Lipopolysaccharide (LPS; 1 μg/mL) was used as the positive CTRL needed to induce the opposite effect—namely, increasing inflammation. Tocilizumab (1 μM), a soluble IL-6 receptor (sIL6R) blocking antibody, was used as negative CTRL. By blocking trans-IL-6 signaling, we anticipated that it would reduce both IL-6 and IL-8 levels. 0.01% DMSO was used as vehicle CTRL. Tocilizumab showed little to no effect compared to DMSO in the case of IL-6 levels suggesting that blocking IL-6 signaling does not dramatically impact its secretion (Figure 2C). In addition, we assessed microtissue viability by measuring the diameter of each microtissue compared to the untreated wells and the DMSO-treated groups. We observed no significant decrease in SM viability for most compounds, including CTRLs. Compounds affecting microtissue or microglia spheroid viability were excluded from further analysis (Figure S2B). We quantified secreted IL-6 and IL-8 percent (%) reductions relative to DMSO-treated CTRLs as the primary screening readout. Hits were defined as compounds reducing IL-6 and/or IL-8 beyond set thresholds.

In C9-ALS SMs, we identified 24 IL-8 hits (20%–70% decrease) and 23 IL-6 hits (20%–60% decrease) (Figure 2D). In C9-ALS microglia spheroids, 45 compounds reduced IL-8 (20%–90%) and 66 reduced IL-6 (15%–60%) (Figure 2E). The top 15 hits per group were selected based on potency of IL-6 and IL-8 inhibition (Figures 2D and 2E). In SMs, telmisartan ranked the highest, reducing IL-8 by 65% and IL-6 by 50% (Figure 2D). In microglia spheroids, flutamide and delavirdine mesylate were the top IL-8 and IL-6 inhibitors, respectively (Figure 2E). Hit thresholds were determined using the negative CTRL (tocilizumab-treated) versus DMSO (Figure 2F). Notably, sartans, the top hits in C9-ALS SMs, were also effective in microglia spheroids, suggesting their ability to reduce IL-6 and IL-8 in both platforms (Figure 2G). Further details are in the methods section. To assess screen robustness, we performed a Z-prime factor analysis, measuring signal separation between the positive and vehicle CTRLs. Results were plotted as probability distributions against log-transformed assay signals, visualized as bell curves (Figures S2C and S2D).

Confirmation of telmisartan as a key hit in decreasing neuroinflammation in C9-ALS SMs

Because sartans were the top hits reducing IL-6 and IL-8 in both microglia and C9-ALS SMs, we validated their effects using telmisartan, valsartan, and azilsartan in a secondary screen. Validation was conducted on C9-ALS SMs derived from different hiPSC lines and their isogenic CTRLs, generated via CRISPR-Cas9 homology-directed repair (HDR). Details, including the gene editing strategy using CRISPR-Cas9 (HDR) to generate these lines, are depicted in Figure 3A. Following the original screening design, SMs were treated with 5 μM sartans on D2 and cultured for

TocriScreen library was applied at 5 μM and plates were incubated at 37°C for 72 h. Subsequently, the supernatants were collected from each well and sent for multiplex cytokine profiling, specific for IL-6 and IL-8.

(C) IL-6 and IL-8 concentrations in C9-ALS SMs and microglia spheroids treated with negative control (tocilizumab, 1 μM), positive control (LPS, 1 μg/mL), and vehicle control (DMSO, 0.01%) demonstrate significantly lower IL-6 and IL-8 concentrations in vehicle control-treated groups relative to LPS-treated groups in both the SM and microglia spheroid groups. The percent decrease in IL-6 and IL-8 in tocilizumab-treated groups against vehicle control-treated groups was used as a cutoff threshold for determining hits (referred to as “hit threshold”). These thresholds are detailed in Figure 2F.

(D) Screen results, shown as percent decrease against the vehicle control, highlight compounds that lowered IL-8 and IL-6 in C9-ALS SM supernatants. The red dashed line indicates the hit threshold for each condition. Red labels denote top hits, identified by their abbreviations (full names in Figure S2A), while black labels indicate toxic compounds removed from further analysis. Bar charts rank the top 15 hits by potency of inhibition, defined as the percent reduction in IL-6 or IL-8 relative to the vehicle control. Data were generated from a single differentiation (technical replicate).

(E) Screen results show the percent decrease in IL-8 and IL-6 concentrations relative to vehicle control, highlighting hits in C9-ALS microglia spheroid supernatants. This panel specifically represents findings for microglia spheroids. See (D) for details on thresholds, labels, and ranking criteria.

(F) Hit thresholds are depicted as % decrease of IL-6 or IL-8 with 1 μM tocilizumab treatment against DMSO in either SMs or microglia spheroids.

(G) Venn diagrams depicting shared ARB hits among each group and those that were mutually exclusive. Telmisartan, Valsartan, and Azilsartan are identified as the hits that are shared between spinal microtissues and microglia spheroids.

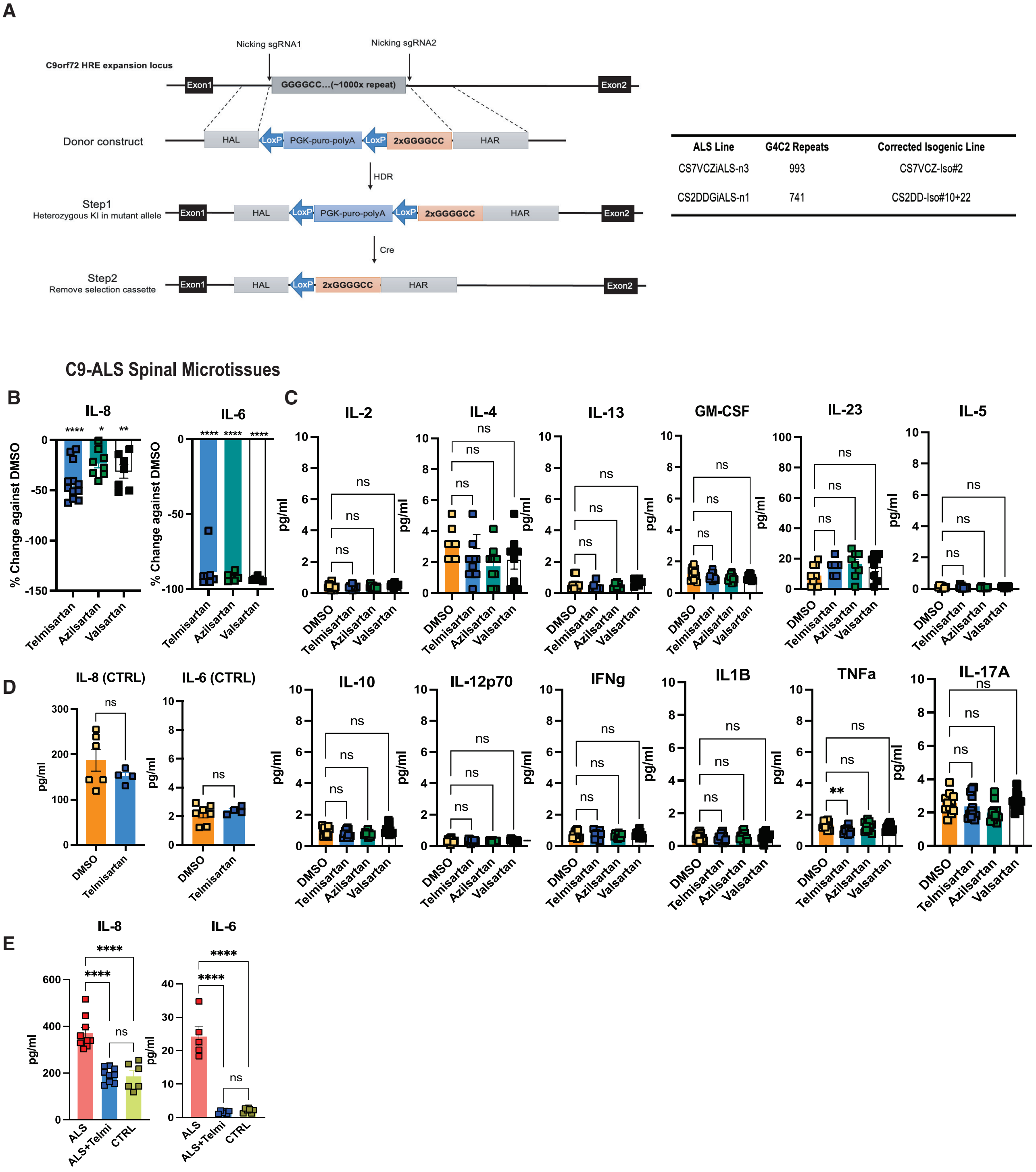

Figure 3. Validation of ARBs on neuroinflammation in C9-ALS spinal microtissues

(A) Gene correction of C9orf72 ALS patient iPSC lines. The CS7VCZiALS and CS2DDGiALS iPSC lines, carrying heterozygous G4C2 repeats of 993 and 741 sequences, respectively, were corrected using CRISPR-Cas9-mediated HDR.

(B) Validation screen was performed in C9-ALS SMs, which were treated with Telmisartan, Valsartan, and Azilsartan (all compounds at 5 μM) following the same paradigm as the HTS. Cytokine analysis shows the percent decrease in IL-6 and IL-8 concentrations relative to DMSO

(legend continued on next page)

72 h before supernatant analysis. Telmisartan, azilsartan, and valsartan significantly reduced IL-6 and IL-8 in C9-ALS SMs (Figures 3B and 3C). However, telmisartan had no significant effect on IL-6, IL-8, or other cytokines in isogenic CTRL SMs (Figure 3D), suggesting its effect is specific to C9-ALS (Figure S3A). Secretome analysis confirmed IL-8 (CXCL8) as a top upregulated protein in C9-ALS SMs (Figure S3B) and the most significantly reduced following telmisartan treatment, further supporting our findings (Figure S3C).

Telmisartan enhances survival of spinal MNs in C9-ALS co-cultures and tricultures

To assess telmisartan's effect on spinal MN survival under C9-ALS conditions, we generated spinal MNs from a GPI: H2B-tdTomato-tagged hESC line (Guttikonda et al., 2021) and cultured them alone or with C9-ALS or CTRL glia (microglia and/or spinal astrocytes) under mono-, co-, and triculture conditions. Cells were treated with DMSO (CTRL) or telmisartan (5 μM) on D2, and live-cell imaging was performed every 4 h for 14 days (Figure 4A). Td-Tomato counts showed no significant difference in MN survival between untreated and telmisartan-treated groups in monoculture (Figure 4B) or when co-cultured with CTRL microglia (Figure 4C). However, telmisartan significantly improved MN survival in co-culture with C9-ALS microglia (Figure 4D). In the triculture model, telmisartan had no effect with wild-type glia (Figure 4E) but significantly increased MN survival in the C9-ALS glia-based setting (Figure 4F). Analysis of "motor neuron death" (net Td-Tomato change between the first and last time points) showed that MNs co-cultured with C9-ALS microglia exhibited significantly higher death counts compared to those co-cultured with CTRL microglia, and telmisartan treatment reduced MN death in the C9-ALS co-culture to levels similar to CTRL microglia (Figure 4G). No significant difference was observed between untreated and telmisartan-treated CTRL microglia co-cultures. Similarly, MNs in C9-ALS tricultures, which included both microglia and astrocytes, showed significantly higher death counts compared to those co-cultured with CTRL microglia (Figure 4G). Finally, supernatant collected after 14 days of culture was analyzed for cytokine levels, revealing that IL-6 and IL-8 levels were significantly elevated in C9-ALS co-cultures and tricultures compared to CTRLs (Figure 4H). Telmisartan treatment significantly reduced IL-6 and IL-8 levels in both C9-ALS co-cultures and tricultures, restoring them to baseline CTRL levels (Figure 4H). These findings suggest that telmisartan has a neuroprotective effect in C9-ALS models derived from patient induced pluripotent stem cells (iPSCs) by enhancing MN survival in a non-cell-autonomous manner and reducing C9-microglia-mediated pro-inflammatory cytokine levels.

Telmisartan shows time-dependent effect on C9-ALS SM cell ratios and rescues MN proportion

As the previous survival studies were performed in 2D co-culture and tri-culture models (Figure 4), we explored whether we could assess the role of telmisartan in our 3D SM platform using whole-mount staining to quantify cell proportions over a 14-day period. SMs were generated and treated with either 0.01% DMSO or 5 μM telmisartan using the same experimental paradigm depicted in Figure 2B. SMs were collected and fixed on days 5 (D5), 7 (D7), and 14 (D14) post-generation and stained using whole-mount IF analysis, as described in the methods section. Antibodies against ISL-1 (spinal MNs), IBA-1 (microglia), and glial fibrillary acid protein (GFAP; spinal astrocytes) were used to determine the proportions of the three cell types over time in culture. The bar graphs (Figures S4A–S4D) depict the proportions of IBA-1+ microglia, GFAP+ astrocytes, and ISL-1+ MNs on D5, D7, and D14. Interestingly, the percentage of ISL-1+ MNs was significantly lower in C9-ALS compared to CTRL SMs on D14 (Figure S4F). With telmisartan treatment (C9-ALS + Telmi), ISL-1+ MN proportions on D5 and D7 showed

(vehicle control). One-way ANOVA with Šídák's multiple comparisons test $*p < 0.05$, $**p < 0.01$, $****p < 0.001$. $N = 3$ replicates, 3 independent differentiations from 2 different lines for each. Data are represented as mean ± SEM.

(C) Bar graphs depict the effects of telmisartan, azilsartan, and valsartan on IL-2, IL-4, IL-5, IL-12, IL-13, GM-CSF, IL-10, IL-12p70, interferon (IFN)γ, IL-1β, TNF-α, and IL-17a concentrations. Results show no significant differences in the cytokine concentrations in the C9-ALS SM supernatants after 72 h of treatment, except for a significant decrease in TNF-α with 5 μM telmisartan treatment. One-way ANOVA with Šídák's multiple comparisons test, $*p < 0.05$, $**p < 0.01$, $****p < 0.001$. $N = 3$ replicates, 3 independent differentiations from 2 different lines. Data are represented as mean ± SEM.

(D) Telmisartan was applied to healthy control SMs at 5 μM following the same paradigm. Bar graphs show no significant difference in IL-6 and IL-8 concentrations between DMSO and telmisartan treatment. Student's t test with multiple comparisons. ns, not significant. $N = 3$ replicates, 2 independent differentiations from two isogenic control lines. Data are represented as mean ± SEM.

(E) Bar graphs depicting IL-6 and IL-8 concentrations from C9-ALS, control, and C9-ALS SMs treated with 5 μM telmisartan. Results show that telmisartan treatment lowers IL-8 and IL-6 concentrations in C9-ALS SMs to levels observed in control SMs. One-way ANOVA with Šídák's multiple comparisons test, $*p < 0.05$, $**p < 0.01$, $****p < 0.001$. $N = 3$ replicates, 3 independent differentiations from 4 different lines for all the panels. Data are represented as mean ± SEM.

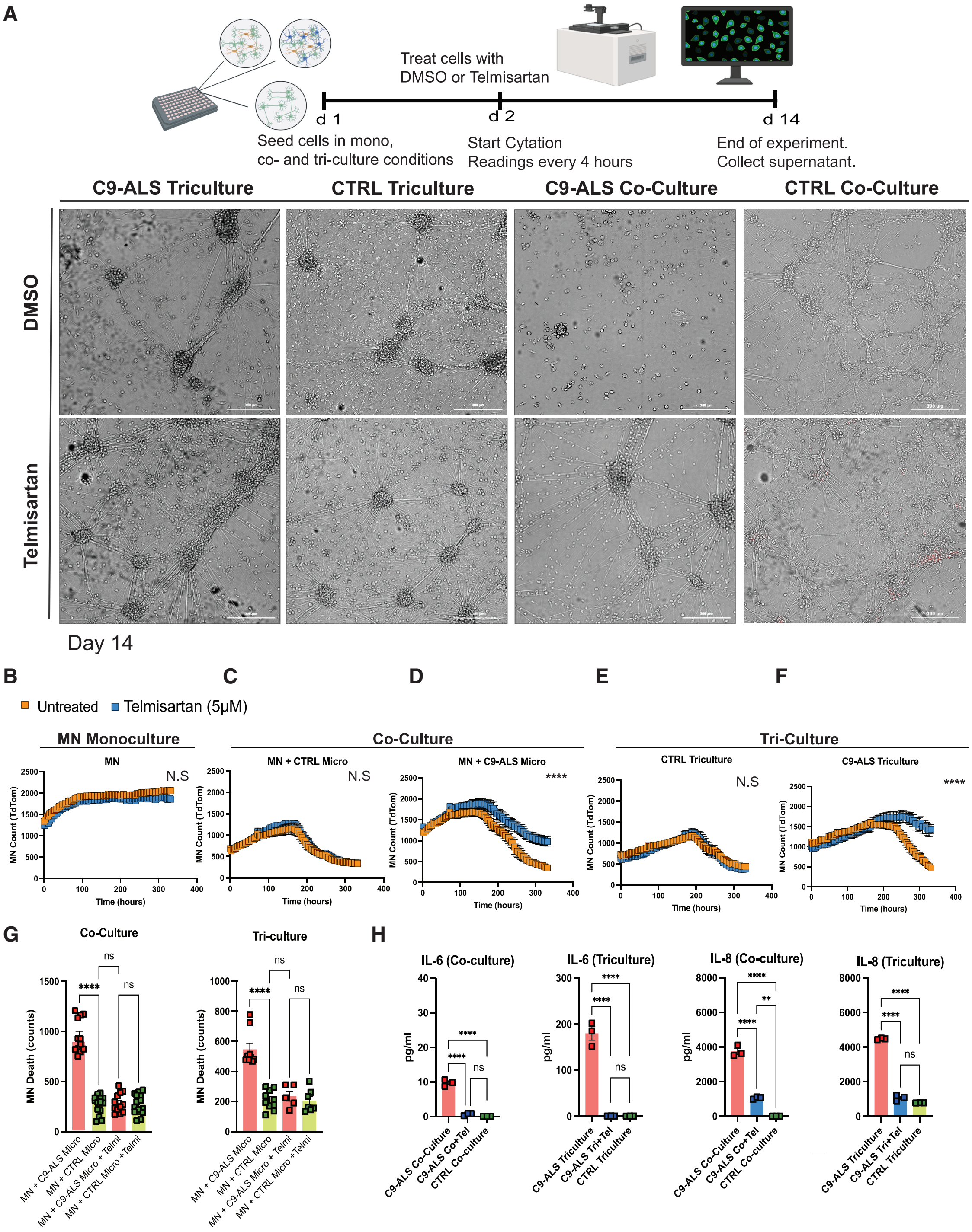

Figure 4. Telmisartan enhances motor neuron survival in C9-ALS tri- and co-culture models

(A) Experimental timeline and setup for assessing the effects of DMSO and telmisartan on cells cultured under 2D mono-, co-, and tri-culture conditions. All MN were wild-type and tagged (GPI:H2B-TdTom). On day 0 (d0), cells were seeded into a 96-well plate in the specified culture conditions. On day 1 (d1), cells were treated with either DMSO or telmisartan. Starting from day 2 (d2), live-cell imaging was initiated using a Cytation instrument, with readings taken every 4 h over a 14-day period to monitor cellular responses. Bottom panel depicts bright-field images at 336 h. Td-tomato counts were recorded every 4 h.

(legend continued on next page)

a slight increase compared to their DMSO-treated counterparts (Figures S4A and S4C), with the most significant difference observed on D14, where the ISL-1+ MN population was significantly higher with telmisartan treatment compared to the DMSO group on D14 (Figures S4C and S4E). In the CTRL (DMSO) group, ISL-1-positive cells remained similar across all the time points, regardless of telmisartan treatment (Figures S4B and S4D). Collectively, these results support our findings that MN populations decline over time in C9-ALS compared to CTRL SMs and that telmisartan can mitigate this effect.

DISCUSSION

Our SM model provides a controlled *in vitro* platform to study cell-autonomous and non-cell-autonomous interactions while enabling scalable high-throughput screening with just 7,500 cells per microtissue. Its novelty lies in mimicking the dynamic interplay between MNs and glia at physiological cell ratios under highly defined conditions, offering a more accurate representation of disease states. By deriving all cell types from a single hiPSC source, it ensures a physiologically relevant cellular environment, preserving intricate cellular interactions often missed in simpler models. Our findings, particularly the identification of sartans as IL-6 and IL-8 modulators with neuroprotective potential, highlight the model's value in drug discovery. IL-6 and IL-8 have emerged as neuroinflammatory signatures in the context of ALS, with some studies highlighting their elevated levels in human ALS cerebrospinal fluid (CSF) and plasma (Mennini et al., 2009; Moreau et al., 2005; Wosiski-Kuhn et al., 2021). These cytokines are thought to play roles in mediating the inflammatory response within the CNS, contributing to the progression of neurodegeneration (Zhang et al., 2023). IL-6 is known to promote the survival and differentiation of neurons under normal conditions, but, in the diseased state, its chronic elevation may lead to detrimental effects, including the exacerbation of neuroinflammation and neuronal loss (Ehrhart et al., 2015; Tortelli et al., 2020). Similarly, IL-8 has been implicated in the inflammatory milieu associated with ALS (Ehrhart et al., 2015; Femiano et al., 2024; Masrori et al., 2022). In our SM model, we identified these cytokines as neuroinflammatory signatures specific to C9-ALS not observed in CTRL SMs, highlighting their potential role in the disease pathology. By using our model, we screened 190 compounds to identify modulators of IL-6 and IL-8 and identified multiple sartans as hits. We also demonstrated a correlation between neuroprotection and the modulation of IL-6 and IL-8 in C9-ALS.

(B) The effects of telmisartan (5 μM) on motor neuron (MN) counts over time were assessed across various experimental conditions, comparing untreated (DMSO, orange) and telmisartan-treated (blue) groups. Td-Tomato counts were recorded every 4 h for 336 h in the following settings: MN alone, MN + CTRL microglia, MN + C9-ALS microglia, C9-ALS triculture (MN + C9-ALS microglia + C9-ALS astrocytes), and CTRL triculture (MN + CTRL microglia + CTRL astrocytes). CTRL refers to cells derived from either (1) healthy control donors age- and gender-matched to C9-ALS patient lines or (2) isogenic controls generated by correcting the C9orf72 repeat expansion in C9-ALS patient lines. Scatterplots illustrate differences in Td-Tomato counts over 14 days between telmisartan-treated and untreated groups across these conditions.
(C) When MNs are co-cultured with control microglia, there is also no significant difference (NS) in MN counts between untreated and telmisartan-treated groups.
(D) MNs co-cultured with C9-ALS microglia, telmisartan treatment results in higher MN counts compared to untreated groups, suggesting a protective effect of telmisartan on MNs in the presence of C9-ALS microglia.
(E) In the control triculture model, telmisartan treatment does not significantly affect MN survival, as indicated by the no significant difference (NS) in MN counts over time between untreated and telmisartan-treated groups.
(F) Telmisartan treatment in the C9-ALS triculture group results in significantly higher motor neuron (MN) counts compared to the untreated group.
(G) MNs co-cultured with C9-ALS microglia exhibit significantly higher death counts compared to MNs co-cultured with CTRL microglia ($p < 0.001$). Treatment with telmisartan significantly reduces MN death in the C9-ALS co-culture to levels similar to those seen in MNs co-cultured with CTRL microglia. There is no significant difference (NS) in MN death counts between untreated and telmisartan-treated CTRL microglia co-cultures. Similarly, MNs in the presence of C9-ALS microglia+C9-ALS astrocytes show significantly higher death counts compared to MNs with CTRL microglia ($p < 0.001$). Telmisartan treatment again significantly reduces MN death in the C9-ALS triculture to levels comparable to those in the CTRL triculture, with no significant difference (NS) observed between untreated and telmisartan-treated CTRL tricultures.
(H) Supernatants were collected on day 14 and sent for multiplex cytokine analysis. Results demonstrate that, in C9-ALS co-cultures and tricultures, IL-6 and IL-8 levels are significantly elevated compared to CTRLs (****$p < 0.001$). Telmisartan treatment significantly reduces IL-6 and IL-8 levels in both C9-ALS co-cultures and tricultures (****$p < 0.001$), reducing the levels to baseline CTRL levels. One-way ANOVA with Šídák's multiple comparisons test, *$p < 0.05$, **$p < 0.01$, ****$p < 0.001$. Data were generated using two C9-ALS patient lines and their isogenic controls. $N = 3$ replicates each. Data are represented as mean ± SEM.

In contrast to the elevated IL-6 and IL-8 levels in C9-ALS SMs and microglia spheroids, we found low levels in C9-ALS astrocytes and MNs, highlighting microglia as key mediators of the disease-related neuroinflammatory response. Beyond secreting IL-6 and IL-8, C9-ALS microglia were neurotoxic, even in the absence of external stimuli, unlike previous studies using LPS or excitotoxic challenges (Banerjee et al., 2023; Vahsen et al., 2022, 2023). This suggests that C9-ALS is not solely MN autonomous but actively driven by microglia, potentially via IL-6 and IL-8 pathways. Our SM model confirmed this role, showing significant MN loss in the C9-ALS group, which was rescued by telmisartan treatment. These findings enable further mechanistic studies on neuroinflammation and the identification of key drivers of MN toxicity in C9-ALS microglia.

Our data implicate the renin-angiotensin-aldosterone system (RAAS) in C9-ALS pathogenesis. RAAS, primarily known for regulating blood pressure (Nakagawa et al., 2020), is also active in the CNS, where angiotensin II (Ang II) and its receptors contribute to neuroinflammation, oxidative stress, and blood-brain barrier disruption (Huang and Zhang, 2024). AT1R activation exacerbates microglial activation and pro-inflammatory cytokine release (Kim et al., 2022), while RAAS inhibition via ARBs or angiotensin converting enzyme (ACE) inhibitors may reduce neuroinflammation (Benicky et al., 2011; Joglar et al., 2009). Sartans, prescribed for hypertension and heart failure, are not currently used for inflammatory conditions, though some studies suggest anti-inflammatory effects in cardiovascular disease and diabetes (Chen et al., 2018; Klinghammer et al., 2013). A meta-analysis also reported that telmisartan reduces IL-6 and tumor necrosis factor alpha (TNF-α), supporting its potential as an anti-inflammatory agent (Takagi et al., 2013). Beyond systemic RAAS, brain-specific RAAS (b-RAS) has been implicated in local immune modulation (Sigmund and Grobe, 2020) and microglial polarization via NADPH oxidase activation (Labandeira-Garcia et al., 2017). AT1R inhibition has shown functional benefits in preclinical models of stroke (Wanderer et al., 2020), Alzheimer's (Mogi and Horiuchi, 2009), and Parkinson's disease (Labandeira et al., 2022). Telmisartan (Micardis) is a potent, blood-brain barrier (BBB)-penetrant ARB (Noda et al., 2012), with cohort studies suggesting an inverse correlation between RAS-acting drugs and neurodegenerative diseases, including Alzheimer's disease (Ho et al., 2021; Lee et al., 2023). Notably, ACE inhibitors have been linked to a lower ALS incidence (Lin et al., 2015), but no studies have explored ARBs in C9-ALS. Here, we demonstrate sartans, particularly telmisartan, as potential C9-ALS therapeutics by reducing neuroinflammation and MN death.

Our findings suggest that IL-6 and IL-8 are key neuroinflammatory cytokines in C9-ALS and that microglia play an active role in its pathology. These results support targeting IL-6 and IL-8 pathways for therapy, including testing telmisartan in C9-ALS progression. However, IL-6 or IL-8 alone did not replicate MN death observed with C9-ALS microglia or tricultures, suggesting that additional toxic factors contribute. Telmisartan may offer broader benefits beyond direct IL-6/IL-8 inhibition. For translational studies, determining whether peripheral telmisartan administration can achieve therapeutic CNS levels comparable to our 5 μM *in vitro* concentration is critical. Its high plasma protein binding (∼99.5%) limits free drug availability, but preclinical studies in monkeys (1 mg/kg IV) show BBB penetration and AT1R inhibition in the brain (Noda et al., 2012). Translating this to humans may require intrathecal delivery for optimal neuroprotection. The SM platform, leveraging hiPSC technology, integrates the spinal cord neuroinflammatory axis while maintaining scalability for high-throughput screening. It is cost effective, easy to fabricate, and adaptable for drug discovery. Beyond ALS, SMs could advance personalized medicine by predicting drug efficacy, optimizing clinical trial design, and guiding patient stratification. Realizing the full potential of this platform could accelerate ALS research and translate promising findings from bench to bedside.

Limitations of the study

Our study demonstrates a robust effect of multiple sartans on neuroinflammation in *C9orf72* SMs and in microglia and shows that telmisartan protects spinal motoneurons from *C9orf72*--microglia-mediated death *in vitro*. However, the study has several limitations. First, although our screening was conducted in 3D microtissues, the pathophysiological analysis in 3D microtissues remains limited. Second, while we utilized hiPSCs from two independent *C9orf72* backgrounds, two age-matched CTRLs, and two gene-corrected isogenic clones corrected to physiological repeat length, future studies are needed to test larger panels of *C9orf72* and sporadic hiPSC lines. Such studies may address whether our findings are broadly applicable to ALS or specific to *C9orf72*. Third, our focus was on the impact of the *C9orf72* mutation in triculture models and microglia-MN co-cultures. However, we have not explored a specific role of *C9orf72* astrocytes using co-cultures of astrocytes and MNs, and the specific factors driving the increase in inflammatory cytokines in C9orf72 microglia remain unclear. Furthermore, studies are needed to address whether the anti-inflammatory and neuroprotective effects are directly mediated by the Ang II type 1 receptor. Finally, this manuscript focused on validating sartans, and other candidate hits from our screen need further validation but may represent interesting targets for future research.

METHODS

hiPSC cell culture

Experiments using human pluripotent stem cells were reviewed by the Tri-institutional ESCRO committee. Human iPSCs were maintained in Essential 8 medium on Vitronectin-coated plates, as previously described (Tchieu et al., 2017) The working cell banks (WCBs) were expanded from the master cell banks obtained from the Answer ALS repository. hiPSC stocks were passaged at a 1:6 ratio, cryopreserved using Stem Cell Banker as the freezing medium, and subsequently thawed into Essential 8 medium supplemented with 10 μM ROCK inhibitor (Y-27632). Cells were maintained in this medium for 24 h post-thawing. All WCB stocks were used between passages 20–30, and differentiations were initiated two passages following thawing. For additional information see supplemental methods.

Generation and dissociation of 3D, spinal MN organoids from hiPSCs in 3D

Spinal MNs were generated in 3D as organoids. For dissociation, 10 organoids were collected and subjected to a papain dissociation technique following manufacturer instructions. For additional information on MN generation and dissociation see supplemental methods.

Generation of spinal astrocytes and microglia from hiPSCs

The spinal astrocyte protocol was adjusted from Tchieu et al. (2019) to generate spinal cord-specific astrocytes. Microglial differentiation followed Guttikonda et al. (2021). For additional information on those two protocols see supplemental methods.

Generation of SMs

SMs were generated similarly to cardiac microtissues (Giacomelli et al., 2020) but by mixing microglia, spinal MNs, and spinal astrocytes at a ratio of 1:3:1 in SM media—Neurobasal media containing brain-derived neurotrophic factor (BDNF; 0.02 μg/mL), glial cell line-derived neurotrophic factor (GDNF; 0.02 μg/mL), and IL-34 (100 ng/mL). Briefly, microglia and astrocyte stocks were thawed and allowed to recover in serum-free medium for one week, whereas day 20–30 spinal MN organoids were dissociated using papain dissociation (Worthington Biochemical Corporation, Cat #: LK003150) for 1 h and 10 min on an orbital shaker and counted by trypan blue. Meanwhile, day 30 microglia were dissociated into a single-cell suspension using Accutase for 10 min at 37° C and counted by trypan blue. Simultaneously, day 50 spinal astrocytes were dissociated with trypsin at 37° C, and cells were counted by trypan blue. Spinal MNs, microglia, and spinal astrocytes were mixed to generate a total of 7,500 cells comprising 4,500 spinal MNs, 1,500 spinal astrocytes, and 1,500 microglia per microtissue and seeded in 100 μL of microtissue media per well in a low-attachment 96-well V-bottom plate (S-Bio PrimeSurface 3D culture: ultra-low-attachment plates, Cat #MS-9096VZ). Plates were centrifuged at 1,090 rpm for 10 min at room temperature (RT) and incubated under standard cell culture conditions (37°C, 5% CO_2).

High-throughput screen

SM and microglia spheroids were generated in 100 μL of media as described earlier. After 24 h, the wells were treated with the TocriScreen FDA-Approved Drugs Library (Tocris, Cat. #7200) using a semi-automated system. The screening was conducted in single wells for the compounds, with triplicates for the positive, negative, and vehicle CTRLs. The final concentration of each compound was 5 μM. Plates were incubated at 37°C with 5% CO_2 for 72 h. After incubation, supernatants were carefully collected without disturbing the cells using a robotic system. The supernatants were immediately frozen at −80°C for subsequent analysis. Cytokine levels (IL-6 and IL-8) were measured using Eve Technologies' Human High Sensitivity Custom 2-Plex Cytokine Assay. Quantitative analysis was performed in collaboration with the MSKCC Gene Editing & Screening Core.

Whole-mount staining of microtissues

To stain SMs in 3D, we modified protocols described previously (Dekkers et al., 2019; Giacomelli et al., 2020). For additional information see supplemental methods.

Immunostaining of monolayer cultures

Cells were fixed in 4% paraformaldehyde in PBS for 20 min, permeabilized in 0.1% Triton X-100 in PBS for 15, and blocked for 30 min in PBS with 1% bovine serum albumin (BSA). Primary antibody incubation was performed overnight at 4 °C at the specified dilutions in 1% BSA-PBS. Following three washes with PBS, cells were incubated with fluorescently conjugated secondary antibodies (2 μg mL^{-1}) and DAPI (1 μg μL^{-1}) for 45 min at RT. Antibody details can be found in the supplemental information.

Cytokine arrays

This study used Luminex xMAP technology for multiplexed quantification of 14 human cytokines, chemokines, and growth factors. The multiplexing analysis was performed using the Luminex 200 system (Luminex, Austin, TX, USA) by Eve Technologies (Calgary, Alberta). For additional information see supplemental methods.

Protein digestion

Samples were loaded into Amicon Ultra centrifugal filters (3 kDa molecular weight cut off), concentrated to a final volume of 100 μL, and transferred to a new Eppendorf tube. The filters were then rinsed with 4 M urea and 50 mM EPPS pH 8.5, and the rinse was transferred to the samples tube. After protein quantification using the Pierce bicinchoninic acid assay (Thermo Fisher Scientific), the proteins were reduced with tris (2-carboxyethyl) phosphine to a final concentration of 5 mM for 20 min at RT. Free cysteine residues were alkylated with iodoacetamide at a final concentration of 10 mM for 20 min in the dark at RT. The samples were cleaned using SP3 Sera-Mag carboxylate SpeedBeads beads (Cytiva, cat#: 65152105050250; 45152105050250, 50 μg/μL) according to the manufactures' instructions. The beads were then resuspended in 50 μL of 50 mM triethylammonium bicarbonate, and the samples were digested with Lys-C (Wako) at a 1:100 enzyme-to-protein ratio and trypsin (Promega) at a 1:100 enzyme-to-protein ratio at 37°C for overnight incubation with shaking (1200 rpm) on a thermomixer (Thermo Fisher Scientific). After digestion, the samples were acidified with 2.65 μL of 99% formic acid (FA) at RT for 5 min with shaking (1,200 rpm), followed by addition of 1.2 mL of 100% acetonitrile (ACN) for 10 min at RT with shaking (1,200 rpm). The samples were washed three times with 100% ACN and resuspended in 50 μL of 2% DMSO at 37°C for 30 min with shaking (1,200 rpm). The eluted peptides were dried under vacuum, reconstituted in water with 0.1% FA, and sonicated in a water bath sonicator. Peptide yield was quantified using a NanoDrop (Thermo Fisher Scientific).

Mass spectrometry analyses

Peptides were separated on a 25 cm column with a 75 μm diameter and 1.7 μm particle size, composed of C18 stationary phase (IonOpticks Aurora 3 1801220). The separation was carried out using a 25-min gradient: from 3.2% to 9.6% B over 2 min at a flow rate of 400 nL/min and then to 18% B over 12 min at 200 nL/min, to 32% B over 19.5 min at 200 nL/min, and finally to 90% B over 5 min at 300 nL/min. The mobile phase A was 0.1% FA in high-performance liquid chromatography-grade water, and mobile phase B ACN with 0.1% FA. The separation was performed using a Vanquish Neo system (Thermo Fisher Scientific). Mass spectrometry (MS) data were acquired on an Orbitrap Astral mass spectrometer (Thermo Fisher Scientific) in a data-independent acquisition mode, with a normalized collision energy of 25%. MS1 spectra were acquired in the Orbitrap at a resolution of 240 K, with a normalized automated gain control target 200%, a custom maximum injection time, and a scan range of 380–980 m/z. MS/MS spectra were acquired in the Astral analyzer with a 2 m/z isolation window, a scan range of 150–2,000 m/z, a precursor mass range of 380–980 m/z, and a loop CTRL time of 0.6 s.

Analyses of MS data

Raw data files were processed using Spectronaut version 18.5 (Biognosys) and searched with the PULSAR search engine against a *Homo sapiens* UniProt protein database downloaded on 2024/05/28 (226,232 entries). Cysteine carbamidomethylation was specified as fixed modifications, while methionine oxidation, acetylation of the protein N-terminus, and deamidation (NQ) were set as variable modification. A maximum of two trypsin missed cleavages were allowed. Searches utilized a reversed sequence decoy strategy to CTRL peptide false discovery rate (FDR), with a 1% FDR threshold set for identification. An unpaired t test was used to calculate p values in differential analysis. The volcano plot was generated based on log2 fold change (log2FC) and q value (multiple testing corrected p value using the Benjamini-Hochberg method). A q value of ≤ 0.05 was considered the statistically significant cutoff.

Live imaging with BioTek Cytation 5 cell imaging multimode reader

We utilized the BioTek Cytation 5 cell imaging multimode reader, which integrates automated microscopy and multimode detection. For additional information see supplemental methods.

Flow cytometry

Flow cytometry analysis of CD14 expression was performed using a PE-conjugated anti-CD14 antibody (clone M5E2, STEMCELL Technologies), after incubation with FcR blocking reagent (Miltenyi Biotec). ISL-1 and IBA-1 expression were detected following fixation and permeabilization of cells using BD Cytofix/Cytoperm solution (BD Pharmingen). Cells were marked with Zombie Violet Viability (BioLegend). After incubation with FcR blocking reagent (Miltenyi Biotec), cells were stained with anti-IBA (clone 019-19741, Wako) and anti-ISL-1 (Clone 39.4D5-s, Developmental Studies Hybridoma Bank) primary antibodies, followed by staining with Alexa Fluor 488- and Alexa Fluor 555-conjugated secondary antibodies (Thermo Fisher Scientific). Flow cytometry was performed using a BD Biosciences LSR Fortessa flow cytometer with Diva software. Data were analyzed using FlowJo (BD Biosciences LLC).

Gene correction and validation for C9orf72 ALS iPSC line

The C9-ALS patient hiPSC lines were obtained from Answer ALS (Baxi et al., 2022): the CS7VCZiALS iPSC lines carries a

heterozygous 993 G4C2 repeating sequences, and the CS2DDGiALS carries a heterozygous 741 G4C2 repeating sequences in the intron 1 of *C9orf72* gene. The gene correction was performed using CRISPR-Cas9-based HDR. For additional information see supplemental methods.

3D image processing and analysis

Image processing and analysis were conducted using Fiji in conjunction with custom computational routines implemented in Python and Julia. A standardized pipeline was applied to each confocal image, encompassing kernel size determination, image processing, and 3D object classification and analysis as described in the supplemental methods.

Statistical analysis

Results are shown as mean ± SEM. Mean values were compared between CTRL cells and cells from the patients in one-way ANOVA with Sidak's multiple comparisons tests. One-way ANOVA, two-way ANOVA, Student's t test, for paired or unpaired measurements was applied where indicated and appropriate. Statistical analysis was performed using GraphPad Prism 9 (v.9.2.0). Statistical significance is denoted as follows: NS, not significant; $p > 0.05$; *$p < 0.05$; **$p < 0.01$; ***$p < 0.001$; ****$p < 0.0001$. Results with p values <0.05 were considered statistically significant.

RESOURCE AVAILABILITY

Lead contact

Requests for further information, resources, and reagents should be directed to the lead contact, Lorenz Studer, studerl@mskcc.org.

Materials availability

The unique cell lines generated from this study are available from the lead contact. The unmodified C9ORF72 patient and CTRL hiPSC lines were obtained from the Answer ALS via the Cedars Sinai Biomanufacturing Center.

Data and code availability

The mass spectrometry proteomics data generated in this study have been deposited in the ProteomeXchange Consortium via the PRIDE partner repository with the dataset identifier PXD057067.

ACKNOWLEDGMENTS

We are grateful to the members of the Studer lab for helpful discussions and support of this study. We thank the MSKCC Gene Editing and Screening Core for their help in designing and executing the high-throughput screen (HTS). Furthermore, we thank Drs. Mary Baylies, Li Gan, and Shuibing Chen for insightful discussions. Moreover, we acknowledge BioRender.com for providing the tools to create illustrations used in this publication. We also thank Dr. Ryan Walsh for generating the GPI:H2B-Td-tomato-tagged ESCs. Additionally, we acknowledge the use of ALS patient-derived iPSC lines from the Answer ALS project, led by Cedars-Sinai Medical. We extend our deepest gratitude to the patients, their families, and the healthy control donors for their invaluable contributions to this research. The work was funded by grant AL200169 - W81XWH2110140 from the Department of Defense and a Project ALS award to L.S. Additional support was provided by grants from the National Institutes of Health (R21NS116545, R01MH135403, core grant P30CA08748), and the JPB foundation/Freedom together Foundation to L.S. B.S. was supported by The Dompé Rita Levi Montalcini Fellowship, B.F.V. was supported by the Motor Neurone Disease Association (MND Association; project grant Talbot/Apr22/889-791) and a Boehringer Ingelheim Fonds travel fellowship, L.W. by Charles Revson fellowship, and E.G. by Rubicon fellowship (2020/30766/ZONMW).

AUTHOR CONTRIBUTIONS

B.S. designed, performed, and interpreted most of the experiments; analyzed the data; generated the figures; and wrote the manuscript. B.F.V. performed some of the SM generation and immunofluorescent staining experiments and wrote the manuscript. L.W. performed and analyzed flow cytometry experiments. N.X. performed the spinal MN death assay and helped with the data analysis. E.L.C. and J.J. developed the two-dimensional spinal MN protocol from which the 3D spinal MN protocol is based upon. A.Z. generated the isogenic C9-ALS lines used in the screen validation. M.L.-T. and D.M.G. performed and analyzed the SM cell proportion quantifications. L.T. processed all samples for proteomic analyses. M.M. and Z.L. performed, analyzed, and interpreted the proteomics data analyses. T.Z. supervised and generated the isogenic lines used in the screen validation. E.G. conceived the idea; designed the study; developed and optimized the 3D spinal MN protocol, spinal astrocyte protocol, and SM model; and wrote the manuscript. L.S. conceived the idea, designed the study, generated funding, and wrote the manuscript.

DECLARATION OF INTERESTS

L.S. is a scientific cofounder and paid consultant of BlueRock Therapeutics Inc. and a scientific cofounder of DaCapo Brainscience.

SUPPLEMENTAL INFORMATION

Supplemental information can be found online at https://doi.org/10.1016/j.stemcr.2025.102535.

Received: February 26, 2025
Revised: May 19, 2025
Accepted: May 20, 2025
Published: June 19, 2025

REFERENCES

Balendra, R., and Isaacs, A.M. (2018). C9orf72-mediated ALS and FTD: multiple pathways to disease. Nat. Rev. Neurol. *14*, 544–558. https://doi.org/10.1038/s41582-018-0047-2.

Banerjee, P., Mehta, A.R., Nirujogi, R.S., Cooper, J., James, O.G., Nanda, J., Longden, J., Burr, K., McDade, K., Salzinger, A., et al. (2023). Cell-autonomous immune dysfunction driven by

disrupted autophagy in C9orf72-ALS iPSC-derived microglia contributes to neurodegeneration. Sci. Adv. *9*, eabq0651. https://doi.org/10.1126/sciadv.abq0651.

Baxi, E.G., Thompson, T., Li, J., Kaye, J.A., Lim, R.G., Wu, J., Ramamoorthy, D., Lima, L., Vaibhav, V., Matlock, A., et al. (2022). Answer ALS, a large-scale resource for sporadic and familial ALS combining clinical and multi-omics data from induced pluripotent cell lines. Nat Neurosci *25*, 226–237. https://doi.org/10.1038/s41593-021-01006-0.

Benicky, J., Sánchez-Lemus, E., Honda, M., Pang, T., Orecna, M., Wang, J., Leng, Y., Chuang, D.M., and Saavedra, J.M. (2011). Angiotensin II AT1 receptor blockade ameliorates brain inflammation. Neuropsychopharmacology *36*, 857–870. https://doi.org/10.1038/npp.2010.225.

Boillée, S., Vande Velde, C., and Cleveland, D.W. (2006). ALS: a disease of motor neurons and their nonneuronal neighbors. Neuron *52*, 39–59. https://doi.org/10.1016/j.neuron.2006.09.018.

Boillée, S., Yamanaka, K., Lobsiger, C.S., Copeland, N.G., Jenkins, N.A., Kassiotis, G., Kollias, G., and Cleveland, D.W. (2006). Onset and progression in inherited ALS determined by motor neurons and microglia. Science *312*, 1389–1392. https://doi.org/10.1126/science.1123511.

Chen, T., Xing, J., and Liu, Y. (2018). Effects of telmisartan on vascular endothelial function, inflammation and insulin resistance in patients with coronary heart disease and diabetes mellitus. Exp. Ther. Med. *15*, 909–913. https://doi.org/10.3892/etm.2017.5451.

Christoforidou, E., Joilin, G., and Hafezparast, M. (2020). Potential of activated microglia as a source of dysregulated extracellular microRNAs contributing to neurodegeneration in amyotrophic lateral sclerosis. J. Neuroinflammation *17*, 135. https://doi.org/10.1186/s12974-020-01822-4.

Conlon, E.G., Fagegaltier, D., Agius, P., Davis-Porada, J., Gregory, J., Hubbard, I., Kang, K., Kim, D., Phatnani, H., et al.; New York Genome Center ALS Consortium (2018). Unexpected similarities between C9ORF72 and sporadic forms of ALS/FTD suggest a common disease mechanism. eLife *7*, e37754. https://doi.org/10.7554/eLife.37754.

Dekkers, J.F., Alieva, M., Wellens, L.M., Ariese, H.C.R., Jamieson, P.R., Vonk, A.M., Amatngalim, G.D., Hu, H., Oost, K.C., Snippert, H.J.G., et al. (2019). High-resolution 3D imaging of fixed and cleared organoids. Nat. Protoc. *14*, 1756–1771. https://doi.org/10.1038/s41596-019-0160-8.

Di Giorgio, F.P., Carrasco, M.A., Siao, M.C., Maniatis, T., and Eggan, K. (2007). Non-cell autonomous effect of glia on motor neurons in an embryonic stem cell-based ALS model. Nat. Neurosci. *10*, 608–614. https://doi.org/10.1038/nn1885.

Ehrhart, J., Smith, A.J., Kuzmin-Nichols, N., Zesiewicz, T.A., Jahan, I., Shytle, R.D., Kim, S.H., Sanberg, C.D., Vu, T.H., Gooch, C.L., et al. (2015). Humoral factors in ALS patients during disease progression. J. Neuroinflammation *12*, 127. https://doi.org/10.1186/s12974-015-0350-4.

Femiano, C., Bruno, A., Gilio, L., Buttari, F., Dolcetti, E., Galifi, G., Azzolini, F., Borrelli, A., Furlan, R., Finardi, A., et al. (2024). Inflammatory signature in amyotrophic lateral sclerosis predicting disease progression. Sci. Rep. *14*, 19796. https://doi.org/10.1038/s41598-024-67165-9.

Giacomelli, E., Meraviglia, V., Campostrini, G., Cochrane, A., Cao, X., van Helden, R.W.J., Krotenberg Garcia, A., Mircea, M., Kostidis, S., Davis, R.P., et al. (2020). Human-iPSC-Derived Cardiac Stromal Cells Enhance Maturation in 3D Cardiac Microtissues and Reveal Non-cardiomyocyte Contributions to Heart Disease. Cell Stem Cell *26*, 862–879.e11. https://doi.org/10.1016/j.stem.2020.05.004.

Giacomelli, E., Vahsen, B.F., Calder, E.L., Xu, Y., Scaber, J., Gray, E., Dafinca, R., Talbot, K., and Studer, L. (2022). Human stem cell models of neurodegeneration: From basic science of amyotrophic lateral sclerosis to clinical translation. Cell Stem Cell *29*, 11–35. https://doi.org/10.1016/j.stem.2021.12.008.

Guttikonda, S.R., Sikkema, L., Tchieu, J., Saurat, N., Walsh, R.M., Harschnitz, O., Ciceri, G., Sneeboer, M., Mazutis, L., Setty, M., et al. (2021). Fully defined human pluripotent stem cell-derived microglia and tri-culture system model C3 production in Alzheimer's disease. Nat. Neurosci. *24*, 343–354. https://doi.org/10.1038/s41593-020-00796-z.

Ho, J.K., Moriarty, F., Manly, J.J., Larson, E.B., Evans, D.A., Rajan, K.B., Hudak, E.M., Hassan, L., Liu, E., Sato, N., et al. (2021). Blood-Brain Barrier Crossing Renin-Angiotensin Drugs and Cognition in the Elderly: A Meta-Analysis. Hypertension *78*, 629–643. https://doi.org/10.1161/hypertensionaha.121.17049.

Huang, S., and Zhang, M. (2024). The role of angiotensin II type 1 receptor pathway in cerebral ischemia−reperfusion injury: Implications for the neuroprotective effect of ARBs. Neuroprotection *2*, 100–119. https://doi.org/10.1002/nep3.45.

Joglar, B., Rodriguez-Pallares, J., Rodriguez-Perez, A.I., Rey, P., Guerra, M.J., and Labandeira-Garcia, J.L. (2009). The inflammatory response in the MPTP model of Parkinson's disease is mediated by brain angiotensin: relevance to progression of the disease. J. Neurochem. *109*, 656–669. https://doi.org/10.1111/j.1471-4159.2009.05999.x.

Kim, J.H., Afridi, R., Cho, E., Yoon, J.H., Lim, Y.H., Lee, H.W., Ryu, H., and Suk, K. (2022). Soluble ANPEP Released From Human Astrocytes as a Positive Regulator of Microglial Activation and Neuroinflammation: Brain Renin-Angiotensin System in Astrocyte-Microglia Crosstalk. Mol. Cell. Proteomics *21*, 100424. https://doi.org/10.1016/j.mcpro.2022.100424.

Klinghammer, L., Urschel, K., Cicha, I., Lewczuk, P., Raaz-Schrauder, D., Achenbach, S., and Garlichs, C.D. (2013). Impact of telmisartan on the inflammatory state in patients with coronary atherosclerosis–influence on IP-10, TNF-α and MCP-1. Cytokine *62*, 290–296. https://doi.org/10.1016/j.cyto.2013.02.001.

Labandeira-Garcia, J.L., Rodríguez-Perez, A.I., Garrido-Gil, P., Rodriguez-Pallares, J., Lanciego, J.L., and Guerra, M.J. (2017). Brain Renin-Angiotensin System and Microglial Polarization: Implications for Aging and Neurodegeneration. Front. Aging Neurosci. *9*, 129. https://doi.org/10.3389/fnagi.2017.00129.

Labandeira, C.M., Pedrosa, M.A., Quijano, A., Valenzuela, R., Garrido-Gil, P., Sanchez-Andrade, M., Suarez-Quintanilla, J.A., Rodriguez-Perez, A.I., and Labandeira-Garcia, J.L. (2022). Angiotensin type-1 receptor and ACE2 autoantibodies in Parkinson's disease.

npj Parkinson's Dis. *8*, 76. https://doi.org/10.1038/s41531-022-00340-9.

Lall, D., and Baloh, R.H. (2017). Microglia and C9orf72 in neuroinflammation and ALS and frontotemporal dementia. J. Clin. Invest. *127*, 3250–3258. https://doi.org/10.1172/jci90607.

Lee, H.W., Kim, S., Jo, Y., Kim, Y., Ye, B.S., and Yu, Y.M. (2023). Neuroprotective effect of angiotensin II receptor blockers on the risk of incident Alzheimer's disease: A nationwide population-based cohort study. Front. Aging Neurosci. *15*, 1137197. https://doi.org/10.3389/fnagi.2023.1137197.

Lin, F.C., Tsai, C.P., Kuang-Wu Lee, J., Wu, M.T., and Tzu-Chi Lee, C. (2015). Angiotensin-converting enzyme inhibitors and amyotrophic lateral sclerosis risk: a total population-based case-control study. JAMA Neurol. *72*, 40–48. https://doi.org/10.1001/jamaneurol.2014.3367.

Masrori, P., Beckers, J., Gossye, H., and Van Damme, P. (2022). The role of inflammation in neurodegeneration: novel insights into the role of the immune system in C9orf72 HRE-mediated ALS/FTD. Mol. Neurodegener. *17*, 22. https://doi.org/10.1186/s13024-022-00525-z.

Mennini, T., Giordano, L., Mengozzi, M., Ghezzi, P., Tonelli, R., Mantegazza, R., Silani, V., Corbo, M., Lunetta, C., and Beghi, E. (2009). Increased Il-8 Levels in the Cerebrospinal Fluid of Patients with Amyotrophic Lateral Sclerosis. Eur. J. Inflamm. *7*, 39–44. https://doi.org/10.1177/1721727X0900700105.

Mogi, M., and Horiuchi, M. (2009). Effects of angiotensin II receptor blockers on dementia. Hypertens. Res. *32*, 738–740. https://doi.org/10.1038/hr.2009.110.

Moreau, C., Devos, D., Brunaud-Danel, V., Defebvre, L., Perez, T., Destée, A., Tonnel, A.B., Lassalle, P., and Just, N. (2005). Elevated IL-6 and TNF-alpha levels in patients with ALS: inflammation or hypoxia? Neurology *65*, 1958–1960. https://doi.org/10.1212/01.wnl.0000188907.97339.76.

Nagai, M., Re, D.B., Nagata, T., Chalazonitis, A., Jessell, T.M., Wichterle, H., and Przedborski, S. (2007). Astrocytes expressing ALS-linked mutated SOD1 release factors selectively toxic to motor neurons. Nat. Neurosci. *10*, 615–622. https://doi.org/10.1038/nn1876.

Nakagawa, P., Gomez, J., Grobe, J.L., and Sigmund, C.D. (2020). The Renin-Angiotensin System in the Central Nervous System and Its Role in Blood Pressure Regulation. Curr. Hypertens. Rep. *22*, 7. https://doi.org/10.1007/s11906-019-1011-2.

Noda, A., Fushiki, H., Murakami, Y., Sasaki, H., Miyoshi, S., Kakuta, H., and Nishimura, S. (2012). Brain penetration of telmisartan, a unique centrally acting angiotensin II type 1 receptor blocker, studied by PET in conscious rhesus macaques. Nucl. Med. Biol. *39*, 1232–1235. https://doi.org/10.1016/j.nucmedbio.2012.06.012.

Philips, T., and Rothstein, J.D. (2014). Glial cells in amyotrophic lateral sclerosis. Exp Neurol *262 Pt B*. Exp. Neurol. *262 Pt B*, 111–120. https://doi.org/10.1016/j.expneurol.2014.05.015.

Saxena, S., and Caroni, P. (2011). Selective neuronal vulnerability in neurodegenerative diseases: from stressor thresholds to degeneration. Neuron *71*, 35–48. https://doi.org/10.1016/j.neuron.2011.06.031.

Sigmund, C.D., and Grobe, J.L. (2020). A colorful view of the brain renin-angiotensin system. Hypertens. Res. *43*, 357–359. https://doi.org/10.1038/s41440-020-0396-2.

Takagi, H., Mizuno, Y., Yamamoto, H., Goto, S.N., and Umemoto, T.; All-Literature Investigation of Cardiovascular Evidence Group (2013). Effects of telmisartan therapy on interleukin-6 and tumor necrosis factor-alpha levels: a meta-analysis of randomized controlled trials. Hypertens. Res. *36*, 368–373. https://doi.org/10.1038/hr.2012.196.

Tchieu, J., Calder, E.L., Guttikonda, S.R., Gutzwiller, E.M., Aromolaran, K.A., Steinbeck, J.A., Goldstein, P.A., and Studer, L. (2019). NFIA is a gliogenic switch enabling rapid derivation of functional human astrocytes from pluripotent stem cells. Nat. Biotechnol. *37*, 267–275. https://doi.org/10.1038/s41587-019-0035-0.

Tchieu, J., Zimmer, B., Fattahi, F., Amin, S., Zeltner, N., Chen, S., and Studer, L. (2017). A Modular Platform for Differentiation of Human PSCs into All Major Ectodermal Lineages. Cell Stem Cell *21*, 399–410.e7. https://doi.org/10.1016/j.stem.2017.08.015.

Tortelli, R., Zecca, C., Piccininni, M., Benmahamed, S., Dell'Abate, M.T., Barulli, M.R., Capozzo, R., Battista, P., and Logroscino, G. (2020). Plasma Inflammatory Cytokines Are Elevated in ALS. Front. Neurol. *11*, 552295. https://doi.org/10.3389/fneur.2020.552295.

Vahsen, B.F., Gray, E., Candalija, A., Cramb, K.M.L., Scaber, J., Dafinca, R., Katsikoudi, A., Xu, Y., Farrimond, L., Wade-Martins, R., et al. (2022). Human iPSC co-culture model to investigate the interaction between microglia and motor neurons. Sci. Rep. *12*, 12606. https://doi.org/10.1038/s41598-022-16896-8.

Vahsen, B.F., Nalluru, S., Morgan, G.R., Farrimond, L., Carroll, E., Xu, Y., Cramb, K.M.L., Amein, B., Scaber, J., Katsikoudi, A., et al. (2023). C9orf72-ALS human iPSC microglia are pro-inflammatory and toxic to co-cultured motor neurons via MMP9. Nat. Commun. *14*, 5898. https://doi.org/10.1038/s41467-023-41603-0.

Wanderer, S., Grüter, B.E., Strange, F., Sivanrupan, S., Di Santo, S., Widmer, H.R., Fandino, J., Marbacher, S., and Andereggen, L. (2020). The Role of Sartans in the Treatment of Stroke and Subarachnoid Hemorrhage: A Narrative Review of Preclinical and Clinical Studies. Brain Sci. *10*, 153. https://doi.org/10.3390/brainsci10030153.

Wosiski-Kuhn, M., Caress, J.B., Cartwright, M.S., Hawkins, G.A., and Milligan, C. (2021). Interleukin 6 (IL6) level is a biomarker for functional disease progression within IL6R(358)Ala variant groups in amyotrophic lateral sclerosis patients. Amyotroph. Lateral Scler. Frontotemporal Degener. *22*, 248–259. https://doi.org/10.1080/21678421.2020.1813310.

Zhang, W., Xiao, D., Mao, Q., and Xia, H. (2023). Role of neuroinflammation in neurodegeneration development. Signal Transduct. Target. Ther. *8*, 267. https://doi.org/10.1038/s41392-023-01486-5.

Stem Cell Reports, Volume *20*

Supplemental Information

Telmisartan is neuroprotective in a hiPSC-derived spinal microtissue model for C9orf72 ALS via inhibition of neuroinflammation

Berkiye Sonustun, Björn F. Vahsen, Mario Ledesma-Terrón, Zhuoning Li, Laura Tuffery, Nan Xu, Elizabeth L. Calder, Johannes Jungverdorben, Leslie Weber, Aaron Zhong, David G. Miguez, Mara Monetti, Ting Zhou, Elisa Giacomelli, and Lorenz Studer

Supplemental Information:

Telmisartan is neuroprotective in a hiPSC-derived spinal microtissue model for C9orf72-ALS via inhibition of neuroinflammation.

Berkiye Sonustun, Björn F. Vahsen, Mario Ledesma-Terrón, Zhuoning Li, Laura Tuffery , Nan Xu, Elizabeth L. Calder, Johannes Jungverdorben, Leslie Weber, Aaron Zhong, David Miguez Gómez, Mara Monetti, Ting Zhou, Elisa Giacomelli and Lorenz Studer.

- **Supplementary Methods:** Pages **1-6**

- **Supplementary Figures and Figure legends**
  - Figure S1 Pages **7-8**
  - Figure S2 Pages **9-10**
  - Figure S3 Pages **11-12**
  - Figure S4 Pages **13-14**

Supplementary Methods

hiPSC Cell Culture

Mycoplasma testing was performed upon thawing the MCB and upon receiving isogenic pairs from the MSKCC Stem Cell Core. Sterility of cultures was monitored through daily observations. Genomic characterization of MCBs was conducted by the Answer ALS repository, and karyotyping analysis was performed both when MCBs were thawed to generate WCBs (for Answer ALS and in-house isogenic pairs) and before the high-throughput screen. Confirmation of the disease mutation was performed by Answer ALS. Removal of the HRE in isogenic lines was confirmed by the MSKCC Stem Cell Core during their generation. All stem cell work was conducted in accordance with protocols approved by the Tri-Institutional Stem Cell Initiative Embryonic Stem Cell Research Oversight Committee (Tri-SCI ESCRO).

Generation of 3D, spinal motor neurons (MNs) organoids from hiPSCs in 3D

hiPSCs were dissociated using 0.04% EDTA-PBS solution and seeded at 10,000 cells/well of a V-bottom 96 well plate (S-Bio PrimeSurface® 3D culture: Ultra-low Attachment Plates, Cat # #MS-9096VZ) in E8 media containing ROCK inhibitor (Y-27632; 10 µM) on Day -1. On Days 0, 2 and 4, media was changed to Neurobasal media containing N2 and B27 supplements (NB N2/B27),

and neural induction was performed via dual SMAD inhibition with LDN193189 (250 nM; Stemgent) and SB431542 (10 µM; Tocris Bioscience) and Anterior-Posterior patterning via the Wnt-activator CHIR 99021 (3uM; Tocris). Ascorbic Acid (AA) (100 nM; Sigma) was supplemented to the media as an antioxidant. On Day 6, organoids were transferred to 10 cm dishes on an orbital shaker and maintained in NB N2/B27 media containing SB431542 (2 µM, R&D), LDN193189 (500 nM, Stemgent), AA (100 nM, Sigma), CHIR 99201 (1 µM, Tocris Bioscience), SAG (1 µM, Selleck) and Retinoic Acid (RA) (100 nM, Sigma) with media changes on days 8 and 10. On Day 11, neural progenitors were assessed for the expression of NKX 6.1 and Olig2. Within its broader expression domain, Nkx6.1 initiates transcription of Olig2 specifically in the pMN domain 64-67. Olig2 distinguishes MN progenitors from V2 interneuron progenitors that also express Nkx6.1 (Novitch et al., 2001). On Day 13, dual SMAD inhibition was completed, and cells were cultured in NB N2/B27 media containing SAG (0.5 µM, Selleck) RA (500 nM, Sigma), AA (100 nM, Sigma), BDNF (0.02 µg/ml, R&D), GDNF (0.02 µg/ml, Peprotech), and CNTF (0.01 µg/ml, R&D). On Days 15 and 17, organoids were cultured in Day 13 media supplemented with DAPT (10 µM; Tocris Bioscience). From Day 20, organoids were maintained in NB media containing B27 (NB/B27) supplemented with SAG (0.5 µM, Selleck), Retinoic Acid (RA) (500 µM, Sigma), DAPT (10 µM; Tocris Bioscience) and CNTF (0.01 µg/ml, R&D). Day 20-30 organoids were dissociated as described below and cells were subjected to quality control (QC), immunofluorescence (IF) experiments to check for Islet-1 (ISL1), HUC/D, MAP2 and TUJ1.

Generation of spinal astrocytes from hiPSCs

On Day 0, hiPSCs were dissociated into a single-cell suspension using Accutase and plated on Geltrex coated plates at 600,000 cells per cm2 in NB N2/B27 media containing SB431542 (10 µM, Tocris Bioscience), LDN193189 (250 µM, Stemgent) Ascorbic Acid (AA) (100 µM; Sigma), CHIR99201 (3 µM; Tocris Bioscience) and ROCK inhibitor (10 µM; Y-27632). Between days 1 and 5, day 0 media without ROCK inhibitor was used to culture cells with media changes every day. Between days 6 and 10, cells were cultured in NB N2/B27 media supplemented with SB431542 (2µM; Tocris Bioscience), LDN193189 (500 nM; Stemgent), AA (100 nM; Sigma), CHIR99201 (1 µM; Tocris Bioscience), SAG (1 µM; Selleck) and Retinoic Acid (RA) (100 nM; Sigma). On Day 11, neural progenitors were dissociated with Accutase solution containing DNAse I (50 µg/ml Sigma/Roche) and plated on Poly-D-lysine (10 µg/ml; Sigma) Fibronectin (2 µg/ml; Corning), Laminin (2 µg/ml; R&D) coated plates at 175,000 cells per cm2 in NB N2/B27 media containing SAG (0.5 µM; Selleck), Retinoic Acid (RA) (500 nM; Sigma), AA (100 nM; Sigma), BDNF (0.02 µg/ml, R&D) GDNF (0.02 µg/ml, Peprotech), CNTF (0.01 µg/ml, R&D) and ROCK

inhibitor (Y-27632; 10 μM). Progenitors were subjected to QC to check for NKX 6.1 and Olig2 expression via IF. On day 12, progenitors were infected with lentiviral vectors containing NFIA (https://www.addgene.org/141403/) and M2rtTA (https://www.addgene.org/20342/) overnight to induce a gliogenic switch of neural progenitors into astrocytes as described in 30 . On days 14 to 20, cells were cultured in Day 13 media supplemented with1 μg/ml Doxycycline to induce NFIA expression and on day 20, cells were subjected to QC to check for NFIA and Sox9 expression via IF. On day 22, cells were dissociated using trypsin and re-plated at 50,000 cells per cm2 in plates coated with Poly-L-Ornithine Hydrobromide (15 μg/ml; Sigma), Fibronectin (2 μg/ml; Corning) and Laminin (2 μg/ml; R&D) in Astrocyte Medium (AM, ScienCell Research Laboratories, Cat. #1801) with 2% fetal bovine serum from the AM kit, for two weeks before removing FBS from the media completely. Cryobanks were generated on Day 22, Day 35, Day 45 and Day 50 cells and cells were subjected to QC, performed by IF to check for astrocyte markers on Day 50.

Generation of microglia from hiPSCs

hiPSCs were dissociated into a single-cell suspension using Accutase on Day 0, and 60,000 cells per cm2 were seeded onto Matrigel-coated plates in Essential 8 medium with activin A (7.5 ng/ml; R&D), BMP4 (30 ng/ml; R&D), CHIR 99021 (3 μM; Tocris Bioscience) and ROCK inhibitor (Y-27632; 10 μM). Following a 16 to18 hour period, Day 0 media was changed to Essential 6 medium based Day 1 media containing activin A (10 ng/ml; R&D), BMP4 (40 ng/ml; R&D) and IWP2 (2 μM; Selleck;). On day 2, cells were exposed to FGF2 (20 ng/ml; Selleck;) that was added to the Day 1 media. The next day, cells were dissociated with Accutase and re-seeded at 60,000 cells per cm2 in Matrigel coated plates in Essential 6 medium based Day 3 media containing ROCK inhibitor (Y-27632; 10 μM). VEGF (15 ng/ml; R&D,) and FGF2 (5 ng/ml; R&D,). On Day 4, and cell medium was changed to Day 3 media without the ROCK inhibitor. Cells were exposed to Essential 6 based media containing VEGF (15 ng/ml), FGF2 (5 ng/ml), SCF (200 ng/ml; R&D) and IL-6 (20 ng/ml; R&D) on Days 5 and 6, followed by media change to Essential 6 containing SCF (100 ng/ml), IL-6 (10 ng/ml), TPO (30 ng/ml; R&D) and IL-3 (30 ng/ml; R&D) on Day 7 and Day 9. Distinctively, we did not co-culture EMP, PMACs with neurons, instead harvesting the round cells into RPMI media containing 10% FBS, and IL-34 (100 ng/ml; R&D) and M-CSF (10 ng/ml; R&D) directly for 18-20 days until round cells were adherent and exhibited classical microglia morphology and expressed microglia specific markers. Cells were subjected to QC, performed by IF to check for the expression of microglial markers and cryobanks were generated using 90% FBS and 10% DMSO as freezing media.

Dissociation of spinal MN organoids

Samples were sliced using a sterile scalpel into smaller pieces and resuspended into papain solution. The samples were incubated at 37C with constant agitation on an orbital shaker for 1 h. Following incubation, the papain-cell solutions were pipetted up and down with a micropipette (P1000) to completely dissolve any tissue pieces. The samples were then transferred to 15 mL falcon tubes and centrifuged at 300g for 5 min at room temperature. Supernatants were discarded and cell pellets were immediately resuspended in DNase dilute albumin-inhibitor solution. Subsequently, albumin-inhibitor solution was added drop-by-drop to each sample to create phase separation and tubes were centrifuged at 70g for 6 min at room temperature. Cells were resuspended in Neurobasal medium and counted to be incorporated into SMs.

Whole Mount Staining of Microtissues

Microtissues were fixed in 4% paraformaldehyde for 1 hour at 4oC, and washed 3x in PBS. After the third wash, PBS was removed and microtissues were incubated in 0.5mL of 0,5% Triton-X in PBS at RT for 6 hours. Subsequently, microtissues were re-suspended in Organoid Washing Buffer, OWB (0.2% Triton-X, 0.02% SDS and 0.2% BSA in PBS) and transferred to a low adhesion 24 well plate and incubated for 15 minutes at RT. Primary antibodies were prepared in OWB and incubated overnight on an orbital shaker at 37oC. The next day, microtissues were washed for a total of 6 hours with OWB inside the wells (3 washes, 2 hours per wash) and the secondary antibodies were prepared in OWB and added to the microtissues inside the wells and incubated overnight at 37oC on the orbital shaker. On the final day, microtissues were subjected to 3X OWB washes for a total of 6 hours, recovered with a P200 micropipette, and mounted on slides for confocal microscopy.

Cytokine Arrays

Fourteen markers were simultaneously measured in the samples using Eve Technologies' Human High Sensitivity 14-Plex Discovery Assay® (MilliporeSigma, Burlington, Massachusetts, USA, HDHSTC14) according to the manufacturer's protocol. The 14-plex consisted of GM-CSF, IFNγ, IL-1β, IL-2, IL-4, IL-5, IL-6, IL-8, IL-10, IL-12p70, IL-13, IL-17A, IL-23, TNF-α. Assay sensitivities of these markers range from 0.11 – 3.25 pg/mL for the 14-plex. Individual analyte sensitivity values are available in the MilliporeSigma MILLIPLEX® MAP protocol. The measurements were performed on a bead analyzer (Bio-Plex 200), which detects the amount of the target analyte. The results are quantified according to a standard curve.

Live imaging with BioTek Cytation 5 Cell Imaging Multimode Reader

Cells were initially seeded into 96-well plates on D0. These plates were incubated under standard cell culture conditions (37°C, 5% CO_2) until they adhered. The next day (D1), wells were treated with 0.01% DMSO or 5 µM telmisartan (final concentration in 100ul media) and incubated overnight under standard cell culture conditions. Prior to imaging, the BioTek Cytation 5 was configured to maintain the cells at 37°C and 5% CO_2 during the entire imaging process. The plates were loaded onto the Cytation 5. Imaging commenced on Day 2 after cell seeding, and the Cytation 5 was programmed to capture both brightfield and fluorescent images (targeting the Td-tomato channel) every 4 hours over a 14-day period. Exposure settings, focus parameters, and fluorescence intensity thresholds were optimized and standardized across all wells to ensure consistent image acquisition. Every 72 hours, a complete media change was performed to refresh the media containing either telmisartan or DMSO.

Gene correction and validation for C9orf72 ALS iPSC line

Two nicking sgRNAs were designed targeting to the upstream and downstream close to the repeat sequence. Each target sequence was cloned into the pX335 vector (Addgene, Plasmid #42335) to make the gene targeting constructs (Scheme: slide 3). A donor plasmid containing a 586 bp left homology arm, followed by a "loxp-PGK-puro-loxp" cassette, a normal 2 G4C2 repeat, and a 652 bp right homology arm was used as the template for HDR (Scheme: slide 3). The nicking sgRNAs and the donor plasmid were electroporated into patients' iPSCs using a Lonza 4D-Nucleofector instrument with Solution "Primary Cell P3", and Pulse Code "CB-150". 0.5 ug/ml Puromycin was added to the 3 days post-electroporation cells for 4 days. Single-cell clones were then generated, PCR and Sanger-sequencing33 were used to identify the correctly knock-in clones that Knock-in to the mutant allele, and with the WT allele untouched. For the identified corrected Knock-in clones, Cre treatment to remove the "loxp-PGK-puro-loxp" cassette and left a "loxP" and a normal 2 G4C2 repeat at the genome locus.

sgRNA sequences and PCR primers:

C9orf72- up sgRNA target	GCTCTCACAGTACTCGCTGA
C9orf72- down sgRNA target	GAAAGCCCGACACCCAGCTT
C9orf72-PCR-F1	AGGTGTAGACGTTGAGAGCC
C9orf72-PCR-R1	CTGAAATTGTGCAGGCGTCT

3D Image Processing and Analysis

Kernel Size Determination: Kernel size, a critical parameter for image filtering and analysis, was estimated using a two-step approach. Initially, a difference image was generated by subtracting the average z-projection from the maximum z-projection. Median filtering and Euclidean Distance Transform (EDT) were applied to this image, with local maxima in the EDT corresponding to initial kernel size estimates. To refine this estimate, the top 5% densest image slices were subjected to background subtraction and median filtering using the initial kernel size. Local maxima in the EDT of these refined slices provided the final kernel size for subsequent image processing.

Nuclear Segmentation and Characterization: DAPI staining was used to identify and localize nuclei within the image volume. To enhance image quality, DAPI images underwent Gaussian blurring and Unsharp Masking. A combination of thresholding, background subtraction, and watershed segmentation was employed to delineate individual nuclei. The resulting 3D nuclear objects were characterized in terms of their position, orientation, and dimensions using an algorithm of clustering in 3D described in Ledesma-Terrón et al.34.

Marker Analysis and Quantification: Isl1, Iba1, and GFAP channels were subjected to median filtering, with a global intensity threshold differentiating signal from background. To reduce noise in the GFAP channel, a grayscale opening operation was applied. To extract quantitative information, the mean marker intensity inside each nuclear volume is looked for all markers, and phenotypes were assigned based on comparative analysis of standardized intensities for each marker.

Data Analysis: To assess temporal changes in marker expression, data were categorized based on experimental conditions (C9-ALS+DMSO, C9-ALS + telmisartan, CTRL+DMSO, CTRL + telmisartan). Statistical comparisons of mean marker intensities across time points were performed to identify significant differences.

Supplemental Figure S1 (Sonustun et al.,)

A

iPSC Line Name	Disease	Parent Cell Type	Sex	Revised El Escorial Criteria	Age at Sample Collection	Age at Death	Cause of Death	Baseline ALSFRS-R
CS7VCZi ALS-n3	C9ORF72 ALS	PBMC/ T-Cell	Male	Definite	64	65	Disease Progression	-2.9324
CS5DZLi CTR-n5	Healthy CTRL	PBMC	Male	N/A	64	N/A	N/A	N/A

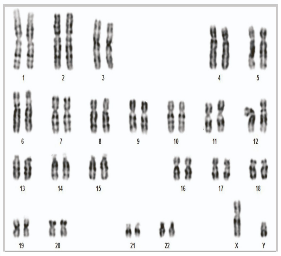

7VCZ (C9-ALS) - 46, XY

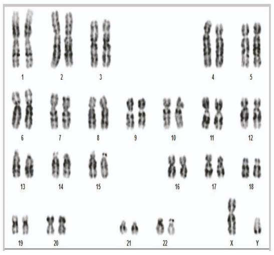

5DZL (CTRL) - 46, XY

B

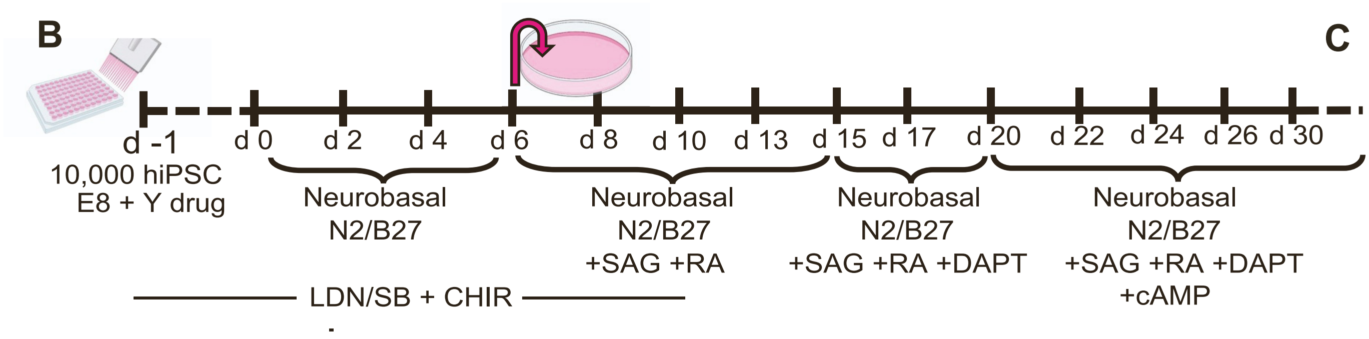

C

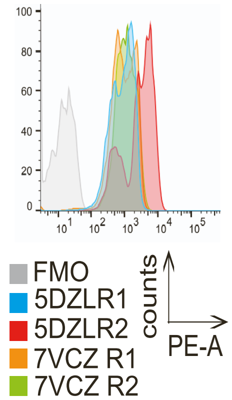

D

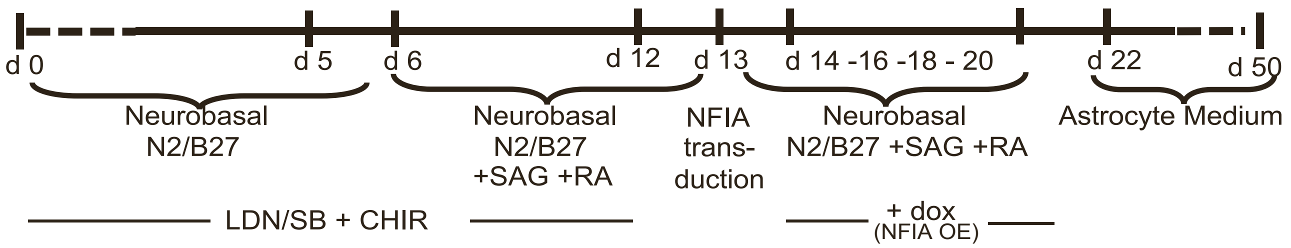

E

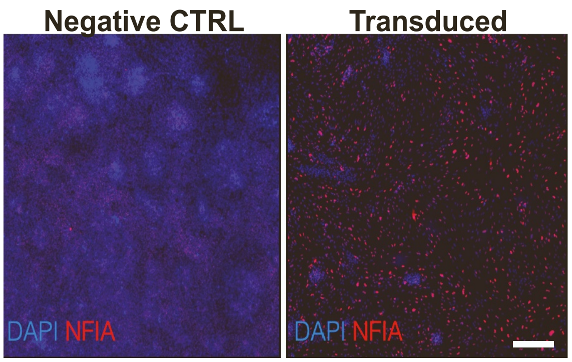

F

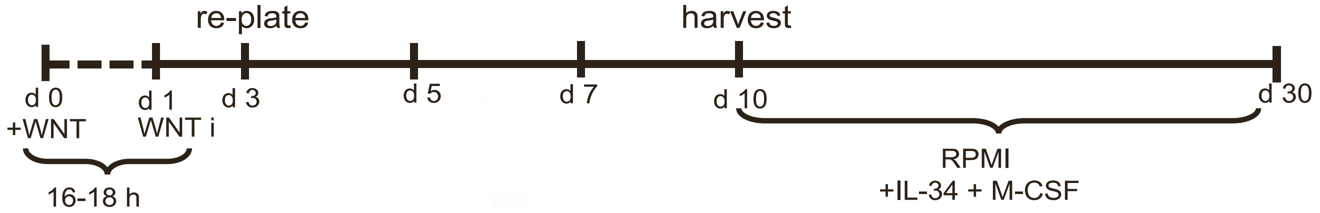

G

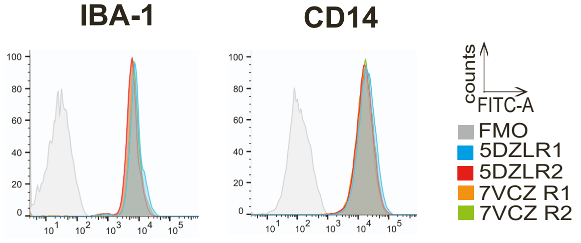

Figure S1. Characterization of hiPSC-Derived Spinal Microtissues – related to Fig. 1

(A) Details and karyotype analysis of the C9-ALS and age- and gender-matched healthy control (CTRL) iPSC lines used in this study. The C9-ALS iPSC lines were derived from patients diagnosed with ALS harboring the C9orf72 hexanucleotide repeat expansion, while the healthy CTRL iPSC lines were obtained from individuals without neurodegenerative diseases.

(B) Schematic of spinal motor neuron (MN) differentiation from hiPSCs in 3D culture. Starting with 10,000 cells per well in a low-attachment 96-well V-bottom plate, the protocol involves dual-SMAD inhibition and anterior-posterior and ventral patterning, resulting in high-purity ISL-1 positive 3D spinal MN organoids within 20-30 days.

(C) Representative flow cytometry analysis showing ISL-1 positivity in Day 30 (d30) hiPSC-derived spinal MN organoids from both healthy control and C9-ALS patients following papain dissociation. (FMO: fluorescence minus one).

(D) Schematic representing the hiPSC-derived spinal astrocyte differentiation protocol adapted from Tchieu et al. Spinal astrocytes are obtained by overexpressing NFIA in NSCs, promoting a glial fate switch in progenitors.

(E) Immunofluorescence characterization of NFIA transduction efficiency in neural progenitor cells (NPCs) on Day 20 (D20). The left panel shows NPCs transduced with a non-targeting virus, stained with DAPI (blue) for nuclear visualization and NFIA (red) as a control, indicating minimal or no expression of NFIA. The right panel displays NPCs transduced with the NFIA-targeting virus, demonstrating successful transduction, as evidenced by the widespread presence of NFIA-positive (red) nuclei throughout the culture. Scale bar corresponds to 100μm.

(F) Schematic illustrating the hiPSC-derived microglia differentiation protocol adapted from Guttikonda et al. The protocol directs hiPSCs to differentiate into microglial precursors by mimicking primitive hematopoiesis. Hemangioblast precursors are exposed to hematopoietic cytokines, generating macrophage precursors that transition into microglia expressing markers such as Pu.1 and IBA-1.

(G) Flow cytometry analysis showing IBA-1 and CD14 positivity in Day 30 (d30) hiPSC-derived microglia from healthy control and C9-ALS following Accutase dissociation. (FMO: fluorescence minus one).

Supplemental Figure S2 (Sonustun et al.,)

A

Abbreviation	Name
UK	UK 14,304
BRC	Bromocriptine mesylate
DHE	Dihydroergotamine mesylate
DBT	Dobutamine hydrochloride
MXD	Minoxidil
PRZ	Prazosin hydrochloride
DIL	Diltiazem hydrochloride
DIP	Dipyridamole
CYT	Cyclothiazide
FMB	Felbamate
CIM	Cimetidine
TPM	Tropicamide
GBC	Glibenclamide
PMZ	Pimozide
DXZ	Diazoxide
TMX	Tamoxifen citrate
ICI	ICI 182,780
TXL	Taxol
DEX	Dexamethasone
ETP	Etoposide

Abbreviation	Name
FMZ	Flumazenil
CLF	Clemastine fumarate
FLC	Flecainide acetate
MFP	Mifepristone
MPA	Mycophenolic acid
LOV	Lovastatin
ARG	Argatroban
CLZ	Cilostazol
CSP	Cisapride
ACT	Acetaminophen
LRT	Loratidine
SIM	Simvastatin
ISR	Isradipine
FLP	Fluticasone propionate
MRP	Mirtazapine
TBZ	Tetrabenazine
DOX	Doxorubicin hydrochloride
RAL	Raloxifene hydrochloride
FLX	Fluoxetine hydrochloride
FEX	Fexofenadine hydrochloride

Abbreviation	Name
ACV	Acyclovir
AML	Amlodipine besylate
BEN	Benazepril hydrochloride
MEX	Mexiletine hydrochloride
CLF	Clofarabine
DCT	Decitabine
ZON	Zonisamide
CAB	Cabergoline
BDS	Budesonide
ACR	Acarbose
PBA	Sodium 4-Phenylbutyrate
CAR	Carvedilol
CPT	CPT 11
TMZ	Temozolomide
DMD	Dexmedetomidine hydrochloride
IBU	(S)-(+)-Ibuprofen
LVT	Levetiracetam
ADP	Adapalene
MET	Metformin hydrochloride
VEN	Venlafaxine hydrochloride

Abbreviation	Name
FLD	Felodipine
DOX	Doxazosin mesylate
SPR	Spironolactone
IRE	Iressa
MCZ	Miconazole nitrate
TAM	Tamsulosin hydrochloride
BMT	Bumetanide
FRS	Furosemide
RAN	Ranolazine dihydrochloride
MTP	Metoprolol tartrate
GEM	Gemcitabine hydrochloride
VPA	Valproic acid, sodium salt
MTP	Metyrapone
ZLT	Zileuton
FLV	Fluvastatin sodium
SLX	Selexipag
FLD	Fludarabine
NEB	Nebivolol hydrochloride
SUM	Sumatriptan succinate
TIZ	Tizanidine hydrochloride

Abbreviation	Name
TOP	Topiramate
ASN	Asenapine maleate
MRV	Maraviroc
DFT	Dofetilide
EXE	Exemestane
VAR	Varenicline tartrate
LNZ	Linezolid
SUN	Sunitinib malate
AZM	Azithromycin
SLF	Sildenafil citrate
DOC	Docetaxel
LST	Losartan potassium
RPG	Repaglinide
CP	CP 690550 citrate
TRN	Tranylcypromine hydrochloride
ABT	ABT 199
TRV	Trovafloxacin mesylate
RTG	Rotigotine hydrochloride
IBL	Ibutilide hemifumarate
PYM	Pyrimethamine

Abbreviation	Name
ASP	Aspirin
HCT	Hydrocortisone
FLU	Flutamide
CLT	Clotrimazole
CRB	Carbamazepine
AZA	Azathioprine
MMF	Mycophenolate mofetil
PRB	Probenecid
FFB	Fenofibrate
BPR	Bepridil hydrochloride
PIO	Pioglitazone hydrochloride
ABA	Abacavir hemisulfate
DEL	Delavirdine mesylate
VAL	Valsartan
NTL	Nateglinide
OLO	Olopatadine hydrochloride
MCL	Meclizine dihydrochloride
TGB	Tiagabine hydrochloride
RSG	Rasagiline mesylate
OLZ	Olanzapine

Abbreviation	Name
AXI	Axitinib
SQV	Saquinavir mesylate
RVG	Rivastigmine tartrate
TFT	Trifluorothymidine
RBV	Ribavirin
AZL	Azilsartan
OLM	Olmesartan
SAHA	SAHA
ZLM	Zolmitriptan
SLT	Salmeterol xinafoate
QTP	Quetiapine hemifumarate
CND	Candesartan
DLX	(S)-Duloxetine hydrochloride
CAP	Capecitabine
MPS	Methyl-prednisolone
LNG	Levonorgestrel
AMX	Amlexanox
RUX	Ruxolitinib
PTV	Pitavastatin calcium
MGT	Miglitol

Abbreviation	Name
STV	Stavudine
RZT	Rizatriptan benzoate
TLM	Telmisartan
TLV	Tolvaptan
RMP	Ramipril
BZA	Bazedoxifene acetate
RSG	Rosiglitazone
RFX	Rifaximin
XL	XL 184
ARP	Aripiprazole
REM	Remdesivir
IRB	Irbesartan
AMB	Ambrisentan
RTV	Ritonavir
IMT	Imatinib mesylate
FTY	FTY 720
PMT	Pemetrexed
ARM	Arformoterol tartrate
RUC	Rucaparib camsylate
BOS	Bosentan

Abbreviation	Name
ANZ	Anastrozole
BCL	Bicalutamide
BOS	Bosutinib
LTZ	Letrozole
FBS	Febuxostat
ESC	Escitalopram oxalate
TRF	Teriflunomide
ALK	Aliskiren hemifumarate
VLZ	Vilazodone hydrochloride
SAX	Saxagliptin hydrochloride
PF	PF 04449913 maleate
DRV	Darunavir
DST	Dasatinib
IBR	Ibrutinib
ETV	Entecavir
MGA	Megestrol Acetate
LND	Lenalidomide
TDL	Tadalafil
PRG	Prasugrel
TRZ	Trazodone hydrochloride

Abbreviation	Name
RSV	Rosuvastatin calcium
CCS	Ciclesonide
CCP	Ciclopirox
PNT	Phentolamine Mesylate
APR	Aprepitant
RFL	Roflumilast
SLN	Silodosin
LPT	Lapatinib
SRF	Sorafenib
MFQ	Mefloquine hydrochloride

B

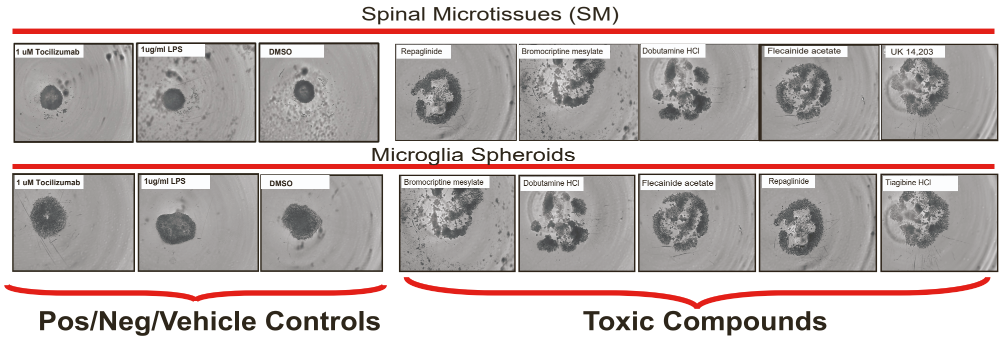

C

	Condition	Z' Score
IL-6	Microglia Spheroids	0.853
	Spinal Microtissues	0.6889
IL-8	Microglia Spheroids	0.7625
	Spinal Microtissues	0.9127

D

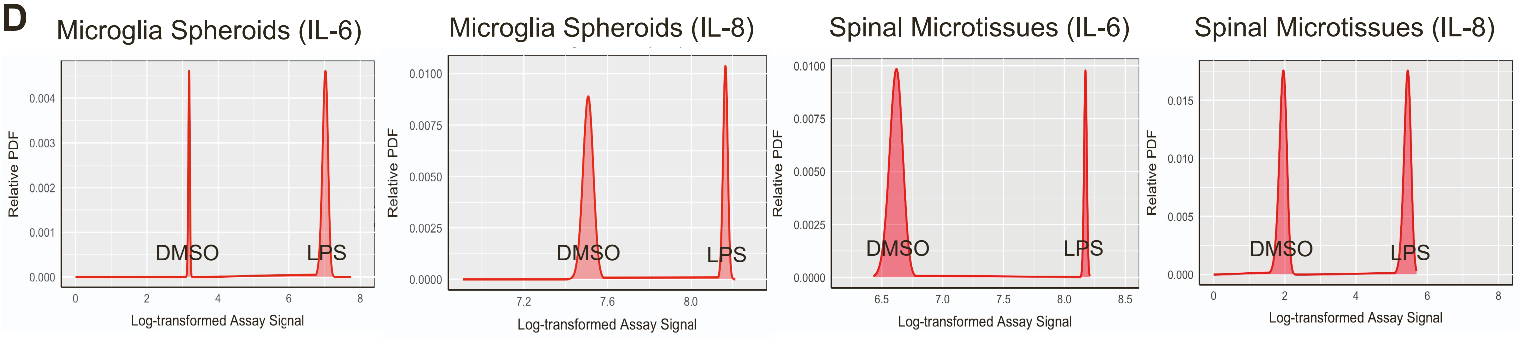

E

Drug	Condition	EC50
Telmisartan	IL-6	1.2 μM
	IL-8	3.9 μM
Valsartan	IL-6	9.7 μM
	IL-8	15 μM
Azilsartan	IL-6	0.16 μM
	IL-8	3.2 μM

Figure S2. Assay Validity and details about compounds – related to Fig. 2

(A) Table showing compound abbreviations in 3-letter codes used in the screen. This table provides a reference for interpreting the hit identification in Figure 2(d).

(B) Cell viability was assessed by measuring the microtissue/spheroid diameters before and 72 hours after library application, and toxic compounds were excluded from analysis. Representative images of toxicity, as well as cells treated with the positive, negative and vehicle controls are shown.

(C) Z' scores for IL-6 and IL-8 cytokine assays conducted on microglia spheroids and spinal microtissues.

(D) Z-prime factor graphs were generated based on relative probability distribution factors on the y-axis, and the extrapolated assay signals of negative and vehicle control data on the x-axis. The peaks represent the separation between the positive control (LPS, right peak) and the vehicle control (DMSO, left peak). These graphs utilize the standard deviations, means, and medians of the control data to depict the assay performance. Z-prime scores and assay validity for the high-throughput screen of 190 FDA-approved compounds targeting neuroinflammation in C9-ALS. All conditions yielded Z-prime factors of above 0.5 indicating high assay quality and reliability.

Supplemental Figure S3 (Sonustun et al.,)

A

Control Microglia Spheroids

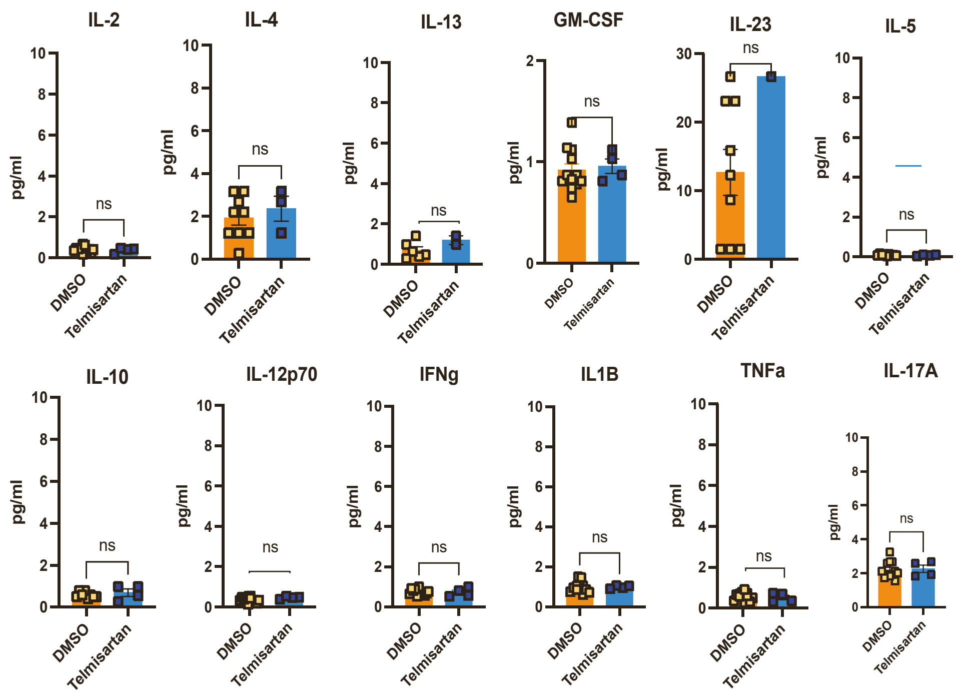

B

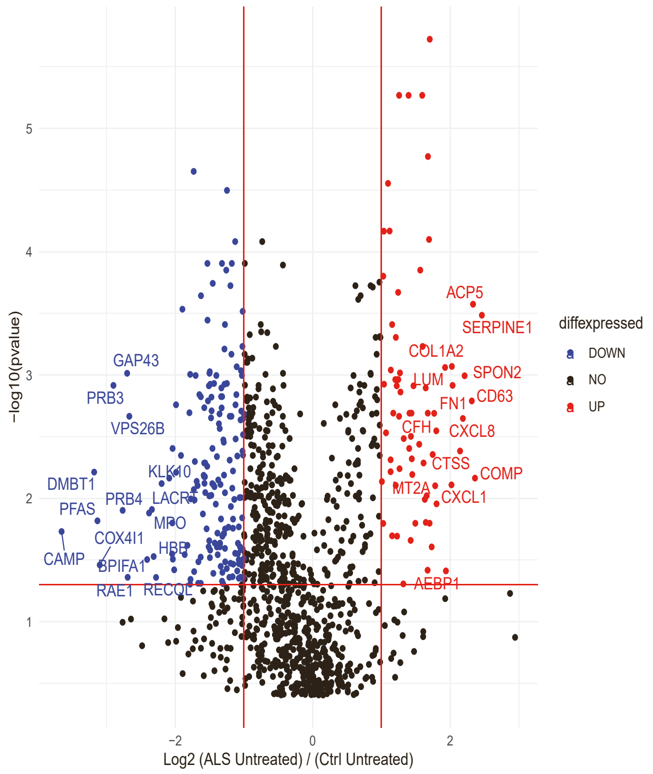

C

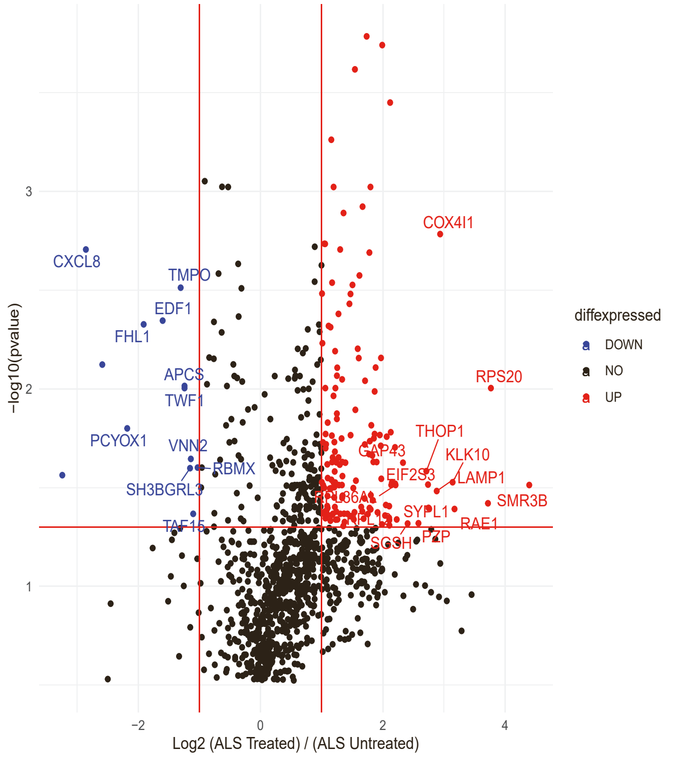

Figure S3. Telmisartan does not affect cytokine levels in supernatant of CTRL SMs - related to Fig. 3

(A) Bar graphs depicting cytokine concentrations from untreated and 5 μM telmisartan treated CTRL SMs. Results show that Telmisartan treatment shows no impact on cytokine levels in CTRL SMs. Student's T-Test N = 3 replicates, 3 independent differentiations. Data are represented as Mean ± SEM.

(B) Unbiased secretome proteomic analysis of untreated C9-ALS and control SM supernatants. Volcano plot depicting significantly different proteins between C9-ALS and control SMs (blue = significantly downregulated proteins in C9-ALS, red = significantly upregulated proteins in C9-ALS compared to controls). IL-8 (CXCL8) is among the top upregulated proteins in the C9-ALS secretome compared to controls, validating our findings. NB: 'Untreated' refers to DMSO-treated.

(C) Unbiased secretome proteomic analysis of untreated C9-ALS and C9-ALS SMs treated with 5 μM Telmisartan. Volcano plot depicting significantly different proteins between untreated C9-ALS and Telmisartan-treated C9-ALS SMs (blue = significantly downregulated proteins in C9-ALS + telmisartan, red = significantly upregulated proteins in C9-ALS + telmisartan compared to untreated C9-ALS). IL-8 (CXCL8) is the top downregulated protein in the telmisartan-treated C9-ALS secretome compared to untreated C9-ALS, validating our HTS findings. NB: 'Untreated' refers to DMSO-treated.

Supplemental Figure S4 (Sonustun et al.,)

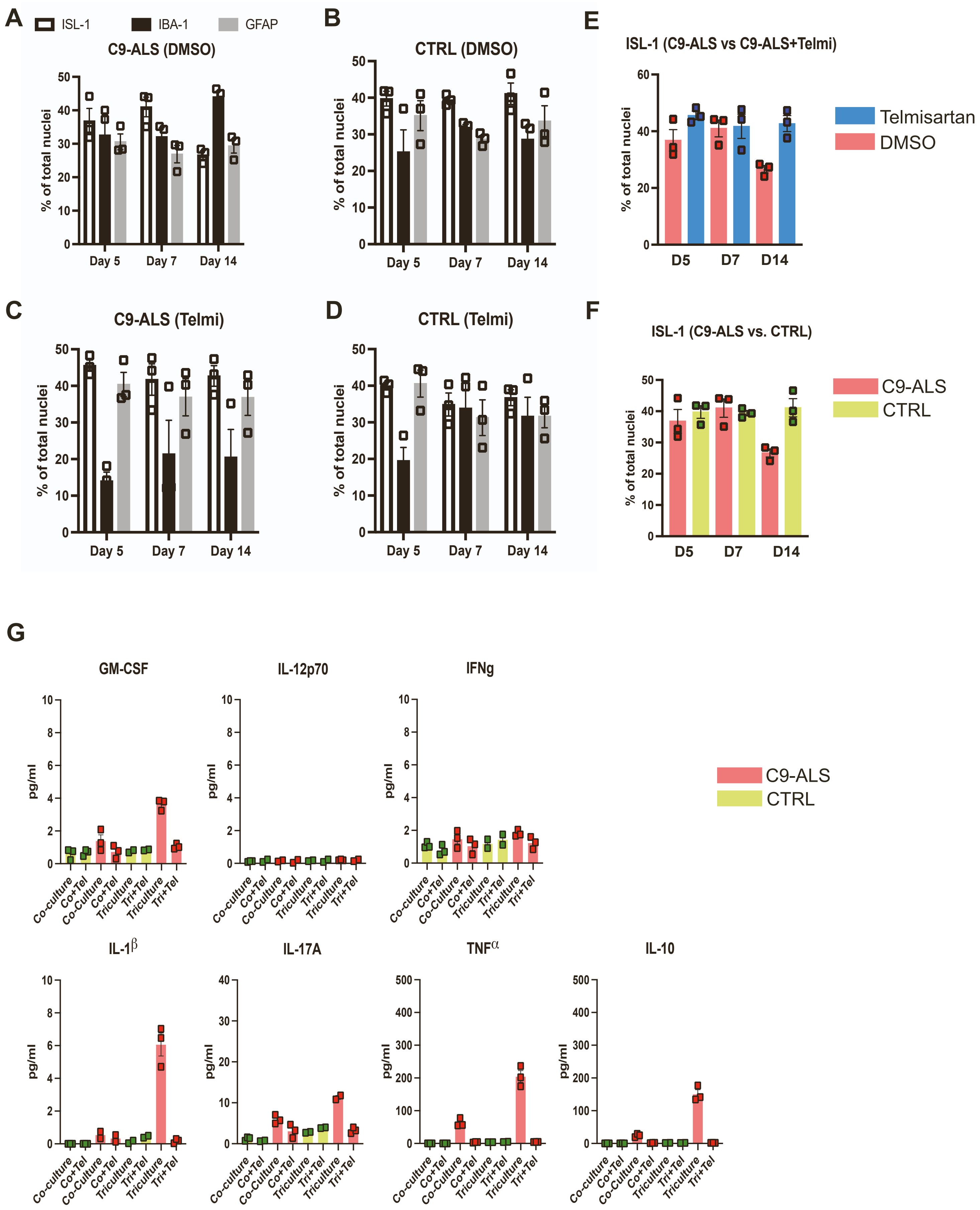

Figure S4. Time-Dependent Effects of Telmisartan on Cell Proportions in C9-ALS Spinal Microtissues – related to Fig. 4

(A)-(D) The bar graphs depict the proportions of IBA1+ microglia, GFAP+ astrocytes, and ISL-1+ spinal motor neurons (MNs) in C9-ALS (red) and control (CTRL, green) spinal microtissues treated with telmisartan (Telmi) over 14 days. The data is shown for Day 5 (D5), Day 7 (D7), and Day 14 (D14).

(E) ISL-1 proportion comparisons between C9-ALS SM +DMSO vs. C9-ALS SM + telmisartan represented as % nuclei of total.

(F) ISL-1 proportion comparisons between C9-ALS SM +DMSO vs. CTRL SM + DMSO represented as % nuclei of total.

(G) Bar graphs depicting the effect of telmisartan on the levels of GM-CSF, IL-10, IL-1β, TNFα, and IL-17A from the supernatants of C9-ALS co- and tri- cultures at 336 hours.

The data presented in this figure were generated using confocal microscopy on whole-mount stained SMs. The quantifications in these panels are based on the z-stack imaging, which captures the three-dimensional architecture of the SMs. The analysis paradigms are described in the Methods section in detail. N = 3 technical replicates, generated using 2 differentiations from 1 C9-ALS line and its isogenic control. Data are represented as Mean ± SEM.
